# Widespread aquifer depressurization after a century of intensive groundwater use in USA

**DOI:** 10.1126/sciadv.adh2992

**Published:** 2023-09-13

**Authors:** Annette Hilton, Scott Jasechko

**Affiliations:** Bren School of Environmental Science and Management, University of California, Santa Barbara, CA 93106, USA.

## Abstract

Water supplies for household use and irrigated agriculture rely on groundwater wells. When wells are drilled into a highly pressurized aquifer, groundwater may flow up the well and onto the land surface without pumping. These flowing artesian wells were common in the early 1900s in the United States before intensive groundwater withdrawals began, but their present-day prevalence remains unknown. Here, we compile and analyze ten thousand well water observations made more than a century ago. We show that flowing artesian conditions characterized ~61% of wells tapping confined aquifers before 1910, but only ~4% of wells tapping confined aquifers today. This pervasive loss of flowing artesian conditions evidences a widespread depressurization of confined aquifers after a century of intensive groundwater use in the United States. We conclude that this depressurization of confined aquifers has profoundly changed groundwater storage and flow, increasing the vulnerability of deep aquifers to pollutants and contributing to land subsidence.

## Teaser

Natural aquifers were inflated with ‘artesian’ pressure, but a century of groundwater pumping in the US has deflated its aquifers

## INTRODUCTION

Groundwater sustains food systems and provides drinking water to millions of people in the US. Agricultural and economic development in the 1800s and early 1900s relied on abundant water supplies provided by “flowing artesian wells,” defined as wells where groundwater flows to the land surface without pumping (*1*). These flowing artesian wells were used to irrigate farmlands (*2*), provide safe and inexpensive drinking water (*3*), and support businesses (*4*, *5*). Flowing artesian conditions indicate that there is a sufficiently high hydraulic head for upward-oriented groundwater flow. Upward-oriented groundwater flows may protect deep drinking water from downward transport of surface-borne pollutants (*6*). The loss of flowing artesian conditions and upward-oriented groundwater flows demonstrate depressurization of an aquifer. Depressurization can change groundwater flow patterns over large areas, affecting the solute distributions in aquifers (*7*). Depressurization can also alter an aquifer’s skeletal structure by the compression and compaction of confining units, leading to land subsidence and the loss of groundwater storage, especially in aquifer systems with fine-grained unconsolidated sediments (*8*).

**Fig. 1.**
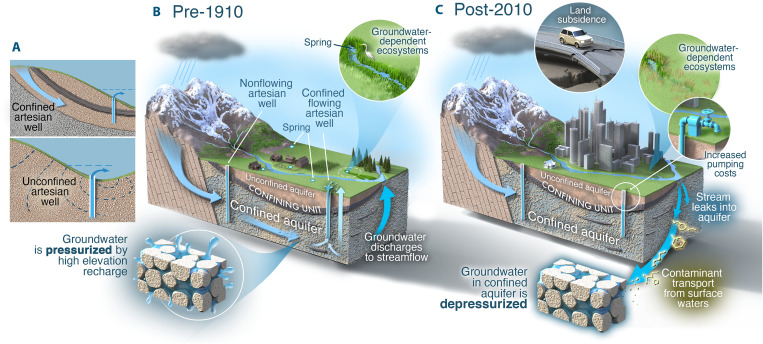
Schematic representation of flowing artesian conditions and groundwater flow and storage in confined aquifers before the year 1910 and after 2010. (**A**) Conceptual framework depicting how flowing artesian conditions can arise in wells drilled into unconfined aquifers (top) and confined aquifers (bottom image) [after (*84*)]. Our analyses focus on the prevalence of flowing artesian conditions in confined aquifers (i.e., top image). (**B**) Groundwater flow and storage in confined aquifers, as well as concepts and processes relevant to this study. Blue arrows depict groundwater flow directions. Before the year 1910 [i.e., (B) pre-1910], we depict a confined aquifer that is pressurized by recharge at relatively high elevations [see blue arrow in mountainous area on the left side of (A) depicting recharge]. Wells drilled into this confined aquifer have flowing artesian conditions because the hydraulic head in the confined aquifer exceeds the land surface elevation. Farther along the groundwater flow pathway, flow is upward-oriented and groundwater discharges to surface water systems. (**C**) After the year 2010 [i.e., (C): post-2010], we depict a confined aquifer that has been depressurized by decades of groundwater withdrawals. Wells drilled into this confined aquifer no longer exhibit flowing artesian conditions due to the depressurization of the confined aquifer. Confined aquifers are more susceptible to rapid and high-magnitude water level declines in response to groundwater withdrawals (relative to withdrawals from unconfined aquifers) because of the relatively small storage coefficients that characterize confined aquifers. In its depressurized state, the post-2010 aquifer system is characterized by groundwater flow that is downward-oriented. Ramifications of the depressurization of the confined aquifer include land subsidence, decline of groundwater discharge to surface water systems, and increased pumping costs. With depressurization, there is also the potential for surface-borne contaminant transport into deeper aquifer units as well as biochemical alterations to occur in the subsurface, resulting in contaminants of geogenic origin [see labeled bubbles surrounding (C)]. Figure design by Victor O. Leshyk.

Flowing artesian conditions can arise in wells that tap unconfined aquifers or those that tap confined aquifers (Fig. 1A). In some unconfined aquifers, gravity-driven groundwater flow in areas with uneven topography can lead to upward-oriented flow in valleys (*9*), creating flowing artesian conditions in wells that are sufficiently deep (Fig. 1A) (*10*, *11*). In some confined aquifers, high-elevation recharge and overlying aquitards can lead to potentiometric surfaces that lie above land surfaces, creating flowing artesian conditions in wells that tap such confined aquifers [for reviews see (*1*, *12*); Fig. 1A]. We reviewed groundwater studies that were published more than 100 years ago and focus on flowing artesian conditions in wells that tap confined aquifers. Our literature review highlights that flowing artesian conditions were more widespread in the 1800s and early 1900s than they are today, with dozens of works published in the early 1900s reporting artesian wells (*13*).

Despite their importance to groundwater-dependent ecosystems and human water access, no continent-wide study has quantified how prevalent flowing artesian conditions once were or how they have changed over the last century. Here, we show that the prevalence of flowing artesian conditions has declined substantially in confined aquifers over the last one hundred years, revealing substantial aquifer depressurization in the US. Our literature review reveals that flowing artesian conditions began to decline early in the twentieth century, motivating us to find records that predate these declines (see the “Groundwater withdrawals and the disappearance of flowing artesian wells” section).

To do so, we compiled thousands of water level measurements from US Geological Survey reports published in the early 1900s and compared these measurements to modern well water level measurements. We developed two complementary analyses to quantify change over time in flowing artesian conditions at the regional and continental scale. Our dual-method approach (i.e., study at both regional-scale and at continental scale) allows us to (i) examine the loss of flowing artesian conditions in individual aquifer units for aquifer systems, where three-dimensional (3D) hydrostratigraphic data are available, and (ii) estimate depth to confined conditions in a diverse array of aquifers across the US where hydrostratigraphic data are not available. The century-long timespan of our analysis, which is more extensive than the time intervals considered by most studies of groundwater levels [cf. (*14*)], enables us to demonstrate how markedly hydraulic heads have changed in the face of extensive groundwater development.

## RESULTS

### Prevalence of flowing artesian wells decreases over time in regional aquifer systems

**Fig. 2.**
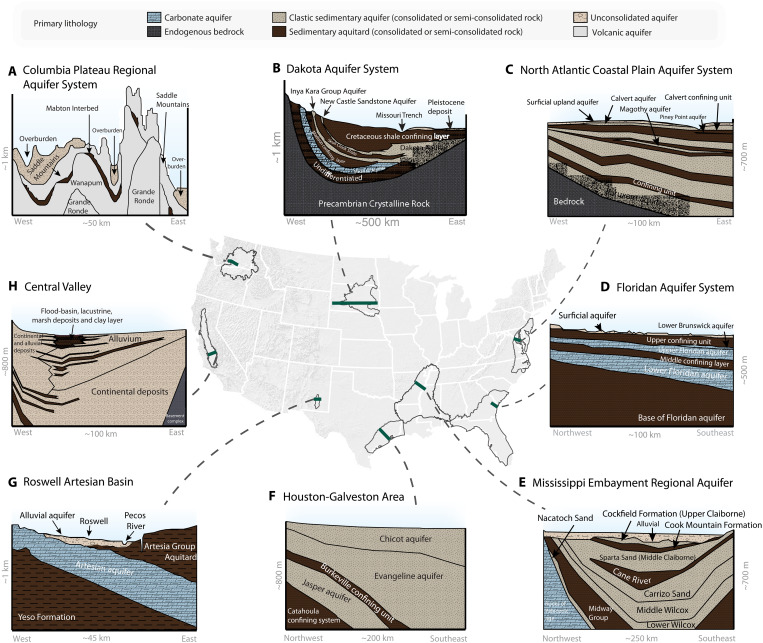
Hydrogeologic cross sections of eight regional aquifer systems. These aquifer systems exemplify a diverse array of lithologies, including carbonate and volcanic rocks, as well as sedimentary aquifers and aquitards. (**A**) The Columbia Plateau Regional Aquifer System is a basaltic aquifer system overlain by a surficial aquifer unit. (**B**) The Dakota Aquifer System consists of clastic and carbonate sedimentary rocks overlying endogenous bedrock. (**C**) The North Atlantic Coastal Plain Aquifer System is a multilayered sedimentary aquifer system overlying bedrock. (**D**) The Floridan Aquifer System consists of carbonate aquifers with confining and semiconfining sedimentary units. (**E**) The Mississippi Embayment Regional Aquifer System is characterized by an alluvial surficial aquifer overlying layered clastic sedimentary aquifers and aquitards. (**F**) The Houston-Galveston Area—within the broader Gulf Coast Regional Aquifer System—consists of layered clastic sedimentary formations that include confining fine-grained sediments. (**G**) The Roswell Artesian Basin is a carbonate aquifer system overlain by an alluvial aquifer. (**H**) The Central Valley is an unconsolidated clastic aquifer, where lenses of fine-grained sediments act as local or regional aquitards. Cross sections (A to H) are based on descriptions and figures presented in (*24*, *85*–*91*). We acknowledge M. GebreEgziabher for help digitizing many of the hydrogeologic cross sections. See section S1 for detailed descriptions of hydrostratigraphy; see figs. S1 to S8 for enlarged versions of each cross section.

**Fig. 3.**
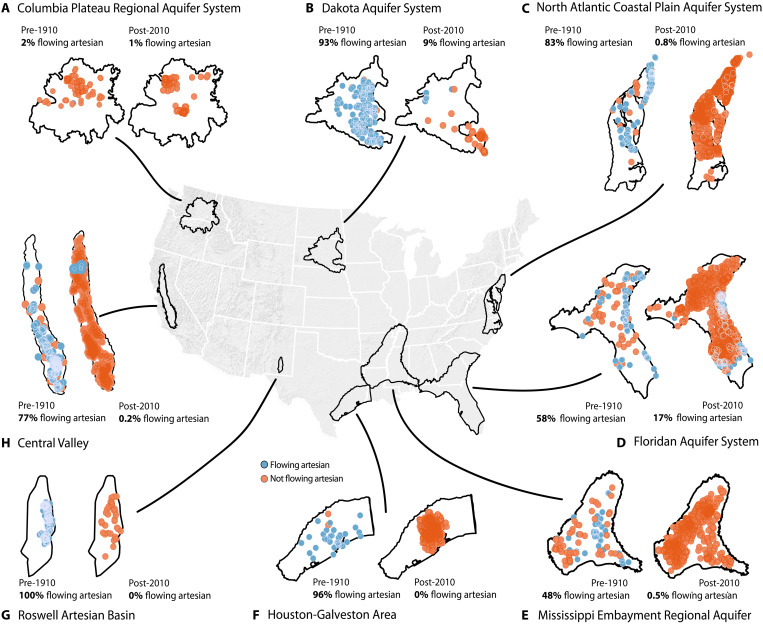
The prevalence of flowing artesian conditions among wells tapping confined aquifers before the year 1910 versus after the year 2010 in eight regional aquifer systems. (**A** to **H**) Individual maps of wells that tap confined conditions (see Materials and Methods). Blue dots represent flowing artesian wells, and orange dots represent nonflowing wells. The outlines of the orange and blue points are displayed atop (i.e., in front of) the filled circles to aid visualization where points are densely distributed. The maps on the left-hand side (labeled pre-1910) present well observations made before the year 1910, whereas the map on the right-hand side (labeled post-2010) presents observations made after the year 2010. In all eight of our studied aquifer systems, the proportion of wells drilled into confined aquifers exhibiting flowing artesian conditions declined over time. In some cases, flowing artesian conditions characterized nearly most wells before 1910 but characterize less than 1% of wells in our post-2010 dataset [e.g., (C), (D), (F), (G), and (H)]. In other cases, flowing artesian conditions were rare even before the year 1910, highlighting that not all confined aquifers produce flowing artesian conditions in wells even a century ago [e.g., (A)]. See section S4 and table S9 for details.

We characterized spatial distributions of flowing artesian wells for eight aquifer systems under two distinct time intervals: (a) well water level measurements made before the year 1910 (“pre-1910”) and (b) well water level measurements made more recently than 2010 (“post-2010”). These eight aquifer systems were selected for study because they are (i) geographically dispersed (Fig. 2), (ii) geologically diverse (Fig. 2 and section S1), (iii) representative of varying groundwater withdrawals (*15*) (section S2), and (iv) previously studied, so that 3D hydrostratigraphic data were available (section S3). Critically, we show that, in all eight study areas, the proportion of wells exhibiting flowing artesian conditions declined over the past century (Fig. 3).

In six of the eight regional aquifer systems, we find that flowing artesian conditions were common a century ago (48 to 100% of wells in our pre-1910 dataset were flowing artesian). Today, however, fewer than 10% of wells are flowing artesian (in our post-2010 dataset; Fig. 3). These six systems are (i) the Dakota Aquifer System (where the proportion of wells that exhibit flowing artesian conditions declined from 93 to 9% over the past century; Fig. 3B), (ii) the North Atlantic Coastal Plain Aquifer System (declined from 83 to 0.8% over the past century; Fig. 3C), (iii) the Mississippi Embayment Regional Aquifer (declined from 48 to 0.5% over the past century; Fig. 3E), (iv) the Houston-Galveston area within the broader Gulf Coast Aquifer System (declined from 96 to 0% over the past century; Fig. 3F), (v) the Roswell Artesian Basin in southeast New Mexico (declined from 100 to 0% over the past century; Fig. 3G), and (vi) the California Central Valley (declined from 77 to 0.2% over the past century; Fig. 3H; for further details see tables S8 to S17).

Flowing artesian wells were also common over a century ago in the Floridan Aquifer System (58% of wells in our pre-1910 dataset). Contrasting the four above aquifer systems, the Floridan Aquifer System has retained an ability to support flowing artesian conditions at present day (17% of wells exhibit flowing artesian conditions in our post-2010 dataset; Fig. 3D). The wells that exhibit flowing artesian conditions in the Floridan Aquifer System in our post-2010 dataset are concentrated along the coasts, whereas flowing artesian wells are nearly nonexistent farther inland (Fig. 3D). However, we note that the proportion of wells exhibiting flowing artesian conditions declined considerably from pre-1910 (58% of wells) to present (17% in our post-2010 dataset).

Unlike the other regional aquifer systems examined, flowing artesian wells were not common in the Columbia Plateau Regional Aquifer System before 1910. In our pre-1910 dataset, just 2% of wells that tap confined aquifers exhibit flowing artesian conditions, and 1% of wells are flowing artesian in our post-2010 dataset (Fig. 3A). The pre-1910 Columbia Plateau Regional Aquifer System data demonstrate that not all confined aquifers supported flowing artesian wells a century ago.

Because detailed 3D hydrostratigraphic data are available for all eight systems, we examined how the prevalence of flowing artesian conditions changed between our pre-1910 and post-2010 dataset for individual aquifer units. Examining individual aquifer units allows us to calculate the change over time among wells that tap different geologic units that are stacked on top of each other. We find that the prevalence of flowing artesian conditions changed substantially for some aquifer units but not others, even where these units underlie the same land area and exist within the same aquifer system. We used our well water level observations to estimate hydraulic heads of individual aquifer units within the eight regional systems for pre-1910 and post-2010 time intervals (figs. S11 to S18). We conclude that some individual aquifer units have depressurized over the past century more than others, even within the same aquifer system.

### Flowing artesian conditions extinguished in many diverse aquifer systems across the US

**Fig. 4.**
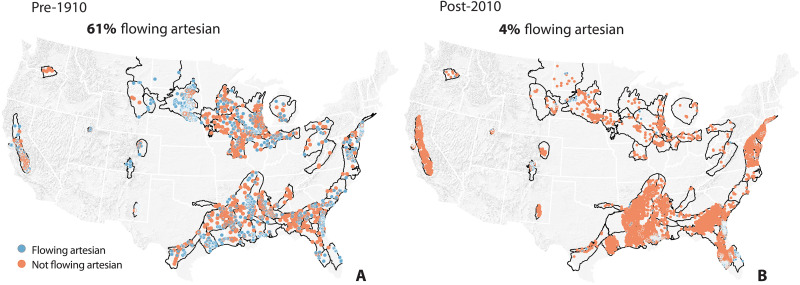
Comparison of the prevalence of flowing artesian conditions among wells tapping confined aquifers before the year 1910 versus after the year 2010 in *n* = 62 aquifer systems. Blue points represent flowing artesian wells (i.e., where the nonpumping potentiometric surface lies above the land surface). Orange points represent nonflowing artesian wells (i.e., where the nonpumping water level lies below the land surface). The outlines of the orange and blue points are displayed atop the filled circles (i.e., at the “front”) to aid visualization where points are densely distributed. (**A**) Among our dataset of well water level measurements made before the year 1910 (i.e., pre-1910), more than half (61%) of wells tapping a confined aquifer exhibit flowing artesian conditions [*n* = 1653 flowing artesian wells among *n* = 2703 total wells displayed in (A)]. (**B**) Among our dataset of well water level measurements made more recently than 1 January 2010 (i.e., post-2010), only 4% of wells tapping a confined aquifer exhibit flowing artesian conditions [*n* = 381 flowing artesian wells among *n* = 9644 total wells displayed in (B)]. Some of the aquifer systems presented here were also presented in our examination of 3D hydrostratigraphic data (i.e., presented in Fig. 3); for a comparison of results presented in Fig. 3 and this figure (i.e., Fig. 4), see table S21.

**Fig. 5.**
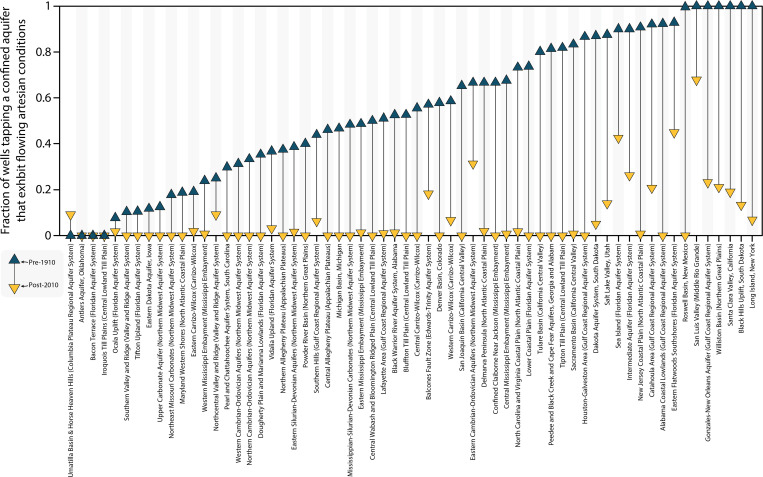
A century of change in the proportion of wells which exhibit flowing artesian conditions in 62 US aquifer systems. Each column in the plot presents data for one aquifer system (see aquifer system titles along the bottom axis of the figure). Dark-blue upward-pointing triangles mark the proportion of wells, which exhibit flowing artesian conditions in our pre-1910 dataset (i.e., well water level measurements made before 1 January 1910 in wells sufficiently deep to tap confined aquifers). Yellow downward-pointing triangles mark the proportion of wells which exhibit flowing artesian conditions in our post-2010 dataset (i.e., well water level measurements made more recently than 1 January 2010 in wells sufficiently deep to tap confined aquifers). A thin black line drawn between the two triangles depicts the magnitude of the difference in the proportion of wells, which exhibit flowing artesian conditions between the pre-1910 and the post-2010 dataset (i.e., the length of the thin black line connecting the triangles represents the difference in the fraction of wells tapping a confined aquifer that exhibit flowing artesian conditions for pre-1910 versus post-2010 datasets). We only analyze wells that were classified as tapping a confined aquifer on the basis of their total depth and the depth distributions of wells defined as tapping a confined aquifer by the US Geological Survey (see Materials and Methods; table S19). For further details on each aquifer system, see table S20.

To examine how flowing artesian conditions have varied over the past century in aquifer systems where we lack detailed 3D hydrostratigraphic data (i.e., aquifers beyond those in Fig. 3), we analyzed depth profiles of wells that the US Geological Survey has classified as tapping aquifers that exhibit either unconfined conditions or confined conditions. We estimated a “depth to confined conditions” based on these US Geological Survey data (table S19). We then mapped wells that were sufficiently deep to tap confined aquifers (i.e., deeper than our estimated depth to confined conditions) and analyzed the prevalence with which these wells exhibit flowing artesian conditions. Sixty-two (*n* = 62) aquifer systems have sufficient well water level data in our pre-1910 and post-2010 datasets for analyses [where “sufficient” is defined as at least *n* = 4 wells in both our pre-1910 dataset and at least *n* = 4 wells in our post-2010 dataset; see the “Identifying wells that tap confined aquifers across the US (Figs. 4 and 5)” section].

Across the continental US (*n* = 62 aquifer systems), we find that the majority of wells tapping a confined aquifer exhibited flowing artesian conditions before the year 1910 (*n* = 1653 flowing artesian wells among a total of *n* = 2703 wells; i.e., 61% of wells; Fig. 4A). By contrast, among measurements made more recently than the year 2010, we find that only 4% of wells tapping a confined aquifer exhibited flowing artesian conditions (*n* = 381 flowing artesian wells among a total of *n* = 9644 wells; Fig. 4B). By juxtaposing pre-1910 and post-2010 datasets, we demonstrate a clear and pervasive loss of flowing artesian conditions over the past century among US groundwater wells that tap confined aquifers (Fig. 5). When we examine each aquifer system individually, the loss of flowing artesian conditions is even more apparent. Specifically, among all aquifer systems in which our pre-1910 dataset contains at least one flowing artesian well (*n* = 58 aquifer systems), the proportion of wells exhibiting flowing artesian conditions declined over the century in every one (i.e., all *n* = 58) of these aquifer systems (Fig. 5). Further, in half of these *n* = 58 aquifer systems, we find no evidence of flowing artesian conditions in any of the wells in our post-2010 dataset, suggesting a complete disappearance of flowing artesian conditions over the past century (i.e., in *n* = 29 of *n* = 58 aquifer systems). Examples of these aquifer systems include the Denver Basin (where the proportion of wells that exhibit flowing artesian conditions declined from 58 to 0% over the past century), the Peedee and Black Creek and Cape Fear Aquifers of North and South Carolina (declined from 81 to 0% over the past century), the Tipton Till Plain of Indiana (declined from 82 to 0% over the past century), the Alabama Coastal Lowlands of Alabama (declined from 92 to 0% over the past century), and the Confined Claiborne near Jackson, Mississippi (declined from 67 to 0% over the past century; see table S20 for specific details on these and other aquifer systems).

Our compiled US well water observations lead us to two main findings: (i) natural hydrogeologic conditions—i.e., climate, topography, and geology—pressurized many confined aquifers to a sufficient extent to cause the first wells drilled into these aquifers to exhibit flowing artesian conditions, and, critically, (ii) that, after a century of extensive groundwater withdrawals across the US, confined aquifers have been depressurized so extensively that they seldom support flowing artesian conditions in wells screened within them.

There are areas where flowing artesian conditions have declined over the past century but still exist today. Examples of such cases include the San Luis Valley of southern Colorado (where the proportion of wells that exhibit flowing artesian conditions declined from 100 to 68% over the past century), the Eastern Cambrian-Ordovician Aquifers of Wisconsin (declined from 67 to 31% over the past century), the Salt Lake Valley (declined from 88 to 14% over the past century), the Gonzales-New Orleans Aquifer (declined from 100 to 23% over the past century), and Long Island (declined from 100 to 7% over the past century). Despite the retention of some flowing artesian conditions in these examples, there is a widespread loss of flowing artesian conditions across the continental US (only *n* = 381 flowing artesian wells among a total of *n* = 9644 wells exhibit flowing artesian conditions in our post-2010 dataset). This loss demonstrates that confined aquifers have been substantially depressurized over the past century (Figs. 4 and 5 and table S20).

## DISCUSSION

### Groundwater withdrawals and the disappearance of flowing artesian wells

Our compiled well water level observations demonstrate the widespread decline in the prevalence of flowing artesian conditions in the US over the last century. Long-term groundwater withdrawals from confined aquifers have been implicated as the primary reason that artesian wells stopped flowing in the Los Angeles Basin [prevalence of flowing artesian conditions reduced substantially by ~1905 (*16*, *17*)], southeastern Michigan [many wells stopped flowing by ~1905 (*18*)], northeastern Texas [many wells stopped flowing by ~1894 (*19*)], and the Dakota Aquifer System [many wells stopped flowing by ~1910 (*12*)]. Although we lack adequate local-scale groundwater withdrawal and hydrogeologic data for causal analyses, historic groundwater withdrawals (*15*) are often acknowledged (*20*) to be the primary driver behind widespread loss of flowing artesian conditions over the past century that we demonstrate here (Figs. 3 to 5).

Present-day US groundwater withdrawals (*21*) (~110 km^3^/year) comprise ~10% of global withdrawals (*22*) and increased substantially from 1950 to present-day [see table 14 within (*21*)]. Groundwater withdrawals from confined aquifers—the focus of this study—make up a portion of these withdrawals, although the exact share of total US groundwater withdrawals that derive from confined aquifers is not known. Confined aquifers are vulnerable to large and rapid reductions in hydraulic head per unit of withdrawn groundwater because of their low storativities [e.g., see discussion of the Cambrian-Ordovician Aquifer System in (*4*)]. We compiled groundwater withdrawal time series for four of our regional aquifer systems but did not find a close correlation between cumulative groundwater withdrawals and modern-day prevalence of flowing artesian wells, highlighting that other factors (e.g., aquifer storativity) are also important influences on the modern-day presence of flowing artesian wells.

Once constructed, flowing artesian wells can continue to extract groundwater from the confined aquifer until either the well is capped or the hydraulic head declines below the top of the well casing and the artesian well stops flowing. In some historical cases, flowing artesian wells went on flowing uninterrupted for long time intervals (*4*, *17*, *18*, *23*–*25*), prompting the US Geological Survey (*17*) to label flow from artesian wells as “careless” and “misused.” For example, uncontrolled flow from an artesian well in 1899 formed a shallow lake 100 feet across in Michigan (Monroe County) (*18*), and similar issues even prompted state law to prevent the loss of artesian flows in Michigan as early as 1905 (*18*). Combating the loss of groundwater resources and the harmful effects of uncontrolled flow from abandoned flowing artesian wells continues to be prioritized in the US and globally [e.g., the St. Johns River Water Management District of the Floridan Aquifer System (*26*, *27*); Coachella Valley Groundwater Basin, California (*28*); the Jordan Valley of Jordan (*29*)].

These examples, as well as the large cumulative groundwater withdrawals estimated by the US Geological Survey (*21*), support the hypothesis that groundwater withdrawals have driven the pervasive loss of flowing artesian conditions over the past century documented here. Regardless of the primary drivers, our work shows that the depressurization of artesian aquifers is not isolated to a few local areas but is, instead, a continental-scale phenomenon.

### Ramifications of widespread depressurization of artesian aquifers

The depressurization of artesian conditions in confined aquifers has ramifications for (i) water access and socioeconomic development, (ii) contaminant transport, and (iii) land subsidence (Fig. 1B). (i) Flowing artesian wells have been an important catalyst in societal and economic development (*1*, *19*, *30*, *31*). Socioeconomic consequences of the loss of flowing artesian conditions can include the loss of intrinsic connectivity of communities to groundwater (*18*, *19*, *32*) and increased energy requirements to access groundwater (*17*). The low cost and availability of groundwater sourced from flowing artesian wells motivated settlement in parts of the US (*3*, *5*, *28*). These settlements relied on flowing artesian wells for irrigating croplands (*33*, *34*) and providing drinking water (*35*), particularly where surface waters were scarce or contaminated [e.g., Gulf Embayment in Mississippi (*3*) and San Joaquin Valley in California (*2*)]. The loss of flowing artesian conditions imposes an increase in the energy required to lift the well water, and therefore cost required to access groundwater. Some towns in the US prospered for years by using artesian wells as a resource for public pools (*5*), bathhouses (*36*–*38*), ice-making (*39*), firefighting (*18*), power generation (*4*), and many other purposes for private citizens [ponds stocked with fish (*5*, *18*) and preserving food (*5*)] or industries [dairy houses (*18*) and laundry businesses (*5*)]. In many cases, the artesian wells eventually stopped flowing, spurring communities reliant on flowing artesian wells to adapt [e.g., Waco in Texas (*37*, *40*)] or even leave [e.g., Somerville County in Texas (*5*)]. Here, we show that the prevalence of flowing artesian wells has declined substantially across the US, revealing once-prosperous communities that were forced to adapt in response to the loss of flowing artesian conditions in wells.

(ii) Contamination of deep groundwaters of anthropogenic and geogenic origin are possible ramifications of confined aquifer depressurization. Depressurization alters groundwater flow patterns, including changes in vertical hydraulic gradients (i.e., hydraulic potential driving groundwater movement). This change in vertical hydraulic gradients can slow natural upward-oriented flows or even reverse groundwater flow directions (*6*). Where vertical hydraulic gradients have reversed, downward movement of surface-borne contaminants into deeper aquifers can threaten deep groundwater quality (*6*), a concern that was raised as early as 1905 (*18*). Recent research (*41*) has demonstrated the presence of “modern groundwater”—which is more likely to bear surface-borne contaminants (*42*, *43*)—in many wells that tap confined aquifers in the US. It is also possible for depressurization to induce geogenic contamination of deep groundwater by biochemical alterations. For example, the depressurization of confined aquifers due to groundwater withdrawals can lead to arsenic contamination, as groundwater from fine-grained confining units flows into the confined aquifer with aqueous-arsenic or arsenic-mobilizing-solutes [e.g., dissolution of iron oxides or expulsion of reactive carbon (*44*, *45*)]. Where aquifer units are connected by well screens, mixing of chemically distinct groundwater can contaminate shallower groundwater systems (*46*).

(iii) The depressurization of confined aquifers can induce land subsidence, harming economies via damage to infrastructure. Specifically, reductions in hydraulic heads in unconsolidated aquifer systems with substantial clay content can result in land subsidence as confining units are compacted [e.g., Galveston, Texas (*47*); Santa Clara Valley, California (*25*); Savannah, Georgia (*48*); Mexico City, Mexico (*6*); Venice, Italy (*49*); Tokyo, Japan (*50*); South Bengal Basin (*51*)]. The land areas that are most vulnerable to land subsidence tend to be alluvial basins and coastal plains (*8*). Many of our study areas have been identified as being highly susceptible to land subsidence in a recent global study (*8*), including some of the aquifer systems where we identify widespread reductions in the prevalence of flowing artesian wells over the past century (e.g., Central Valley, Houston-Galveston area, North Atlantic Coastal Plain Aquifer System). In California’s Central Valley—where we show that flowing artesian conditions were widespread in the early 1900s but have since disappeared (Fig. 3H)—lands have subsided by as much as 9 m since the 1920s, causing billions of dollars in damage to infrastructure (*52*).

### Geologic, climate, and anthropogenic impacts on artesian conditions

Flowing artesian wells can occur in wells that tap unconfined or confined aquifers (Fig. 1A) (*9*, *12*). In this study, we focus on flowing artesian wells that tap a confined aquifer. To explore the potential influence of environmental and anthropogenic factors on the prevalence of flowing artesian conditions, we statistically examined the interactions between climatic (aridity index, mean annual precipitation) and anthropogenic (mean annual groundwater withdrawals) variables (tables S9 and S22). Our statistical analyses explain only a limited proportion of total variance in the prevalence of flowing artesian wells (in our post-2010 dataset), highlighting the complex set of factors that can influence the presence of flowing artesian wells. Some of the factors that may influence the prevalence of flowing artesian wells and their ability to sustain flowing artesian conditions over time include (i) geology, (ii) climate, and (iii) human intervention.

(i) Geologic conditions can play a critical role in generating flowing artesian conditions in wells and may determine how resilient flowing artesian conditions are to groundwater withdrawals. The confined aquifers that we study here vary widely in their geologic characteristics and include carbonate rock aquifers (e.g., Upper and Lower Floridan Aquifers in the Floridan Aquifer System; Artesia Group in the Roswell Artesian Basin), consolidated sandstone aquifers (e.g., Dakota Formation in the Dakota Aquifer System), and poorly consolidated alluvial basins (e.g., Tulare Basin in California’s Central Valley). The Floridan Aquifer System is an exemplar carbonate aquifer system that has retained some of its capacity to support flowing artesian wells over the last century (e.g., see flowing artesian wells in post-2010 dataset in Fig. 3D). The Roswell Artesian Basin has been called (*24*) a “rechargeable artesian aquifer” largely due to the presence of a carbonate aquifer at depth. It supported flowing artesian conditions for a portion of the last century (*24*), although our study indicates that flowing artesian conditions have all but vanished today (i.e., none of the wells in our post-2010 dataset exhibit flowing artesian conditions). The hydraulic properties of the varying geologic formations represented by our aquifer systems are critical to understand aquifer response to human interventions. Specifically, the capacity of an aquifer to release groundwater—as determined by storage properties inherent to a given geologic formation—is especially important when considering impacts of groundwater withdrawals on hydraulic head. Our statistical analyses (section S9) indicate that climatic variables and groundwater withdrawals alone are not sufficient to explain the observed proportion of wells exhibiting flowing artesian conditions. Although we lack data detailing aquifer storage properties for analyses, we highlight the importance of the hydraulic properties of aquifer systems and emphasize the potential value of a developing national database of hydraulic properties as an area of future work.

(ii) Climate can also be important to regional hydrogeologic conditions. In our statistical models, we did not find a strong relationship between either mean annual precipitation (*53*) or the aridity index (*54*) (annual precipitation divided by annual potential evapotranspiration) and the prevalence of flowing artesian conditions (tables S9 and S22). The lack of a strong statistical relationship between climate conditions and the prevalence of flowing artesian wells highlights that climate conditions are not the only factor influencing hydraulic heads in confined aquifers and their response to groundwater withdrawals. Nevertheless, we emphasize that climate conditions are an important aspect of all hydrogeologic systems and that our *n* = 62 aquifer systems span a wide array of climate conditions.

(iii) Human interventions including pumping and land use changes are important factors that may influence the presence of flowing artesian conditions (*55*, *56*). Unregulated and uncontrolled artesian flows from wells have long been recognized as detrimental to sustained groundwater use by US Geological Survey scientists and local citizens (*17*, *38*). Despite these warnings, flowing artesian conditions began to disappear as early as ~1894 (*19*). We do not observe a strong statistical relationship between annual groundwater withdrawals [as of 2015 (*41*)] and the prevalence of flowing artesian conditions in our study aquifers (tables S9 and S22); however, we lack adequate long-term groundwater withdrawal data to be confident that these two variables (total groundwater withdrawals over the past century and the change in the prevalence of flowing artesian conditions over the past century) are uncorrelated.

### The legacy of flowing artesian wells

Flowing artesian wells have served humanity for centuries (*12*). In the US, flowing artesian wells motivated settlements (*3*, *5*), supported livelihoods (*5*, *18*), and provided safe (*3*, *35*) and equitable (*3*) drinking water supplies. Although we present some of the earliest well water level observations available for the US (*3*, *57*–*60*), development of many aquifer systems began before the earliest measurements in our pre-1910 dataset (e.g., development via wells sunk in the mid-1800s). Our analysis reveals a substantial reduction in the prevalence of flowing artesian conditions across the US (~61% in our pre-1910 dataset, to ~4% in our post-2010 dataset; Fig. 4). We interpret our results as evidence for a widespread depressurization of confined aquifer systems.

This depressurization of US aquifers affected communities reliant on flowing artesian wells by impairing their access to water (*17*, *35*), including communities located where intensive groundwater withdrawals continue today (*61*–*65*). The decline in flowing artesian conditions may imply a reversal of groundwater flow directions from natural upward-oriented flow (i.e., suggested by widespread flowing artesian wells in the early 1900s) to the modern-era disrupted state where there is a greater likelihood for downward-oriented flow. These reversed vertical groundwater flow directions and depressurized aquifer systems have likely increased the potential for contamination in deep aquifers (*6*, *45*) and induced land subsidence (Fig. 1B) (*48*). Our analysis reveals that flowing artesian wells have been extinguished over a century of groundwater use in the US, affecting aquifer systems and humans that rely on artesian aquifers.

## MATERIALS AND METHODS

### Piezometric compilation and quality control

We compiled well water measurements throughout the continental US from three different sources by (i) downloading data from the US Geological Survey’s National Water Information System, (ii) digitizing water level measurements documented in US Geological Survey reports published in the early 1900s, and (iii) downloading piezometric data from two state agencies.

(i) We downloaded US Geological Survey National Water Information System (*66*) well water level data from a REST Web Service for the time range 1 January 1800 to 1 January 2022. We excluded monitoring wells where the dataset did not specify a well depth or where the dataset recorded a well depth of zero. We excluded water level measurements with non-numeric water levels (i.e., a blank entry) or where a flag was included in the database that suggested the water level measurement was compromised [i.e., we excluded measurements with one of the following codes in the field entitled “lev_status_cd”: “True value is below reported value due to local conditions,” “True value is above reported value due to local conditions,” “Measurement unable to be obtained due to local conditions,” “Frozen,” “Dry,” “Obstructed,” and “Pumping”; see Water Level Status Codes (*67*)].

(ii) We compiled thousands of well water level measurements reported in tables within US Geological Survey reports published in the early 1900s. We manually transcribed well water level measurements (*n* = 11,375) from five different reports published in the early 1900s (*3*, *57*–*60*). None of the early 1900s US Geological Survey reports that we consulted record the latitude and longitude of the well; the locations of these wells are provided in each report as a description (e.g., State, County, and City) or a township, range, and section. We estimated the descriptive locations of wells using a geocoding software [Geocode by Awesome Table (*68*)]. For further details pertaining to our data compilation and quality control procedures, see section S10. Last, we supplemented our own compilation by also analyzing well water level measurements made in California in the early 1900s by Mendenhall *et al.* (*2*), as digitized by Hansen *et al.* (*69*). The combination of the Mendenhall well water level data (*2*) (*n* = 3957), US Geological Survey well water measurements [described in (i) above; *n* = 1733], and our own compilation of well water level data from five early 1900s US Geological Survey reports (*n* = 4636 that met our criteria for analyses) sums to *n* = 10,326 early 1900s well water level measurements (see Data and Materials Availability statement).

(iii) We downloaded publicly available well water measurements from two state agencies to improve data coverage and supplement the US Geological Survey National Water Information System data. In California, we downloaded water level data for California’s Central Valley from the Groundwater Ambient Monitoring and Assessment Program (*70*) (downloaded 11 May 2022), the Department of Water Resources Periodic Groundwater Level Measurements (*71*) and Continuous Groundwater Level Measurements (*72*) (downloaded January 2022). In South Dakota, we downloaded state level piezometric data from the South Dakota Department of Agriculture and Natural Resources Observation Wells database (*73*) (downloaded 18 November 2021).

All piezometric records were constrained to two time periods: pre-1910 (measurements before the year 1910) and post-2010 (2010–2022). In many cases, in our post-2010 dataset, there were multiple water level measurements for a single monitoring well. In these cases, we calculated the median well water level for each unique well from all water level measurements over the 2010–2022 time period. All pre-1910 data have just one water level measurement per unique well, thus were not required to calculate a median water level. All water level data in our two statistical groups [i.e., (a) the measured water level for the pre-1910 dataset and (b) the median well water level for the post-2010 dataset] were used to create a binary classification for each well: flowing artesian (i.e., well water level is above the top of the land surface) or not flowing (i.e., water level is below the land surface). In the pre-1910 dataset, there is a variety of reporting for flowing artesian conditions (table S26). For the post-2010 dataset, water levels are reported as below land surface.

### Regional analysis and hydrostratigraphic data

We analyzed hydraulic heads in the confined portions of eight regional aquifer systems (Fig. 3): (a) Columbia Plateau Regional Aquifer System, (b) Dakota Aquifer System, (c) North Atlantic Coastal Plain Aquifer System, (d) Floridan Aquifer System, (e) Mississippi Embayment Regional Aquifer, (f) Houston-Galveston area within the broader Gulf Coast Aquifer System, (g) Roswell Artesian Basin, and (h) Central Valley.

To identify wells under confined aquifers, we used hydrostratigraphic spatial data for the regional aquifer systems (*74*–*80*). All hydrostratigraphic data were obtained from federal and state agencies as 3D raster data, except for (b) the Dakota Aquifer System, where such data did not exist. For the Dakota Aquifer System, we analyzed lithological logs from the South Dakota Department of Natural Resources Lithologic Logs Database (*81*) (downloaded 27 November 2021) to create a 3D representation of the top of the Dakota aquifer (section S3 and the “Limitations” section).

Well bottoms and well water levels were calculated using the US Geological Survey Digital Elevation Model (*82*) (⅓-arc-second) for each study period, consistent with the regional hydrostratigraphic raster data in the North American Datum of 1983. The well bottoms were used to determine confinement (table S27). Our criteria for confinement of wells for each aquifer system are described in table S27.

We also identified US Geological Survey wells designated as being drilled in a confined or unconfined aquifer unit that are located within the boundaries of our eight regional aquifer systems presented in Fig. 3 (such US Geological Survey classifications were only available for our post-2010 dataset). For aquifer systems that had sufficient wells with a US Geological Survey confined classification (*n* = 10), we compared the results of our hydrostratigraphic confining classification to these data and calculated the error rate of our analysis (i.e., how our method of classifying confined wells using hydrostratigraphic data matched the US Geological Survey’s own classification of confined wells) (table S28).

### Identifying wells that tap confined aquifers across the US

To go beyond boundaries of our eight regional aquifers (Fig. 3), we classified wells in other parts of the US as either confined or unconfined by analyzing wells that the US Geological Survey has classified as tapping unconfined or confined aquifers (*n* = 225,388 wells). We grouped these US Geological Survey wells by (a) the aquifer system that the well is located within [boundaries from the US Aquifer Database by GebreEgziabher *et al.* (*83*)], and by (b) the depth of the well (discrete depth intervals are defined at 10-m intervals from zero to 100 m and 20-m intervals for depths exceeding 100 m). Next, for each aquifer system, we calculated the percentage of wells within a given depth range (e.g., all wells with depths between 10 and 20 m) that have been classified as tapping confined aquifers by the US Geological Survey. Next, we calculated the shallowest “depth range” at which both of the following criteria are met: (i) 80% of wells with depths within the depth range are classified as confined, and (ii) more than 80% of wells with depths deeper than the depth range are classified as confined. We apply this estimate of the “depth to confined aquifers” across the entire aquifer system to our two water level datasets: (i.e., pre-1910 well water level measurements and post-2010 measurements) to identify wells that tap confined aquifers (table S19). For each time period, we compare only confined wells and present their flowing artesian versus nonflowing artesian conditions in Figs. 4 and 5.

### Statistical analyses

To examine potential factors that may influence the prevalence of flowing artesian conditions, we conducted a suite of statistical analyses. We examined the interactions between climatic [aridity index (*54*) and mean annual precipitation (*53*)] and anthropogenic [mean annual groundwater withdrawals for the year 2015 (*41*)] influences. Data compilation for these explanatory variables are detailed in section S8. Our statistical analyses included multiple hypotheses testing using generalized linear mixed models. Those models and the results are explained in detail in section S9.

### Limitations

Our analyses have several limitations. First, (i) our pan-US analyses (Figs. 4 and 5) use boundaries of aquifer systems as delineated by (*83*) to identify wells falling within each aquifer system and estimate the depth to confined aquifers for these individual systems. Although we only classify wells as tapping confined aquifers if 80% or more of all wells at (and deeper than) that depth are classified as confined by the US Geological Survey, this depth to confined conditions likely does not represent the inherent heterogeneity within each aquifer system. We recognize that estimating one depth to confined conditions for an entire aquifer system is an oversimplification, however, but it was a necessary oversimplification for us to examine aquifer systems that lack 3D hydrostratigraphic data (i.e., aquifer systems beyond the eight we present in Fig. 3). We examined the potential ramifications of our simplified method (represented in Figs. 4 and 5) by comparing them to our analyses of eight regional aquifer systems where we have 3D hydrostratigraphic data (i.e., results in Fig. 3). Results of our analysis of 62 aquifer systems across the US (Figs. 4 and 5) are largely consistent with our detailed analysis based on 3D hydrostratigraphic data in eight regional aquifer systems (Fig. 3).

Second, (ii) our regional-scale analyses (Fig. 3) analyze published 3D hydrostratigraphic data from the US Geological Survey and state agencies where available. For the Dakota Aquifer System, no such data existed. We compiled our own 3D hydrostratigraphic raster of the top of the Dakota Aquifer from lithological logs from the South Dakota Department of Natural Resources Lithologic Logs Database (section S3) (*81*). Our interpolated surface of the top of the Dakota Aquifer is therefore more uncertain than other data products that we analyzed here because our interpolation method may not capture important regional-scale geologic features (e.g., faults). For all eight regional systems, wells were only included in the analyses if they fell within the boundaries of the 3D hydrostratigraphic data extent.

Third, (iii) the pre-1910 water level data do not specify a well latitude and longitude; instead, the well locations are inexact text descriptions [for example, “Springfield, 4 ½ m. N. of” and “Albany, Whitehair farm, well No. 1” quoting (*57*)]. Our georeferencing approach and the way we accounted for additional location information (e.g.,“4 ½ m. N. of”) leads to uncertainty in the locations of these wells, meaning that some of our pre-1910 well locations are likely inaccurate. As our analyses focus mostly on expansive spatial scales, we expect that these inaccuracies do not alter our main finding: that there has been a pervasive loss of flowing artesian wells over the past century in the US.

Fourth, (iv) our analyses are only as representative as our data. The spatial distribution of US Geological Survey monitoring wells may not be sufficiently dense and even to adequately represent regional hydrogeologic conditions. For example, in the case of the Roswell Artesian Basin, we spoke with the superintendent of the Pecos Valley Artesian Conservation District. This individual indicated that US Geological Survey monitoring wells may not be in areas that currently support flowing artesian conditions; they noted that there was at least one well in their local monitoring network that still exhibits flowing artesian conditions today, although flowing artesian conditions of that well varied seasonally.

## Supplementary Materials

### This PDF file includes:

Supplementary Text Figs. S1 to S20 Tables S1 to S28 References
