# Supplementary material for: Widespread aquifer depressurization after a century of intensive groundwater use in USA
Source: Sci Adv. 2023 Sep 13;9(37):eadh2992. doi: 10.1126/sciadv.adh2992 (PMC11006208; doi:10.1126/sciadv.adh2992)
Supplement: Supplementary file 1 — Supplementary Text Figs. S1 to S20 Tables S1 to S28 References [file sciadv.adh2992_sm.pdf]

Science Advances
AAAS

Supplementary Materials for

Widespread aquifer depressurization after a century of intensive groundwater use in USA

Annette Hilton and Scott Jasechko

Corresponding author: Annette Hilton, ahilton@ucsb.edu

Sci. Adv. **9**, eadh2992 (2023)
DOI: 10.1126/sciadv.adh2992

This PDF file includes:

Supplementary Text
Figs. S1 to S20
Tables S1 to S28
References

S1. Hydrogeological conditions of our eight regional aquifer systems

Here we detail each regional aquifer system examined, providing a description, a cross-section, and hydraulic properties of the aquifer system where available.

(a) Columbia Plateau Regional Aquifer System

The Columbia Plateau Regional Aquifer System is an intermontane structural basin with volcanic and fluvial deposits. The system covers an area of 114,000 square kilometers in Washington, Oregon, and Idaho. The Columbia Plateau has been informally divided into four physiographic subprovinces: the Yakima Fold Belt, Blue Mountains, and Palouse subprovinces, and the Clearwater Embayment (*92*, *93*). Age of the sedimentary formations within the aquifer systems ranges from the Cenozoic to present (*94*). The primary hydrogeologic units in the Columbia Plateau Regional Aquifer System are the Overburden, Saddle Mountains, Mabton Interbed, Wanapum, Vantage Interbed, Grande Ronde, and Older Bedrock units (*85*). Generally, the system consists of basalt aquifers with permeable ‘interflow zones’ (i.e., the tops and bottoms of individual basalt deposits, and any sediment in between two stacked basalt deposits), separated by less permeable interiors (*85*). Sedimentary aquifers overlie the basalts in certain areas. The geologic materials and their hydrogeologic properties are highly heterogeneous in the Columbia Plateau Regional Aquifer System. The Overburden unit is characterized by diverse lithologies, grain sizes, and ages. In the confined, basalt hydrogeologic units, tens to hundreds of individual layered basalt flows may be present. The lava flows are flood basalts which are variable in thickness and extent, overall forming laterally extensive deposits across much of the system (*95*). Broadly, groundwater moves through the aquifer system from the uplands (topographic highs) to lowlands, primarily the Columbia River and its tributaries. Due to structural controls that compartmentalize much of the system, groundwater flow paths are relatively short compared to some other large aquifer systems. Major ridges in the Yakima Fold Belt are primary drivers of this compartmentalization (*85*).

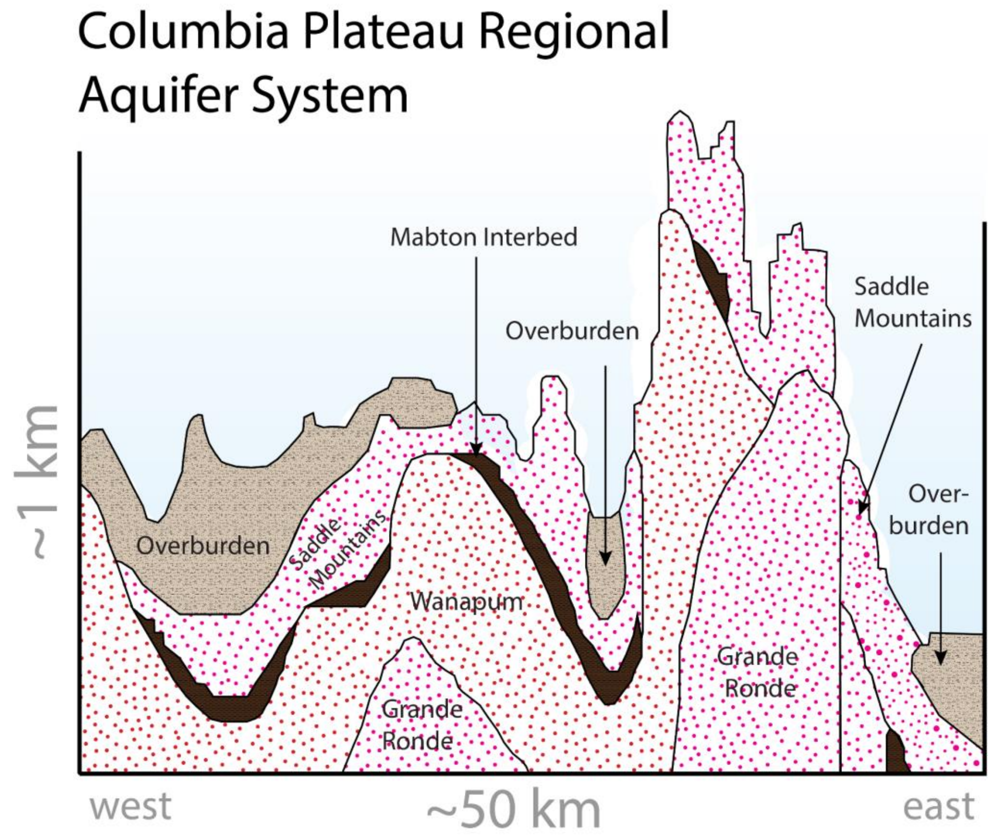

Fig. S1. This cross section shows one subarea within the broader Columbia Plateau Regional Aquifer System–the Yakima Basin–located in central Washington State. Overburden units (tan shades) overlie volcanic deposits (white shades with pink dots). The area is characterized by high topographic relief. The hydrogeologic cross section is based on Fig. 7b by (*85*); we acknowledge M. GebreEgziabher for their help digitizing the hydrogeologic cross section.

Table S1. Hydraulic properties of the Columbia Plateau Regional Aquifer System (*94*, *96*)**.**

**Hydrogeologic unit***	**Hydraulic conductivity (ft/d) (median)**	**Vertical hydraulic conductivity (ft/d) (median)**	**Specific storage (1/ft)**
Overburden	96	0.96	0.000025
Saddle Mountains interflow zones			0.0000025
Saddle Mountains flow interiors			
Saddle mountains (effective value)	14.4	0.003	
Mabton interbed	14.1	0.0001	
Wanapum interflow zones			
Wanapum flow interiors			
Wanapum (effective value)	13	0.001	
Vantage interbed	12.6	0.0001	
Grande Ronde interflow zones			
Grande Ronde flow interiors			
Grande Ronde (effective value)	10.2	0.0002	

*Hydraulic conductivity and vertical hydraulic conductivity values are reported in table 3 of (*94*) for the Columbia Plateau Regional Aquifer System (CPRAS) model by (*96*). Hydrogeologic units are defined by the CPRAS model for these parameters. For specific storage, values are reported by (*96*) for the surficial Overburden, and the combined Columbia River Basalt Group, which consists of the Saddle Mountains Basalt flow members and interbeds, the Mabton interbed, the Wanapum Basalt flow members and interbeds, the Vantage interbed, and the Grande Ronde Basalt member and interbeds. We recognize that these hydrogeologic units do not straightforwardly correlate (in all cases) to the hydrogeologic units used in our analysis. We use the hydrogeologic units reported in the geologic framework constructed by (*74*).

(b) Dakota Aquifer System

The Dakota Aquifer System covers ~171,000 square kilometers in South Dakota and extends into multiple adjacent states. The aquifer system in South Dakota is the focus in this study. The age of the aquifer systems' sedimentary units ranges from Paleozoic to Tertiary time. The primary hydrogeologic units in the Dakota Aquifer System are the Dakota sandstone (also referred to as the Dakota-Newcastle sandstone), the Inyan Kara Group, and the Madison Group. On the western side of the state, the Inya Kara Group aquifer and Newcastle Sandstone aquifer represent distinct aquifer units, but merge to form the Dakota sandstone in the eastern portion of South Dakota (*86*). The major aquifers are separated by low-permeability confining layers, which are mostly shale. Sometimes these confining layers contain minor aquifers, such as the Niobrara Formation and the Greenhorn Limestone. Recharge to the aquifer system occurs at the outcrop on the flanks of the Black Hills, which is a dome of crystalline Precambrian rocks located on the western side of the state (*97*). High elevations at the Black Hills diminish mostly uniformly to the east, broken by the valley of the Missouri River (Missouri Trench (*86*)). Groundwater generally flows from west to east.

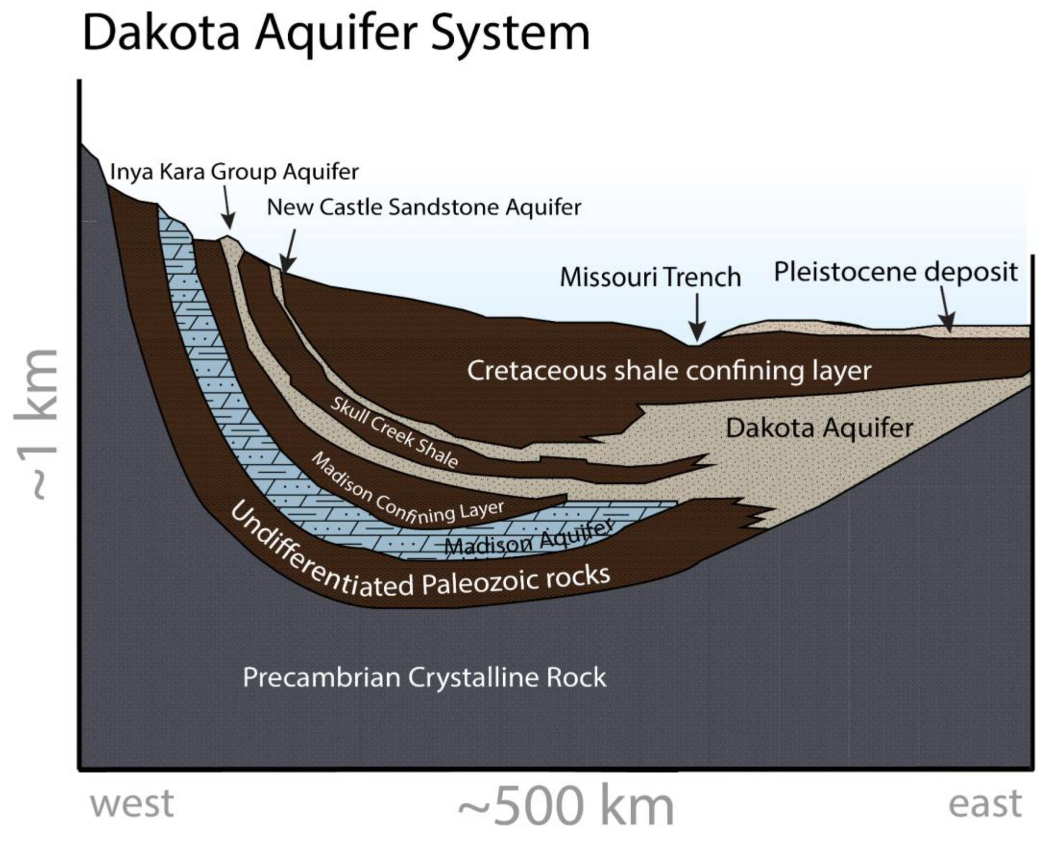

Fig. S2. Cross section of the Dakota Aquifer System of South Dakota. The hydrogeologic cross section is based on Fig. 1 by (*86*); we acknowledge M. GebreEgziabher for their help digitizing the hydrogeologic cross section. The cross section depicts a layered sedimentary aquifer system, with thick regional aquitards (e.g., Cretaceous shale) and a deep confined sandstone aquifer (the 'Dakota Aquifer').

Table S2. Hydraulic properties of the Dakota Aquifer System. Properties are best-fit parameter values used in the single Dakota aquifer model analysis (*86*)**.**

**Hydrogeologic unit**	**Hydraulic conductivity (ft/d)**	**Storage coefficient (dimensionless)**
Dakota-Newcastle sandstone	5.53	0.0001

(c) North Atlantic Coastal Plain

The North Atlantic Coastal Plain (NACP) covers 77,700 square kilometers, spanning from New York to the northeastern part of North Carolina. The aquifer system consists of a seaward dipping wedge of mostly unconsolidated sediments, including clay, silt, sand, and gravel (*98*). Sediment ages range from the Early Cretaceous to the Holocene. The western edge of the Coastal Plain is called the Fall Zone, where the sedimentary rocks of the North Atlantic Coastal Plain meet igneous and metamorphic rocks of the Piedmont Province. The primary hydrogeologic units of the North Atlantic Coastal Plain consists of ten regional aquifers and nine regional confining units. The continuity of these units vary in geographical space, with some units present in certain states within the NACP and not complete in others. The units include: the surficial aquifer, the Upper Chesapeake confining unit, the Upper Chesapeake aquifer, the Lower Chesapeake confining unit, the Lower Chesapeake aquifer, the Calvert confining unit, the Piney Point aquifer, the Nanjemoy-Marlboro confining unit, the Aquia aquifer, the Monmouth-Mount Laurel aquifer, the Matawan confining unit, the Matawan aquifer, the Magothy aquifer, the Potomac confining unit, the Potomac-Patapsco aquifer, the Potomac-Patuxent confining unit, and the Potomac-Patuxent aquifer (*98*). Recharge to the aquifer system mostly flows through the shallow unconfined aquifer and discharges directly to streams or coastal waters without reaching the confined aquifer units. In pre-development conditions, recharge was from precipitation as well as some groundwater and surface water flow eastward across the Fall Zone; even in pre-development conditions, simulations suggest that groundwater recharge to the confined aquifer system was small (<2% of total groundwater recharge; (*87*).

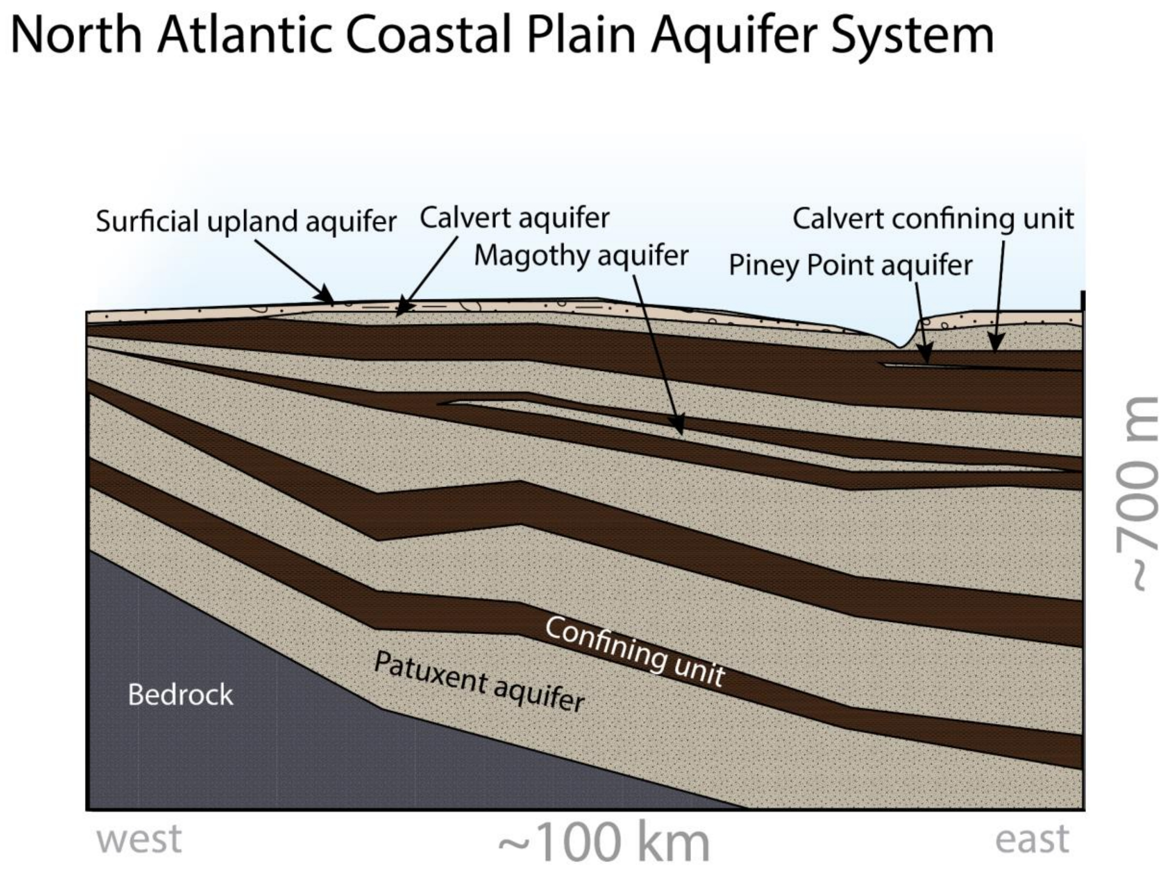

Fig. S3. Cross section of the North Atlantic Coastal Plain Aquifer System. The hydrogeologic cross section is based on Fig. 6 by (*87*); we acknowledge M. GebreEgziabher for their help digitizing the hydrogeologic cross section. The aquifer system consists of a suite of sedimentary formations, including both aquitards (dark brown layers in cross section) and aquifers (lighter tan layers in cross section).

Table S3. Hydraulic properties of the North Atlantic Coastal Plain Aquifer System (*87*)**.**

**Hydrogeologic unit***	**Hydraulic conductivity (ft/d) (median)**	**Vertical hydraulic conductivity (ft/d) (median)**	**Specific storage (1/ft) (median)**
Surficial	442.83	2.25	0.000001
Upper Chesapeake confining unit	0.0001273	0.0002747	0.00009522
Upper Chesapeake aquifer	175.92	11.36	0.000001094
Lower Chesapeake confining unit	0.1131	0.08752	0.0001083

Hydrogeologic unit*	Hydraulic conductivity (ft/d) (median)	Vertical hydraulic conductivity (ft/d) (median)	Specific storage (1/ft) (median)
Lower Chesapeake aquifer	139.2	9.4	0.0000009957
Calvert confining unit	0.008339	0.0004715	0.0001006
Piney Point aquifer	20.87	11.64	0.000001148
Nanjemoy-Marlboro confining unit	0.001574	0.0001442	0.00007574
Aquia aquifer	24.4	5.58	0.00000114
Monmouth-Mount Laurel confining unit	0.000002699	0.000001851	0.00001076
Monmouth-Mount Laurel aquifer	32.08	0.48	0.0000009637
Matawan confining unit	0.00006876	0.00003257	0.0000119
Matawan aquifer	51.29	6.25	0.0000007723
Magothy confining unit	0.000001075	0.0000005416	0.00001023
Magothy aquifer	60	7.48	0.0000009878
Potomac confining unit	0.00000131	0.000002127	0.000009153
Potomac-Patapsco aquifer	20.57	0.29	0.000001064
Potomac-Patuxent confining unit	0.000002174	0.000001678	0.000007973
Potomac-Patuxent aquifer	57.26	5.8	0.0000007951

*Hydrogeologic units reported here are from(*75*). These are the same units used in our regional aquifer analysis.

(d) Floridan Aquifer System

The Floridan Aquifer System encompasses ~259,000 square kilometers across Florida, parts of Georgia, Alabama, and South Carolina. The Floridan aquifer system is a complex carbonate system that typically behaves as one aquifer system over much of its extent, with clastic updip areas in Alabama, Georgia, and South Carolina. The sediments throughout the system are Paleocene to Miocene in age, and generally thicken seaward. The aquifer system is primarily divided into the Upper Floridan and Lower Floridan aquifer units, numerous confining units, and several high-permeability zones exist within the Upper and Lower Floridan formations (*99*). Detailed hydrogeologic units in the Floridan Aquifer System include the surficial unit (called the Biscayne aquifer in southern Florida), the upper confining unit, the Upper Floridan aquifer, the Ocala-Avon Park lower-permeability zone (semi-confining), the Avon Park permeable zone, the Lisbon-Avon Park composite unit (semi-confining), the middle Avon Park composite unit (semi-confining), the Lower Floridan aquifer, the Lower Avon Park permeable zone, the Glauconite marker unit (semi-confining), and the Oldsmar permeable zone. These units are not continuous throughout the Floridan Aquifer System. Overall, the Floridan is characterized primarily by carbonate rocks that have varying degrees of vertical and horizontal interconnection. Where carbonates of the Floridan crop out, karstic features are evident due to dissolution. In the subsurface, collapse sinkholes have been mapped, including collapse features at depth and within confined parts of the system (*100*). The surficial aquifer unit is only productive in two areas, and otherwise acts as temporary storage for groundwater that recharges the deeper aquifer units.

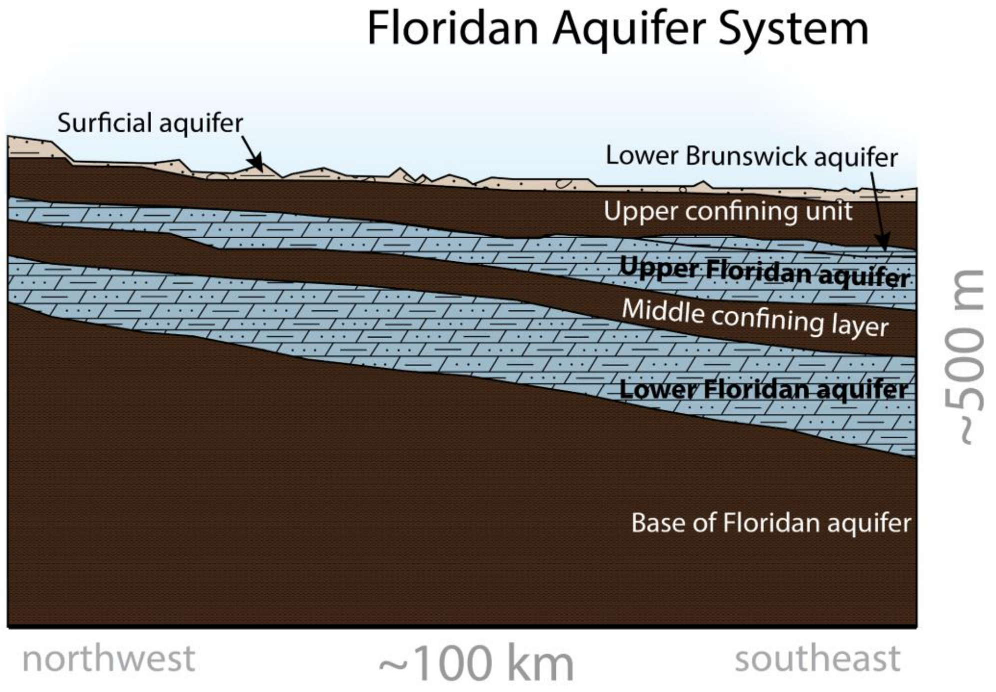

Fig. S4. Cross section of the Floridan Aquifer System. The hydrogeologic cross section is based on Fig. 21 by (*88*); we acknowledge M. GebreEgziabher for their help digitizing the hydrogeologic cross section. Carbonate rock aquifers (blue shaded formations) dip southeast towards the sea (this cross section depicts a location in eastern Georgia). These carbonate rock aquifers (i.e., the Upper Floridan and Lower Floridan aquifers) are separated by a confining unit.

Table S4. Hydraulic properties of the Floridan Aquifer System (*101*)**.**

Hydrogeologic unit*	Hydraulic conductivity (ft/d) (median)	Vertical hydraulic conductivity (ft/d) (median)	Storage coefficient (dimensionless) (median)
Upper Floridan			
unconfined			0.0009
thinly confined			0.0006
confined			0.0004
Middle Floridan			0.0002
Lower Floridan			0.0004

*Hydrogeologic units reported here are from (*101*). In this report, the Upper Floridan was separated into unconfined, thinly confined, and confined areas. We recognize that these hydrogeologic units do not straightforwardly correlate (in all cases) to the hydrogeologic units used in our analysis. We use the hydrogeologic units reported in digital surfaces of the Floridan Aquifer System created by (*76*).

(e) Mississippi Embayment Regional Aquifer System

The Mississippi Embayment Regional Aquifer System spans ~202,000 square kilometers across eight states: Alabama, Arkansas, Illinois, Kentucky, Louisiana, Mississippi, Missouri, and Tennessee. The embayment is considered to be part of the Gulf Coastal Plain and is a syncline that plunges to the south whose axis generally parallels the Mississippi River (*102*). This southwestward plunging trough contains Cretaceous and Cenozoic sediments. There are several fault zones within the Mississippi Embayment (including the New Madrid, Arkansas, and Pickens-Gilbertown fault zones), as well as three structural highs (Sabine uplift, Monroe uplift, and Jackson dome). The axis of the Mississippi Embayment is influenced by these structural features (*103*). Primary hydrogeologic units include the Mississippi River Valley Alluvial aquifer, the Vicksburg-Jackson confining unit, the Upper Claiborne aquifer, the Middle Claiborne confining unit, the Middle Claiborne aquifer, the Lower Claiborne confining unit, the Lower Claiborne aquifer, the Middle Wilcox aquifer, the Lower Wilcox aquifer, and the Midway confining unit. Recharge to the system prior to development is understood to derive from precipitation and streamflow losses (*103*).

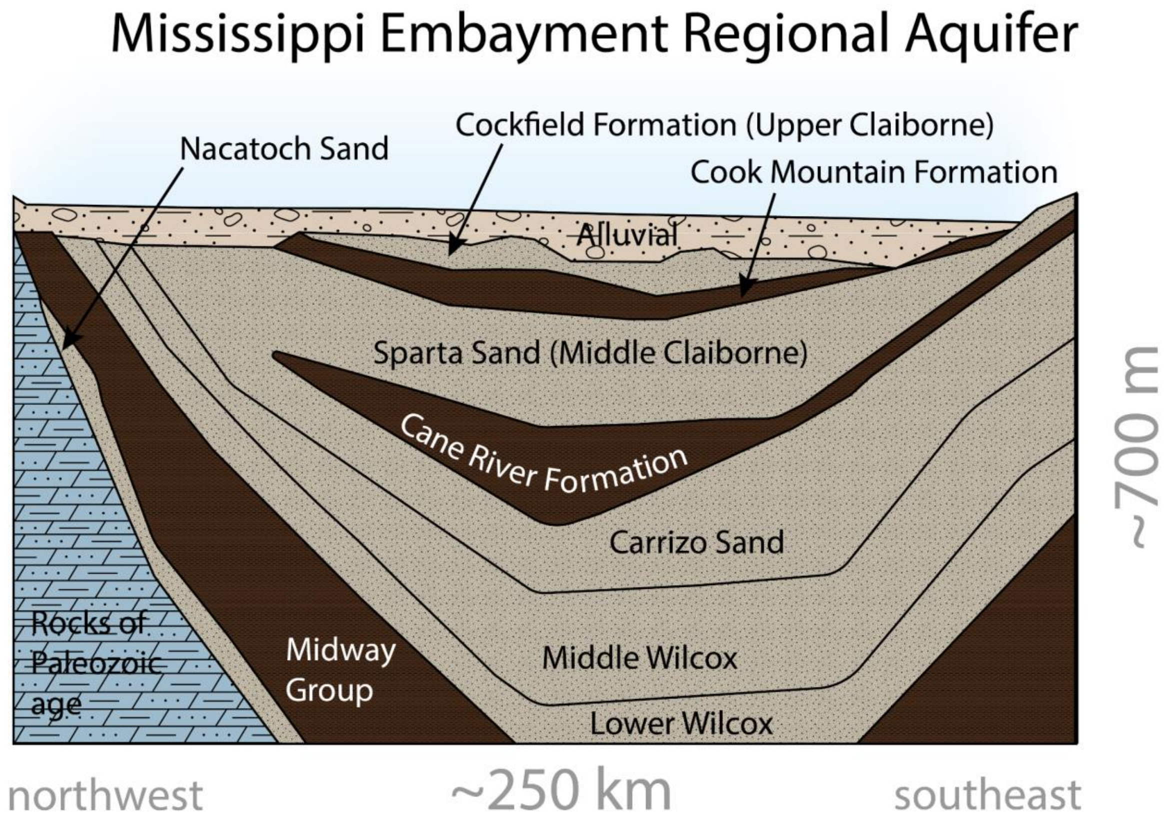

Fig. S5. Cross section of the Mississippi Embayment Regional Aquifer System. The hydrogeologic cross section is based on Fig. 69 by (*89*); we acknowledge M. GebreEgziabher for their help digitizing the hydrogeologic cross section. Sedimentary aquifers (light tan shades) are layered and, in places, separated by confining units (e.g., the Cane River Formation).

Table S5. Hydraulic properties of the Mississippi Embayment Regional Aquifer System. These values are final calibrated hydraulic parameters from the Mississippi Embayment Regional Aquifer Study (MERAS) by (*103*)**.**

**Hydrogeologic unit***	**Hydraulic conductivity (ft/d) (median)**	**Hydrogeologic unit**	**Specific storage (1/ft) (median)**
Vicksburg-Jackson confining unit	1	Vicksburg-Jackson confining unit	0.000000346
Undifferentiated Claiborne group	48.7	Undifferentiated Claiborne group	0.000000368
Upper Claiborne aquifer	26.3	Upper Claiborne aquifer	0.000000259
Middle Claiborne confining unit	0.154	Middle Claiborne confining unit	0.000000288
Middle Claiborne confining unit zone 50	146.1	Middle Claiborne confining unit zone 50	0.00000851
Middle Claiborne confining unit zone 51	34.8	Middle Claiborne confining unit zone 51 and 53*	0.00000103
Middle Claiborne confining unit zone 52	27.2	Middle Claiborne confining unit zone 52*	0.000000992
Middle Claiborne confining unit zone 61	3.7		
Middle Claiborne confining unit zone 53	10.2		
El Dorado confining unit	0.00453	El Dorado confining unit	0.000000365
El Dorado sand	59	El Dorado sand	0.00000244
Lower Claiborne confining unit	0.0883	Lower Claiborne confining unit	0.000000308
Winona-Tallahatta aquifer	26.9	Winona-Tallahatta aquifer	0.000000281
Lower Claiborne aquifer	25	Lower Claiborne aquifer	0.000000279
Wilcox aquifer in zone 110	5.6	Wilcox aquifer in zone 110	0.000000365
Middle Wilcox aquifer	2.4	Middle Wilcox aquifer	0.000000336
Lower Wilcox aquifer	24.6	Lower Wilcox aquifer	0.000000339

*Hydrogeologic units reported here are from (*103*). These units are based on the MERAS model created by (*77*) (Mississippi River Valley aquifer, Vicksburg-Jackson group confining unit, Upper Claiborne aquifer, Middle Claiborne confining unit, Middle Claiborne aquifer, Lower Claiborne confining unit, Lower Claiborne aquifer, Middle Wilcox aquifer, Lower Wilcox aquifer, Midway confining unit) but include the addition of the El Dorado confining unit, El Dorado Sand, and the Winona-Tallahatta aquifer, which are minor units but used extensively in certain local areas. These units represent layers in the hydrological model (MERAS), which sometimes are grouped in zones of similar hydraulic properties, as well as multiple zones within a single aquifer unit. Please refer to (*103*) for a detailed description of these units and zones. We recognize that these hydrogeologic units do not straightforwardly correlate (in all cases) to the hydrogeologic units used in our analysis. We use the hydrogeologic units reported in the MERAS model of (*77*).

(f) Houston-Galveston Area

The Houston-Galveston area of the Gulf Coast Aquifer System covers 65,000 square kilometers along the Gulf Coast of Texas. The system outcrops in the northwestern area (outcrops run parallel to the coast) and downdips toward the Gulf of Mexico. Overall, the system consists of alternating continental and marine sediments, primarily sands and clays. Sediment ages range from the Miocene to Holocene. Some salt domes exist in the study area (*104*). The primary hydrogeologic units are the Chicot aquifer, the Evangeline aquifer, the Burkeville confining unit, the Jasper aquifer, and the Catahoula confining unit. The uppermost parts of the aquifer system are considered to be unconfined, but as depth increases and interbedded sand and clays accumulate, conditions become confined. Generally, groundwater recharges at topographical highs of aquifer outcrops in the northwestern part of the system; recharge to shallow zones follows relatively short flow paths and discharges to streams. Recharge that infiltrates to intermediate and deeper zones of the system would travel southeastward under predevelopment conditions, discharging in topographically low areas of the coast (*104*). s

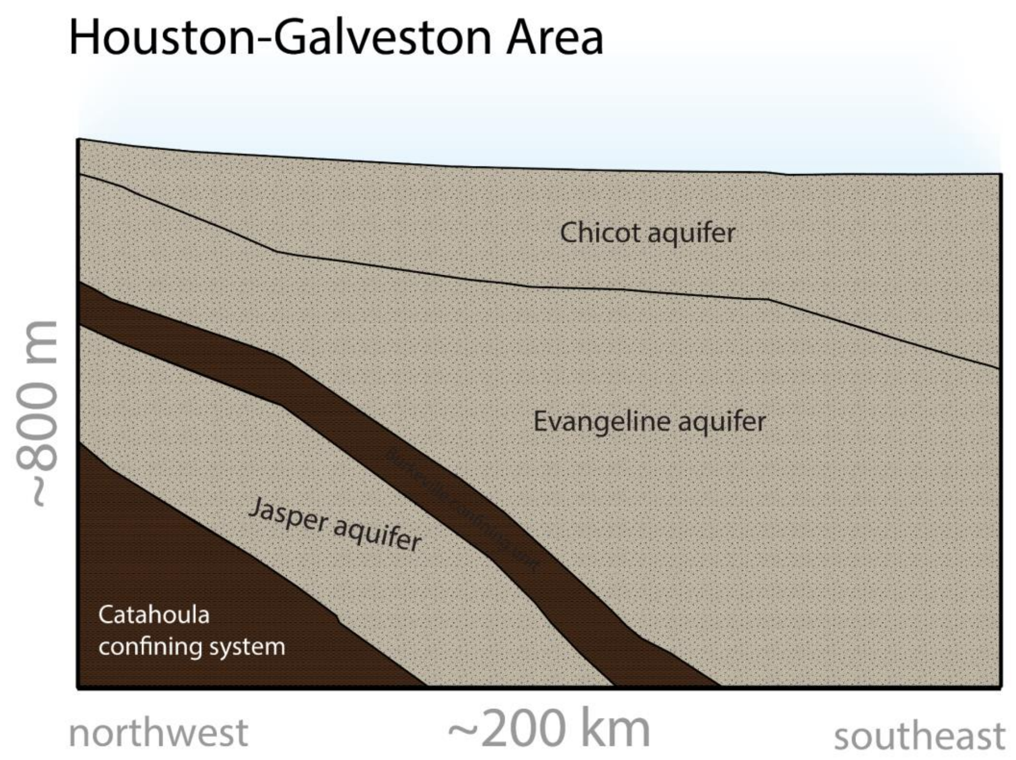

Fig. S6. Cross section of the Houston-Galveston Area of the Gulf Coast Aquifer System. The hydrogeologic cross section was based on Fig. 2 of (*90*); we acknowledge M.

GebreEgziabher for their help digitizing the hydrogeologic cross section. The aquifer system is characterized by layered sedimentary formations, some being poorly consolidated. The uppermost formations (Chicot and Evangeline) are depicted here as aquifers but are characterized by interspersed layers of coarser and finer grained sediments, meaning groundwater stored in deeper portions of these formations can exist under semi-confined or confined conditions.

Table S6. Hydraulic properties of the Houston-Gulf Coast Aquifer System. Properties are calibrated-parameter values from the Houston Area Groundwater Model (HAGM)(*78*)**.**

**Hydrogeologic unit***	**Hydraulic conductivity (ft^2/d) (min)**	**Hydraulic conductivity (ft^2/d) (max)**	**Storativity (dimensionless) (min)**	**Storativity (dimensionless) (max)**
Chicot aquifer	0.004	39.9	0.002	0.156
Evangeline aquifer	0.39	30.8	0.001	0.182
Burkeville confining unit	0.000009	0.021	0.00001	0.05
Jasper aquifer	0.864	21.23	0.0000041	0.201

*Hydrogeologic units reported here are from (*78*) in the HAGM model. Hydraulic properties are estimated within the model only for the Chicot aquifer, Evangeline aquifer, Burkeville confining unit, and Jasper aquifer. The hydrogeologic units we used in our analysis were also from the HAGM model, but include the Chicot aquifer, Evangeline aquifer, Burkeville confining unit, Jasper aquifer, and the Catahoula confining unit.

(g) Roswell Artesian Basin

The Roswell Artesian Basin covers ~10,000 square kilometers of the lower Pecos Valley in southeastern New Mexico. The aquifer system consists of an eastward-dipping carbonate aquifer overlain by a leaky confining unit, all overlain by an unconfined alluvial aquifer (*24*). The basin is typically characterized as a two-aquifer system: the carbonate aquifer (often called the "artesian" aquifer, or Artesia) and the alluvial aquifer (often called the "shallow" aquifer). The carbonate aquifer is underlain by the Yeso formation, a lower confining unit. The carbonate aquifer becomes confined about 10 kilometers west of the city of Roswell, and the eastern boundary of the basin is a no-flow boundary along the Pecos River (*24*). Carbonate rocks consist primarily of limestones that contain highly porous, transmissive water bearing zones with secondary porosity formed by subsurface dissolution of evaporites. In predevelopment times, groundwater flowed towards the southeast, downgradient from the recharge area in the Sacramento Mountains; the carbonate aquifer was directly recharged at its outcrop, as well as through sinkholes and fractures associated with the Pecos Buckles (wrench fault zones).

Groundwater would then flow upward through leaky confining areas into the alluvial aquifer and finally to the Pecos River (*24*).

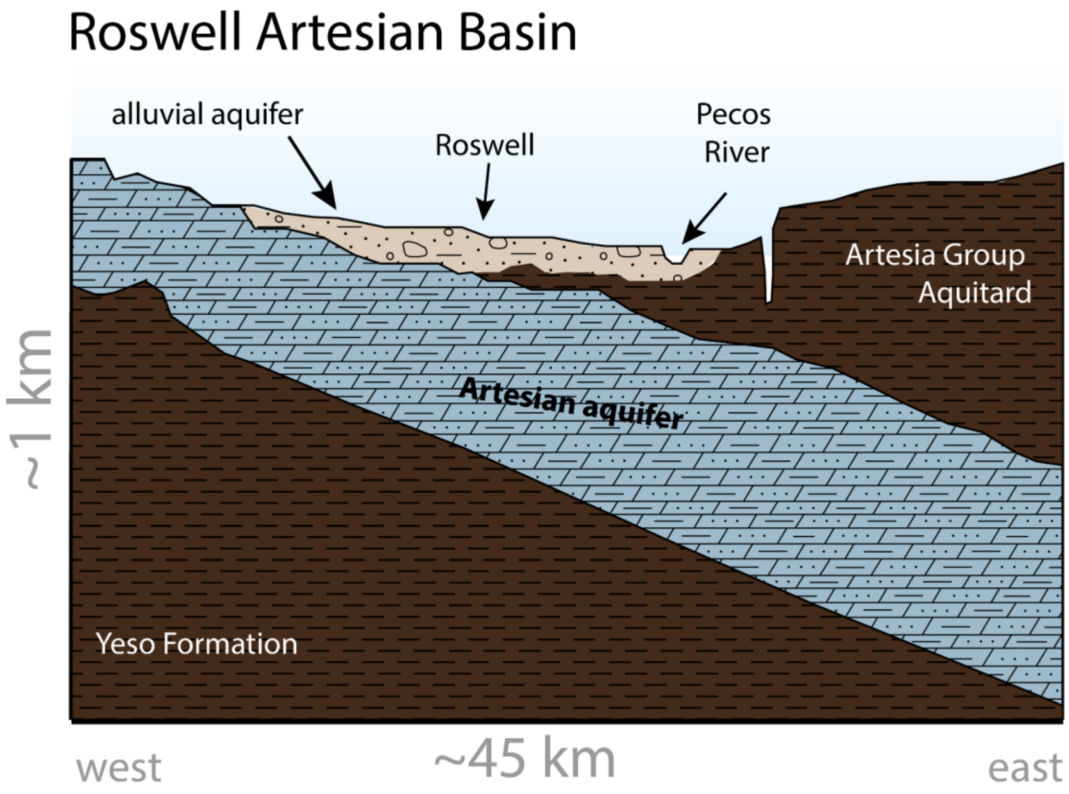

Fig. S7. Cross section of the Roswell Artesian Basin. The hydrogeologic cross section was based on Fig. 2 of (*24*). The "Artesian Aquifer" outcrops in the west and becomes a confined aquifer in the eastern portion of this aquifer system. It is a carbonate rock aquifer (see light blue shading in cross section).

We did not identify data describing the hydraulic properties (such as storativity) of the Roswell Artesian Basin in our literature review.

(h) Central Valley

The Central Valley covers 52,000 square kilometers in California. The Central Valley is an intermontane structural trough bounded on the west by the Coast Ranges and on the east by the Sierra Nevada. The Central Valley is divided into two subregions, the Sacramento Valley (northern one-third) and the San Joaquin Valley (southern two-thirds). Sediments in the valley are unconsolidated to semi-consolidated gravel, sand, silt, and clay, and range from Jurassic to Holocene in age. Most of the groundwater is contained in the upper part of the sediments which is composed of post-Eocene continental deposits (*80*). In the western part of the San Joaquin Valley, a confining unit called the Corcoran Clay Member of the Tulare Formation exists. Numerous other clay and silt lenses are present throughout the valley and are thought to comprise up to 50 percent of the total sediments in the system (*105*). Because of the high percentage of clays and fine-grained materials, it is generally considered that the aquifer becomes confined several hundred feet below the surface (*105*). There are no strictly defined aquifer units in the Central Valley, rather, the aquifer is designated into unconfined and confined zones, and further classified by sediment textures. In the U.S. Geological Survey Central Valley Hydrologic Model, the Central Valley is divided into ten vertical layers and characterized on the basis of sediment textures (*80*). The Central Valley is a highly human-modified hydrological system. Generally, pre-development recharge occurred directly from precipitation in the valley and snowmelt in the mountains, with groundwater discharge to streams and springs.

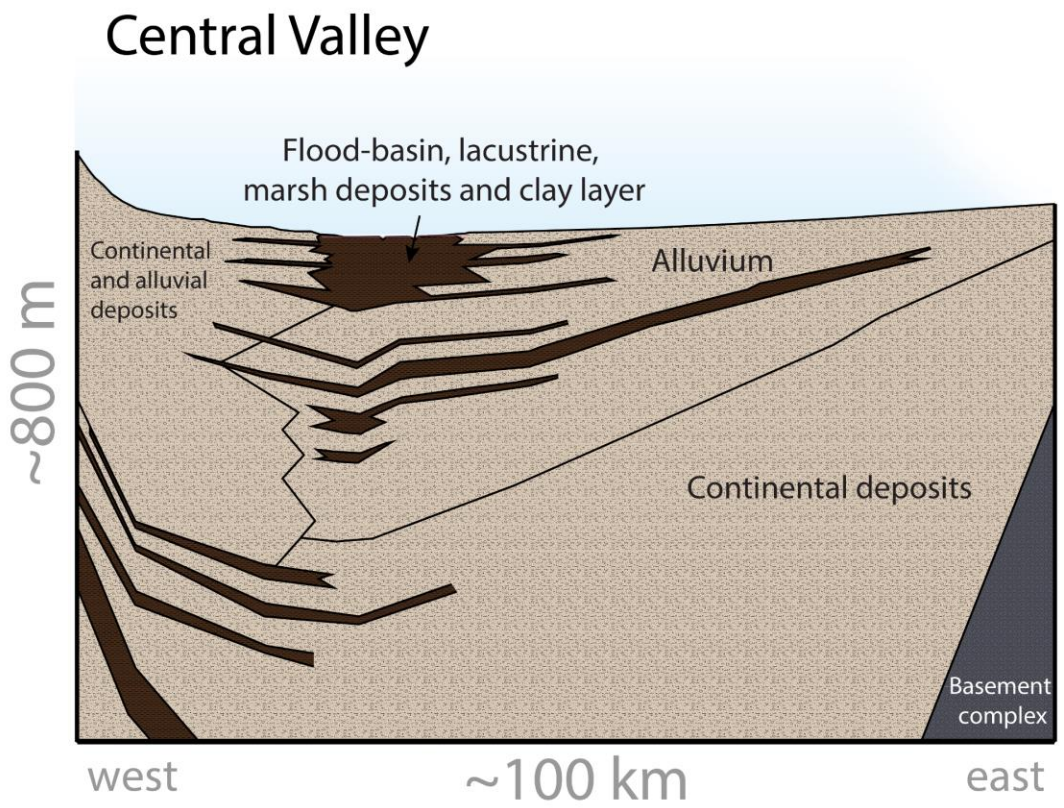

Fig. S8. Cross section of the Tulare Basin of the Central Valley. The hydrogeologic cross section was based on Plate 1 of (*91*); we acknowledge M. GebreEgziabher for their help digitizing the hydrogeologic cross section. The aquifer system is characterized by heterogenous sediments, some being poorly consolidated. Finer grained layers (e.g., the Corcoran clay) act as local- or regional-scale aquitards, meaning groundwater stored at deeper depths is characterized by semi-confined or confined conditions.

Table S7. Hydraulic properties of the Central Valley, by subregion. Hydraulic conductivity for coarse and fine grained materials are from the Central Valley Hydrologic Model (*80*)**. Specific storage for the San Joaquin Valley is reported from** (*106*)**.**

**Subregion**	**Hydraulic conductivity (coarse grained materials) (ft/d)**	**Hydraulic conductivity (fine grained materials) (ft/d)**	**Specific storage (mean) (1/ft)**
San Joaquin Valley	3300	0.24	0.000000086
Sacramento Valley	670	0.075	

S2. Groundwater withdrawals in our regional aquifer systems

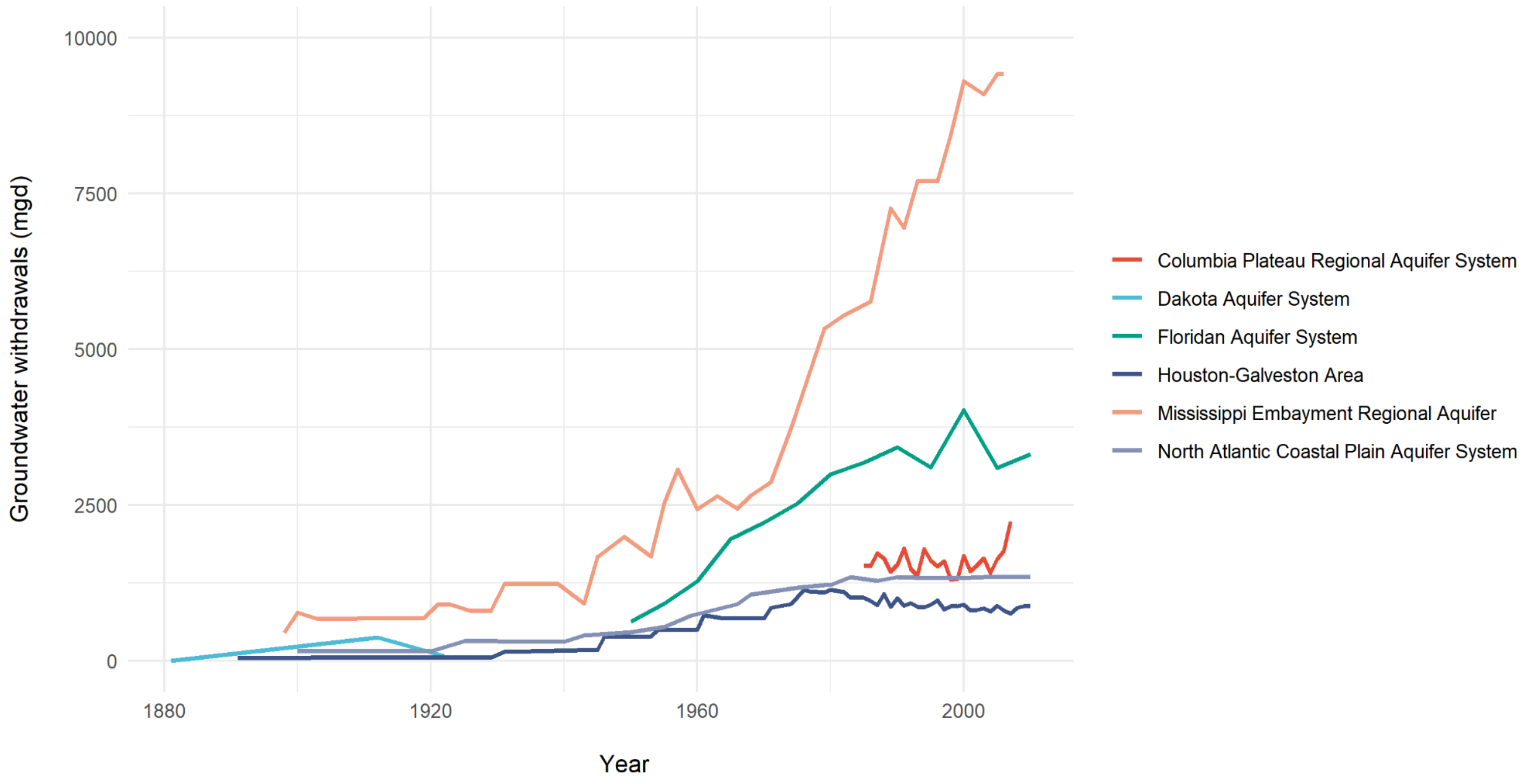

Fig. S9. Groundwater withdrawals for six of the eight regional aquifer systems over time. Groundwater withdrawal data were available for the years 1985-2007 in the Columbia Plateau Regional Aquifer System **(*85*)**, 1881-1922 in the Dakota Aquifer System **(*86*)**, 1900-2010 in the North Atlantic Coastal Plain Aquifer System **(*107*)**, 1950-2010 in the Floridan Aquifer System **(*100*)**, 1880-2007 in the Mississippi Embayment Regional Aquifer **(*108*)**, and 1891-2009 in the Houston-Galveston Area **(*78*)**. Groundwater withdrawal data were not available for the Roswell Artesian Basin or the Central Valley. Groundwater withdrawals are reported in millions of gallons per day (mgd on y-axis), where 1 mgd equates to 3785.4 m3/day

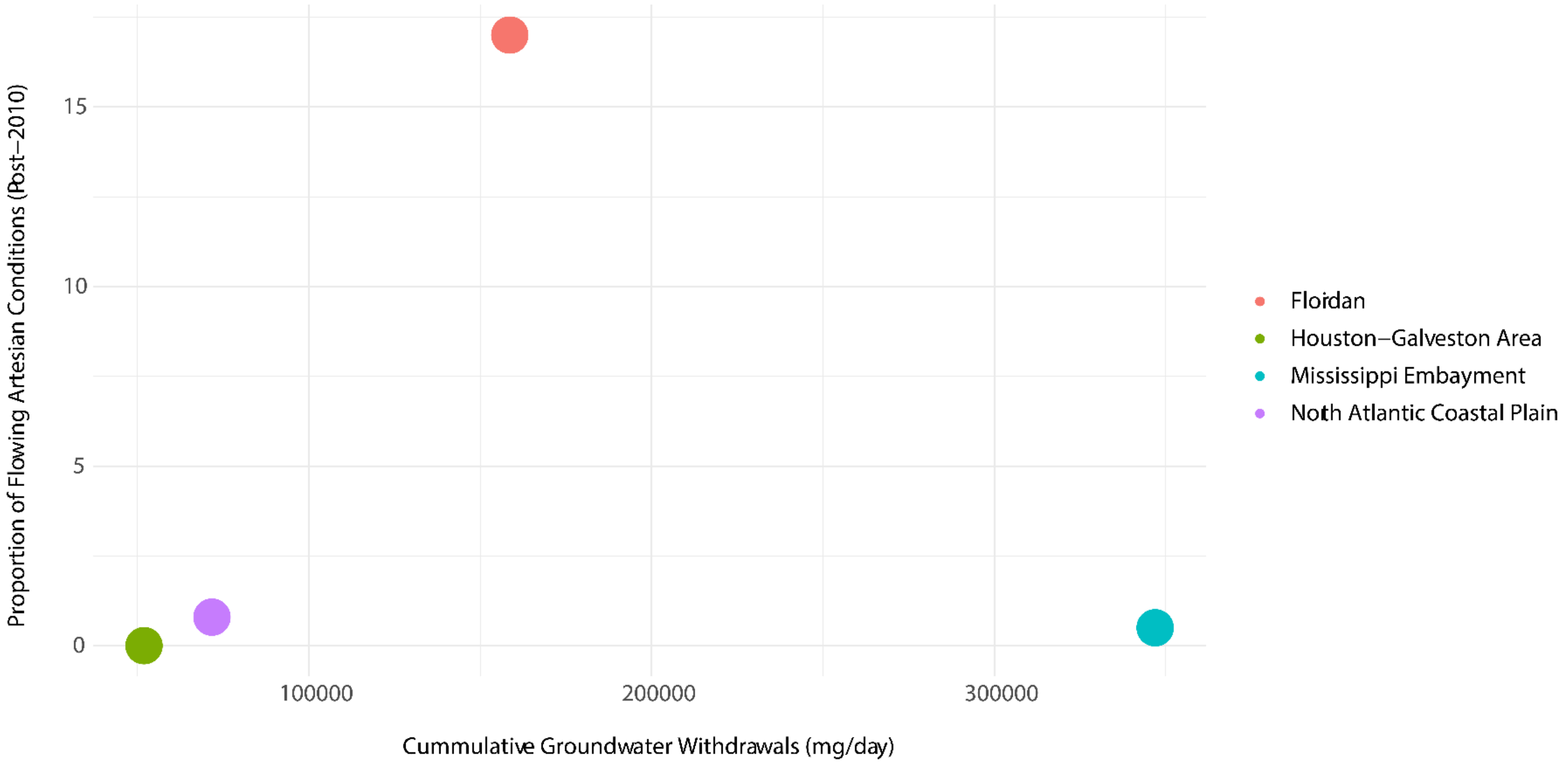

Fig. S10. Cumulative groundwater withdrawals (mg/day) and the proportion of wells exhibiting flowing artesian conditions in our post-2010 dataset. Cumulative withdrawals were calculated for the years 1900-2007 where data were available (four of the eight regional aquifer systems: the Floridan Aquifer System (*100*), Houston-Galveston Area (*78*), Mississippi Embayment Regional Aquifer (*108*), and the North Atlantic Coastal Plain Aquifer System (*107*)). The proportion of wells exhibiting flowing artesian conditions on the y-axis are reported as a percentage (consistent with our results in the main text).

Table S8. Cumulative groundwater withdrawals (mg/day) from 1900-2007 (*78, 100, 107, 108*)**. The proportion of wells exhibiting flowing artesian conditions in pre-1910 and post-2010 are reported (as a percentage).**

**Aquifer System**	**Groundwater Withdrawals (mg/day)**	**Proportion of wells exhibiting flowing artesian conditions Pre-1910**	**Proportion of wells exhibiting flowing artesian conditions Post-2010**
North Atlantic Coastal Plain	71775	83	0.8
Mississippi Embayment	346926	48	0.5
Houston-Galveston Area	51882	96	0
Floridan	158558	58	17

S3. 3D Hydrostratigraphic data of regional aquifer systems

We compiled hydrostratigraphic data for the following eight regional aquifer systems: (a) Columbia Plateau Regional Aquifer System, (b) Dakota Aquifer System, (c) North Atlantic Coastal Plain Aquifer System, (d) Floridan Aquifer System, (e) Mississippi Embayment Regional Aquifer, (f) Houston-Gulf Coast Aquifer System, (g) Roswell Artesian Basin, and (h) Central Valley Aquifer.

(a) Columbia Plateau Regional Aquifer System

Data source: United States Geological Survey
Projection: Lambert Conformal Conic
Datum: North American Datum of 1983
Type: Raster
Year Published: 2011

Date downloaded: 2/16/2022
URL: https://pubs.usgs.gov/sir/2010/5246/

Webpage title: Three-Dimensional Model of the Geologic Framework for the Columbia Plateau Regional Aquifer System, Idaho, Oregon, and Washington

Metadata page: https://pubs.usgs.gov/sir/2010/5246/

Webpage title: Three-Dimensional Model of the Geologic Framework for the Columbia Plateau Regional Aquifer System, Idaho, Oregon, and Washington

Data citation (*74*).

(b) Dakota Aquifer

Data source: Compiled from South Dakota Department of Natural Resources Lithologic Logs Database
Projection: Albers Conical Equal Area
Datum: WGS 1984
Type: Raster

Date downloaded: 11/27/2021
URL: http://cf.sddenr.net/lithdb/

Webpage title: Lithologic Logs Database

Steps for Dakota formation raster creation:

(1) We identified unique URLs that provide lithological information from the South Dakota Lithological Logs Database (*87*) (n=35,000 records)
(2) We utilised program Octoparse to crawl each of 35,000 URLs and compile web page information in a tabular format
(3) We searched through 35,000 lithological log records for string "dak" to indicate Dakota formation (n=293 logs)
(4) Each lithological log record (n=293) was manually examined for top and bottom of Dakota formation, top and bottom of Dakota recorded
(5) We plotted latitude and longitude coordinates for lithological logs with a top and bottom for the Dakota formation in ArcPro
(6) We used Natural Neighbour Inverse Distance Weighted to create a raster layer of the top and bottom of the Dakota formation
(7) South Dakota Monitoring Wells (*79*) were used to check the accuracy of the raster layers we created for the top and bottom of the Dakota formation. For wells identified as tapping the Dakota formation (n=43), n=26 fell into the top and bottom of our rasters of the Dakota formation, n=15 fell under the top of the Dakota formation only (not contained within our boundaries of the bottom of the Dakota formation), and n=2 wells did not fall within our raster boundaries
(8) Our analysis seeks to identify confined wells, therefore we decided to utilise the top of the Dakota formation for our analysis of the Dakota Aquifer System. Any well bottom that taps beneath the top of the Dakota formation raster was included in the analysis as a confined well

(c) North Atlantic Coastal Plain Aquifer System

Data source: United States Geological Survey
Projection: NAD 1983 Albers
Datum: North American Datum of 1983
Type: Raster
Year Published: 2016

Date downloaded: 06/01/2020
URL: https://www.sciencebase.gov/catalog/item/57df93b2e4b090825000fb55
Webpage title: Digital elevations and extents of regional hydrogeologic units in the Northern Atlantic Coastal Plain aquifer system

Metadata page: https://www.sciencebase.gov/catalog/item/57df93b2e4b090825000fb55
Webpage title: Digital elevations and extents of regional hydrogeologic units in the Northern Atlantic Coastal Plain aquifer system

Data Citation (*75*).

(d) Floridan Aquifer System

Data source: United States Geological Survey
Projection: NAD 1983 Albers
Datum: North American Datum of 1983
Type: Raster
Year Published: 2015

Date downloaded: 07/08/2020
URL: https://pubs.usgs.gov/ds/0926/

Webpage title: Digital Surfaces and Thicknesses of Selected Hydrogeologic Units of the Floridan Aquifer System in Florida and Parts of Georgia, Alabama, and South Carolina

Metadata page: https://pubs.usgs.gov/ds/0926/
Webpage title: Digital Surfaces and Thicknesses of Selected Hydrogeologic Units of the Floridan Aquifer System in Florida and Parts of Georgia, Alabama, and South Carolina

Data Citation (*76*).

(e) Mississippi Embayment Regional Aquifer

Data source: United States Geological Survey
Projection: USA Contiguous Albers Equal Area Conic USGS Version
Datum: North American Datum of 1983
Type: Raster
Year Published: 2008

Date downloaded: 02/11/2020
URL: https://pubs.usgs.gov/sir/2008/5098/downloads/WRD_NSDI_Node.html
Webpage title: Digital Surfaces and Thicknesses of Selected Hydrogeologic Units within the Mississippi Embayment Regional Study Aquifer (MERAS)
Model: Hydrologic Model Layer Surfaces

Metadata page: https://pubs.usgs.gov/sir/2008/5098/
Webpage title: Digital Surfaces and Thicknesses of Selected Hydrogeologic Units within the Mississippi Embayment Regional Study Aquifer (MERAS)

Data Citation (*77*).

(f) Houston-Gulf Coast Aquifer System

Data source: Texas Water Development Board
Projection: Albers Conical Equal Area
Datum: North American Datum of 1983
Type: Raster
Year Published: 2013

Date downloaded: 1/12/2022
URL: https://www.twdb.texas.gov/groundwater/models/download.asp
Webpage title: Groundwater Availability Model (GAM) and Geodatabase Downloads
Model: Gulf Coast Aquifer (northern portion) GAM

Metadata page: https://www.twdb.texas.gov/groundwater/models/gam/glfc_n/glfc_n.asp
Webpage title: Northern portion of the Gulf Coast Aquifer System

Data citation (*78*).

(g) Roswell Artesian Basin

Data source: New Mexico Bureau of Geology and Mineral Resources

Projection: Transverse Mercator
Datum: North American Datum of 1983
Type: Raster
Year Published:2020

Date downloaded: 04/05/2022
URL: https://geoinfo.nmt.edu/publications/openfile/details.cfml?Volume=614

Webpage title: A Three-Dimensional Hydrogeologic Model from the Pecos Slope to the Southern High Plains, South Eastern New Mexico
Model: PecosSlope_HydrogeologicModel.mpk

Metadata page: https://geoinfo.nmt.edu/publications/openfile/details.cfml?Volume=614

Webpage title: A Three-Dimensional Hydrogeologic Model from the Pecos Slope to the Southern High Plains, Southeastern New Mexico

Data citation: (*79*).

(h) Central Valley Aquifer

Data source: United States Geological Survey
Projection: Albers Conical Equal Area
Datum: North American Datum of 1983
Type: Raster
Year Published: 2012

Date downloaded: 06/23/2020
URL: https://ca.water.usgs.gov/projects/central-valley/central-valley-hydrologic-model.html
Webpage title: California's Central Valley
Model: Central Valley Hydrologic Model

Metadata page: https://ca.water.usgs.gov/projects/central-valley/central-valley-hydrologic-model.html
Webpage title: California's Central Valley

Data Citation (*80*).

S4. Regional aquifer systems results pre-1910 to post-2010

In all of our regional aquifer systems (n=8), we examine the number of flowing artesian wells in each aquifer unit pre-1910 and post-2010. We compare the percentages of wells exhibiting flowing artesian conditions for both of these time periods. We also completed an analysis of the change in hydraulic head (i.e., from pre-1910 to post-2010) for aquifer units where there were a sufficient number (at least n=25) of wells. For the change in head analysis, we present figures of the change in head for the two time periods as well as median values of hydraulic head for the two time periods (pre-1910, post-2010). In our post-2010 dataset, water levels for non-flowing wells and well-head pressure estimates for flowing wells were used to calculate head. In our pre-1910 dataset, water levels for non-flowing wells were used; for wells that were flowing artesian, we took the land surface elevation as the minimum head. Therefore, we note that the median values for the pre-1910 time period represent merely the baseline of hydraulic heads of the aquifer units examined (i.e., because wells that exhibit flowing artesian conditions have a hydraulic head that is, by definition, above the land surface elevation at the well site); the actual hydraulic head would likely be higher.

Table S9. Summary of artesian conditions for each regional aquifer system.

Aquifer System	Pre-1910			Post-2010		
	Wells (n)	Flowing artesian wells (n)	Percentage of wells that are flowing artesian (%)	Wells (n)	Flowing artesian wells (n)	Percentage of wells that are flowing artesian (%)
Columbia Plateau Regional Aquifer System	87 wells	2 wells	2%	105 wells	1 well	1%
Dakota Aquifer System	175 wells	162 wells	93%	64 wells	6 wells	9%
North Atlantic Coastal Plain Aquifer System	137 wells	113 wells	83%	1,670 wells	14 wells	0.8%

Aquifer System	Pre-1910			Post-2010		
	Wells (n)	Flowing artesian wells (n)	Percentage of wells that are flowing artesian (%)	Wells (n)	Flowing artesian wells (n)	Percentage of wells that are flowing artesian (%)
Floridan Aquifer System	144 wells	83 wells	58%	1,024 wells	172 wells	17%
Mississippi Embayment Regional Aquifer	238 wells	115 wells	48%	542 wells	3 wells	0.5%
Houston-Galveston	61 wells	51 wells	96%	630 wells	0 wells	
Roswell Artesian Basin	248 wells	248 wells	100%	32 wells	0 wells	
Central Valley	310 wells	237 wells	77%	2571 wells	6 wells	0.2%

(a) Columbia Plateau Regional Aquifer System

The Columbia Plateau Regional Aquifer System (Fig. 3A) changed the least between our two time periods, but also had the smallest pre-1910 artesian conditions. Pre-1910 (n=357 wells), 24% of wells (n=87) were classified as tapping confined conditions, with most confined wells found in the Wanapum and Grande Ronde units. Only two wells were found to be artesian (2.3%), in the Wanapum unit. Post-2010 reflect similar conditions (n=483 wells) with 22% of wells (n=105) classified as tapping confined conditions and one well (0.95%) found to be flowing artesian, also in the Wanapum unit.

Table S10. Columbia Plateau Regional Aquifer System flowing artesian conditions by geologic unit.

**Geologic formation that the well bottom lies within**	**Pre-1910**			**Geologic formation that the well bottom lies within**	**Post-2010**		
	Wells (n)	Flowing artesian wells (n)	Percentage of wells that are flowing artesian (%)		Wells (n)	Flowing artesian wells (n)	Percentage of wells that are flowing artesian (%)
Overburden (surficial)	0 wells	0 wells		Overburden (surficial)	0 wells	0 wells	
Mabton Interbeds & Saddle Mountains Basalt	0 wells	0 wells		Mabton Interbeds & Saddle Mountains Basalt	0 wells	0 wells	
Vantage Interbeds & Wanapum Basalt	62 wells	2 wells	3%	Vantage Interbeds & Wanapum Basalt	59 wells	1 wells	2%
Grande Ronde Basalt	24 wells	0 wells	0%	Grande Ronde Basalt	43 wells	0 wells	0%
Older Bedrock	0 wells	0 wells		Older Bedrock	0 wells	0 wells	

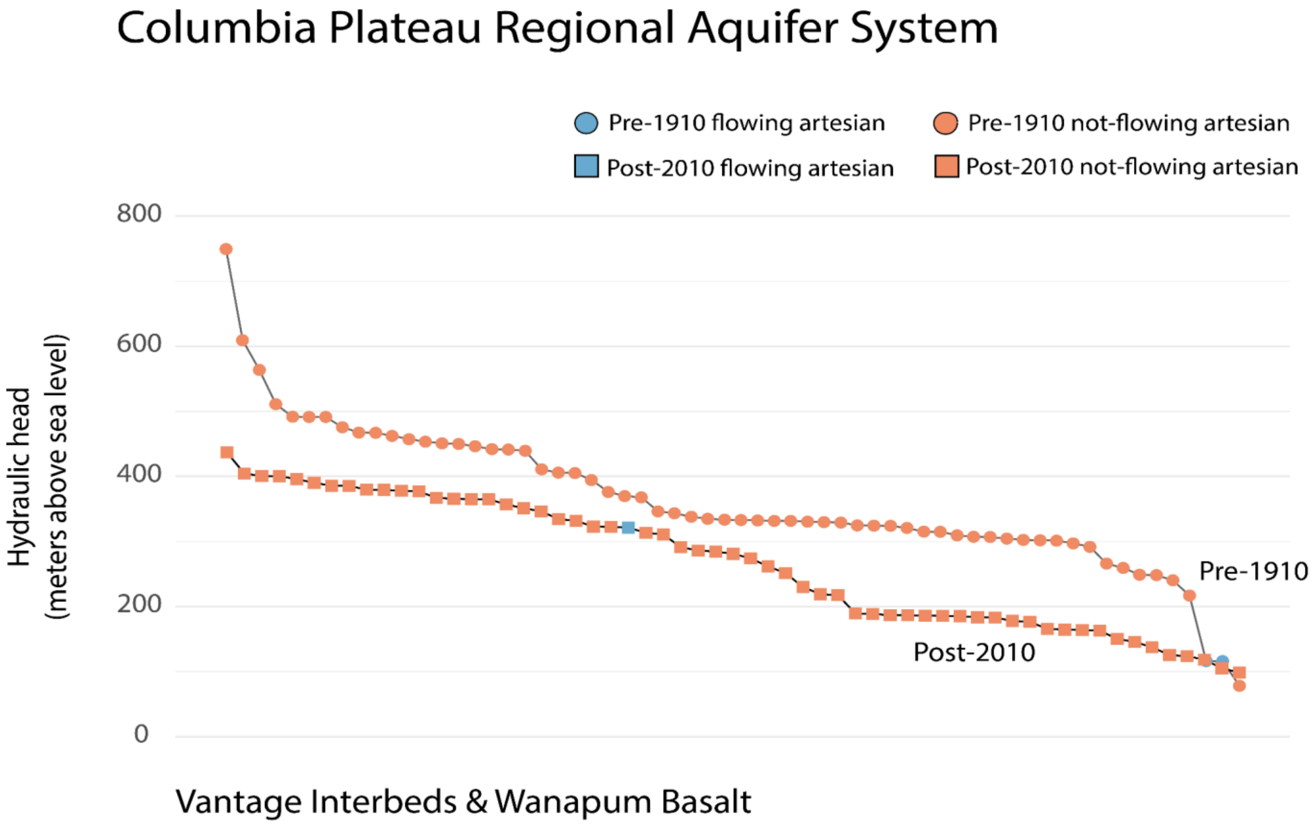

Fig. S11. Change of hydraulic head between our pre-1910 and post-2010 datasets in the Vantage Interbeds and Wanapum Basalt of the Columbia Plateau Regional Aquifer System. Each point represents one well water level measurement, and these points are ranked from highest (left) to lowest (right) hydraulic head along the x-axis of the plot. Circles represent the pre-1910 time period and squares represent the post-2010 time period. Blue shaded points represent flowing artesian wells while orange shaded points represent non-flowing wells. The Pre-1910 median hydraulic head was 333 meters above sea level; the post-2010 median was 281 meters above sea level. The pre-1910 median hydraulic head should be viewed as a minimum because we estimated the hydraulic head of flowing artesian wells to equate to the land surface elevation (but actual hydraulic heads for a flowing artesian well exceed the land surface elevation).

(b) Dakota Aquifer System

In the Dakota Aquifer System (Fig. 3B), some artesian conditions remain, but the reduction from pre-1910 to post-2010 in the prevalence of wells that exhibit flowing artesian conditions is substantial. All wells with bottoms that lie below the top of the Dakota Aquifer were classified as being confined (please see S1. (b) for details on our raster creation of the top of the Dakota unit). Specifically, in our pre-1910 dataset we find that 100% (n=175) of wells were classified as tapping confined conditions, with 92.6% of those wells tapping confined conditions (n=162) exhibiting flowing artesian conditions. In our post-2010, 100% (n=64) of wells were classified as tapping confined conditions with only 9.4% of confined wells (n=6) flowing artesian.

Table S11. Dakota Aquifer flowing artesian conditions by geologic unit.

**Geologic formation that the well bottom lies within**	**Pre-1910**			**Geologic formation that the well bottom lies within**	**Post-2010**		
	Wells (n)	Flowing artesian wells (n)	Percentage of wells that are flowing artesian (%)		Wells (n)	Flowing artesian wells (n)	Percentage of wells that are flowing artesian (%)
Dakota aquifer	175 wells	162 wells	93%	Dakota aquifer	64 wells	6 wells	9%

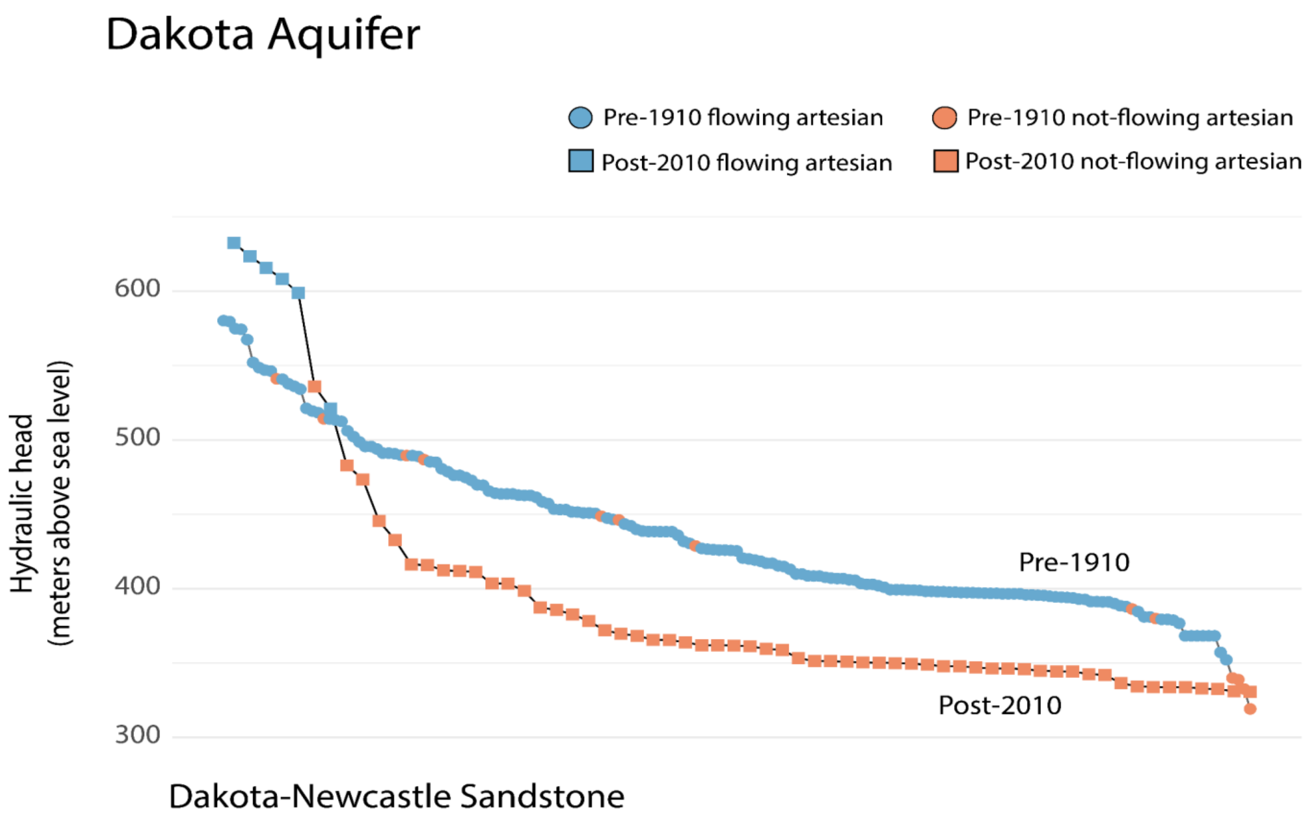

Fig. S12. Change of hydraulic head between our pre-1910 and post-2010 datasets in the Dakota-Newcastle Sandstone of the Dakota Aquifer System, South Dakota. Each point represents one well water level measurement, and these points are ranked from highest (left) to lowest (right) hydraulic head along the x-axis of the plot. Circles represent the pre-1910 time period and squares represent the post-2010 time period. Blue shaded points represent flowing artesian wells while orange shaded points represent non-flowing wells. The Pre-1910 median hydraulic head is 425 meters above sea level; the post-2010 median hydraulic head is 362 meters above sea level. The pre-1910 median hydraulic head should be viewed as a minimum because we estimated the hydraulic head of flowing artesian wells to equate to the land surface elevation (but actual hydraulic heads for a flowing artesian well exceed the land surface elevation).

(c) North Atlantic Coastal Plain Aquifer System

In the North Atlantic Coastal Plain Aquifer System (Fig. 3C), once-widespread flowing artesian conditions among wells (pre-1910) are now exceedingly rare in deep wells (post-2010. Specifically, in the North Atlantic Coastal Plain Aquifer System, we find that within our pre-1910 well water level dataset (n=2020 wells) 68% of wells (n=137) were classified as tapping confined conditions, with 82.5% of these confined wells (n=113) flowing artesian. Most flowing artesian wells were those with bottoms within the Lower Chesapeake aquifer and the Potomac-Patapsco aquifers. In our post-2010 well water level dataset (n=3603 wells), 46% of these wells (n=1670) were classified as tapping confined conditions, with only 0.84% of these confined wells (n=14) exhibiting flowing artesian conditions. The majority of these wells exhibiting flowing artesian conditions have bottoms within the Lower Chesapeake and Potomac-Patapsco aquifers.

Table S12. North Atlantic Coastal Plain Aquifer System by geologic unit.

	Pre-1910				Post-2010		
**Geologic formation that the well bottom lies within**	Wells (n)	Flowing artesian wells (n)	Percentage of wells that are flowing artesian (%)	**Geologic formation that the well bottom lies within**	Wells (n)	Flowing artesian wells (n)	Percentage of wells that are flowing artesian (%)
Surficial aquifer	0 wells	0 wells		Surficial aquifer	0 wells	0 wells	
Upper Chesapeake confining unit	0 wells	0 wells		Upper Chesapeake confining unit	0 wells	0 wells	
Upper Chesapeake aquifer	1 wells	0 wells	0%	Upper Chesapeake aquifer	141 wells	2 wells	1%
Lower Chesapeake confining unit	2 wells	2 wells	100%	Lower Chesapeake confining unit	15 wells	0 wells	0%
Lower Chesapeake aquifer	25 wells	24 wells	96%	Lower Chesapeake aquifer	94 wells	4 wells	4%
Calvert confining unit	10 wells	9 wells	90%	Calvert confining unit	14 wells	0 wells	0%

Geologic formation that the well bottom lies within	Pre-1910			Geologic formation that the well bottom lies within	Post-2010		
	Wells (n)	Flowing artesian wells (n)	Percentage of wells that are flowing artesian (%)		Wells (n)	Flowing artesian wells (n)	Percentage of wells that are flowing artesian (%)
Piney Point aquifer	7 wells	5 wells	71%	Piney Point aquifer	69 wells	2 wells	3%
Nanjemoy-Marlboro confining unit	11 wells	8 wells	73%	Nanjemoy-Marlboro confining unit	36 wells	0 wells	0%
Aquia aquifer	2 wells	2 wells	100%	Aquia aquifer	129 wells	1 wells	0.8%
Monmouth-Mount Laurel confining unit	2 wells	1 wells	50%	Monmouth-Mount Laurel confining unit	29 wells	0 wells	0%
Monmouth-Mount Laurel aquifer	5 wells	4 wells	80%	Monmouth-Mount Laurel aquifer	118 wells	1 wells	0.9%
Matawan confining unit	6 wells	5 wells	83%	Matawan confining unit	21 wells	0 wells	0%
Matawan aquifer	15 wells	14 wells	93%	Matawan aquifer	92 wells	1 wells	1%
Magothy confining unit	4 wells	4 wells	100%	Magothy confining unit	15 wells	0 wells	0%
Magothy aquifer	4 wells	2 wells	50%	Magothy aquifer	195 wells	0 wells	0%
Potomac confining unit	7 wells	6 wells	86%	Potomac confining unit	70 wells	1 wells	1%
Potomac-Patapsco aquifer	23 wells	20 wells	87%	Potomac-Patapsco aquifer	425 wells	2 wells	0.5%
Potomac-Patuxent confining unit	0 wells	0 wells		Potomac-Patuxent confining unit	44 wells	0 wells	0%
Potomac-Patuxent aquifer	5 wells	4 wells	80%	Potomac-Patuxent aquifer	119 wells	0 wells	0%
Basement	8 wells	3 wells	38%	Basement	44 wells	0 wells	0%

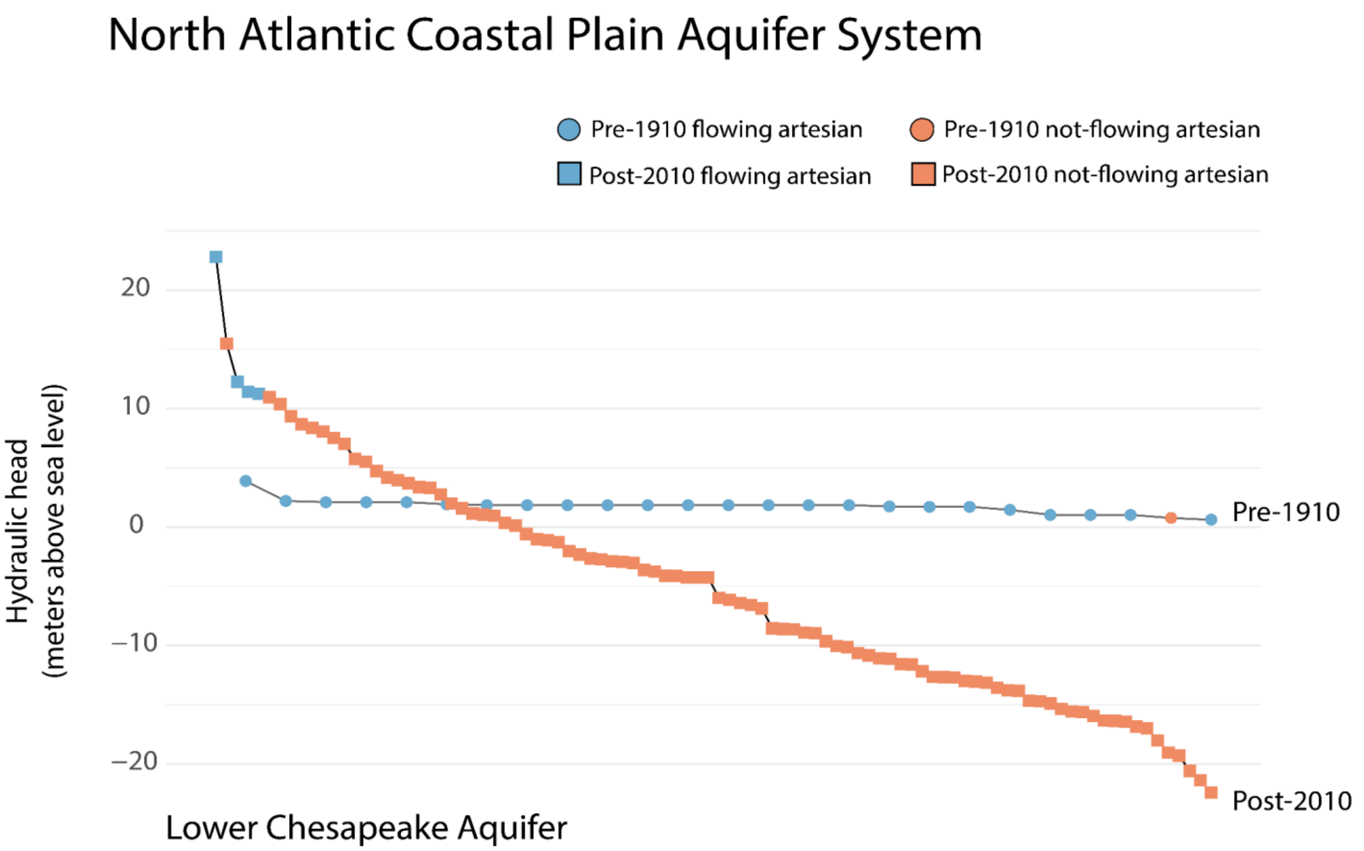

Fig. S13. Change of hydraulic head between our pre-1910 and post-2010 datasets in the Lower Chesapeake aquifer of the North Atlantic Coastal Plain Aquifer System. Each point represents one well water level measurement, and these points are ranked from highest (left) to lowest (right) hydraulic head along the x-axis of the plot. Circles represent the pre-1910 time period and squares represent the post-2010 time period. Blue shaded points represent flowing artesian wells while orange shaded points represent non-flowing wells. The pre-1910 median hydraulic head is 2 meters above sea level; the post-2010 median hydraulic head is -5 meters below sea level. The pre-1910 median hydraulic head should be viewed as a minimum because we estimated the hydraulic head of flowing artesian wells to equate to the land surface elevation (but actual hydraulic heads for a flowing artesian well exceed the land surface elevation).

(d) Floridan Aquifer System

Compared to most of the other eight regional aquifer systems that we studied here, the Floridan Aquifer System (Fig. 3D) exhibited a less substantial, yet still considerable, reduction in artesian conditions between our pre-1910 and our post-2010 datasets. Specifically, in our pre-1910 well water level dataset (n=249 wells) we classified 58% of wells (n=144) as tapping confined conditions, with 57.6% of these wells tapping confined conditions (n=83) categorized as flowing artesian. The number of wells exhibiting flowing artesian conditions are most numerous for wells with bottoms within the Upper Floridan aquifer and the Lower Floridan aquifer. Post-2010 (n=2201 wells), 47% of wells (n=1024) were classified as tapping confined conditions, with 16.8% of confined wells (n=172) flowing artesian. The majority of artesian wells were found to tap the Upper Floridan aquifer (n=126), while the number of artesian wells in the Lower Floridan aquifer decreased dramatically (n=1).

Table S13. Floridan Aquifer System flowing artesian conditions by geologic unit.

Geologic formation that the well bottom lies within	Pre-1910			Geologic formation that the well bottom lies within	Post-2010		
	Wells (n)	Flowing artesian wells (n)	Percentage of wells that are flowing artesian (%)		Wells (n)	Flowing artesian wells (n)	Percentage of wells that are flowing artesian (%)
Surficial aquifer	0 wells	0 wells		Surficial aquifer	0 wells	0 wells	
Upper confining unit	0 wells	0 wells		Upper confining unit	0 wells	0 wells	
Upper Floridan	80 wells	53 wells	66%	Upper Floridan	691 wells	126 wells	18%
Bucatunna clay confining unit	0 wells	0 wells		Bucatunna clay confining unit	0 wells	0 wells	
Ocala-Avon Park lower-permeability zone	5 wells	3 wells	60%	Ocala-Avon Park lower-permeability zone	67 wells	18 wells	27%
Avon Park permeable zone	2 wells	1 wells	50%	Avon Park permeable zone	111 wells	10 wells	9%

**Geologic formation that the well bottom lies within**	Pre-1910			**Geologic formation that the well bottom lies within**	Post-2010		
	Wells (n)	Flowing artesian wells (n)	Percentage of wells that are flowing artesian (%)		Wells (n)	Flowing artesian wells (n)	Percentage of wells that are flowing artesian (%)
Lisbon-Avon Park composite unit	15 wells	8 wells	53%	Lisbon-Avon Park composite unit	67 wells	9 wells	13%
Lower Floridan	35 wells	16 wells	46%	Lower Floridan	63 wells	1 wells	2%
Middle Avon Park composite unit	1 wells	1 wells	100%	Middle Avon Park composite unit	8 wells	0 wells	0%
Lower Avon Park permeable zone	2 wells	1 wells	50%	Lower Avon Park permeable zone	12 wells	5 wells	42%
Glauconite marker unit	2 wells	0 wells	0%	Glauconite marker unit	4 wells	2 wells	50%
Oldsmar permeable zone	2 wells	0 wells	0%	Oldsmar permeable zone	1 wells	1 wells	100%

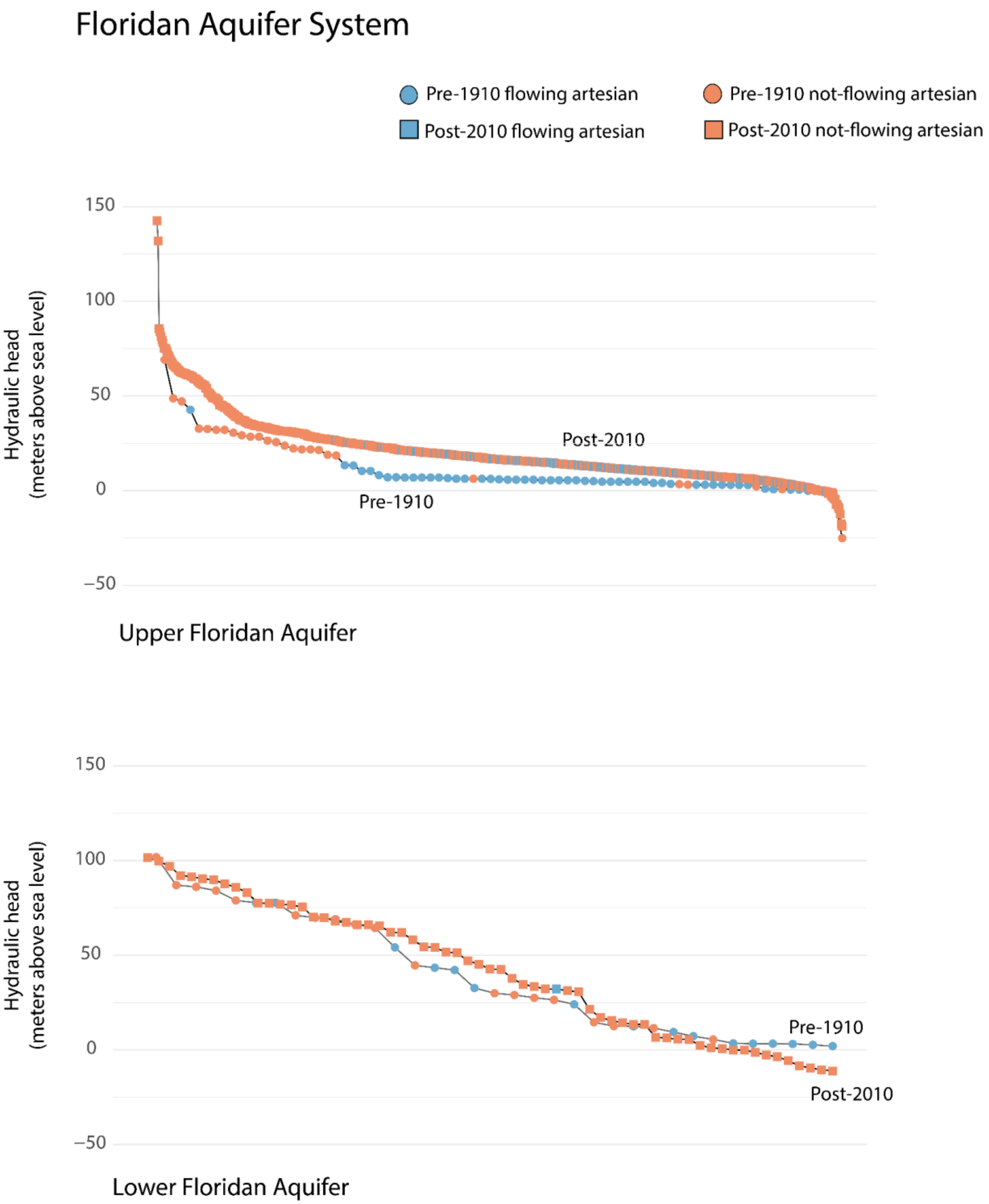

Fig. S14. Change of hydraulic head between our pre-1910 and post-2010 datasets in the Upper and Lower Floridan aquifers of the Floridan Aquifer System. Each point represents one well water level measurement, and these points are ranked from highest (left) to lowest (right) hydraulic head along the x-axis of the plot. Circles represent the pre-1910 time period and squares represent the post-2010 time period. Blue shaded points represent flowing artesian wells

while orange shaded points represent non-flowing wells. The pre-1910 median hydraulic head is 6 meters above sea level for the Upper Floridan Aquifer and 30 meters above sea level for the Lower Floridan Aquifer. The post-2010 median hydraulic head is 16 meters above sea level for the Upper Floridan and 43 meters above sea level for the Lower Floridan Aquifer. The pre-1910 median hydraulic head should be viewed as a minimum because we estimated the hydraulic head of flowing artesian wells to equate to the land surface elevation (but actual hydraulic heads for a flowing artesian well exceed the land surface elevation).

(e) Mississippi Embayment Regional Aquifer

In the Mississippi Embayment Regional Aquifer (Fig. 3E) in our pre-1910 dataset (n=579 wells) we classified 41% of these wells (n=238) as tapping confined conditions; 48.3% of these wells that tap confined conditions (n=115) are flowing artesian wells. The highest number of wells with flowing artesian conditions were found in the Lower Claiborne Confining Unit (n=38) and Middle Claiborne Aquifer (n=47). In our post-2010 dataset (n=3664 wells), we classified 18% (n=674) of these wells as tapping confined conditions; only 0.45% (n=3) of these wells that tap confined conditions are flowing artesian. Critically, we note that none of the wells with bottoms within the Middle Claiborne aquifer are flowing artesian in our post-2010 dataset, despite the large statistical sample of wells (n=542) in our post-2010 dataset for this unit.

Table S14. Mississippi Embayment Regional Aquifer flowing artesian conditions by geologic unit.

Geologic formation that the well bottom lies within	Pre-1910			Geologic formation that the well bottom lies within	Post-2010		
	Wells (n)	Flowing artesian wells (n)	Percentage of wells that are flowing artesian (%)		Wells (n)	Flowing artesian wells (n)	Percentage of wells that are flowing artesian (%)
Mississippi River Valley aquifer	0 wells	0 wells		Mississippi River Valley aquifer	0 wells	0 wells	
Vicksburg-Jackson Group confining unit	0 wells	0 wells		Vicksburg-Jackson Group confining unit	0 wells	0 wells	
Upper Claiborne aquifer	0 wells	0 wells		Upper Claiborne aquifer	0 wells	0 wells	
Middle Claiborne confining unit	0 wells	0 wells		Middle Claiborne confining unit	0 wells	0 wells	
Middle Claiborne aquifer	136 wells	47 wells	35%	Middle Claiborne aquifer	542 wells	0 wells	0%
Lower Claiborne confining unit	42 wells	38 wells	91%	Lower Claiborne confining unit	26 wells	0 wells	0%
Lower Claiborne aquifer	29 wells	15 wells	52%	Lower Claiborne aquifer	21 wells	1 wells	5%
Middle Wilcox aquifer	29 wells	14 wells	48%	Middle Wilcox aquifer	32 wells	1 wells	3%
Lower Wilcox aquifer	1 wells	1 wells	100%	Lower Wilcox aquifer	46 wells	0 wells	0%
Midway confining unit	1 wells	0 wells	0%	Midway confining unit	7 wells	1 wells	14%

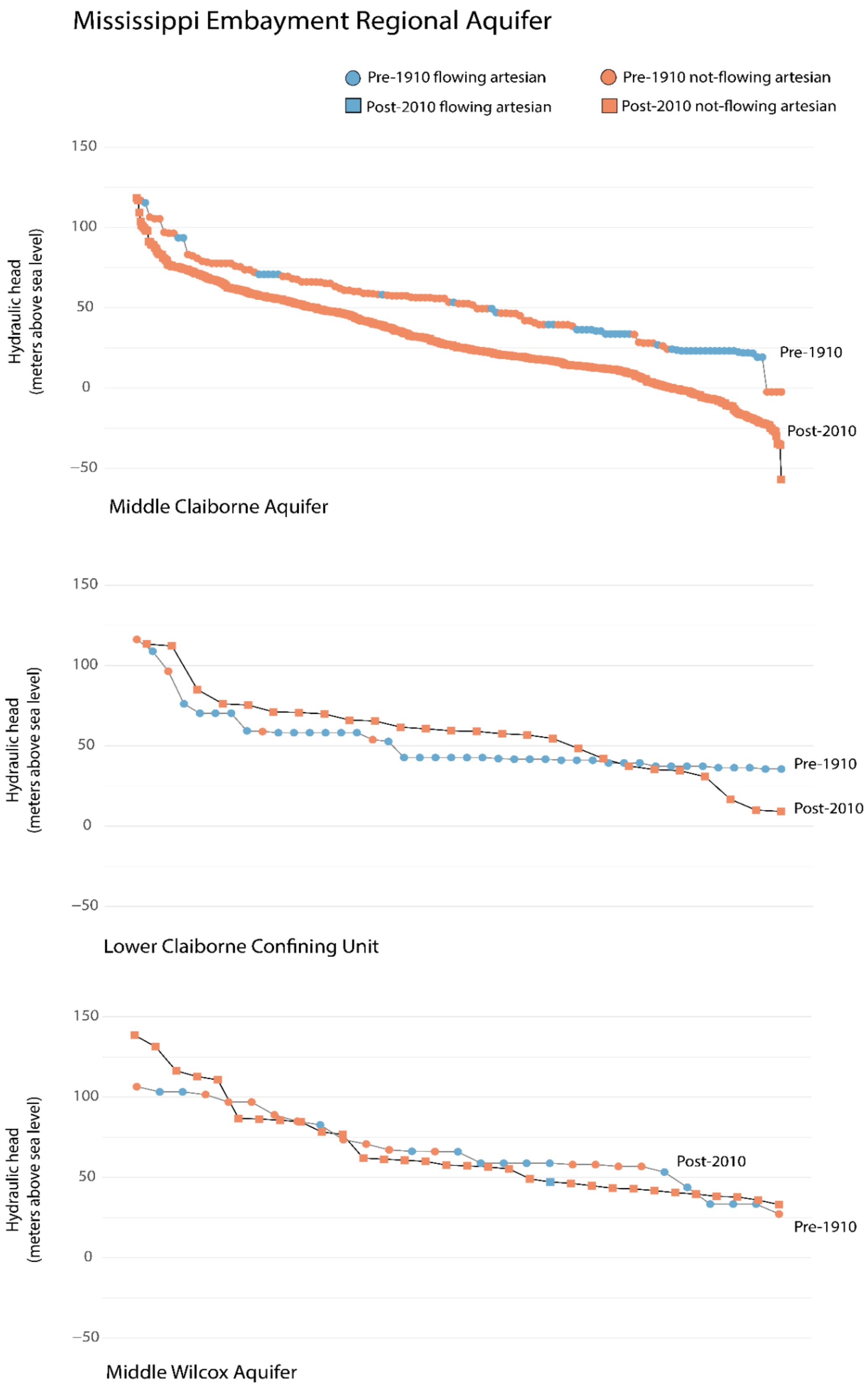

Fig. S15. Change of hydraulic head between our pre-1910 and post-2010 datasets in the

Middle Claiborne aquifer, Lower Claiborne confining unit, and the Middle Wilcox aquifer of the Mississippi Embayment Regional Aquifer System. Each point represents one well water level measurement, and these points are ranked from highest (left) to lowest (right) hydraulic head along the x-axis of the plot. Circles represent the pre-1910 time period and squares represent the post-2010 time period. Blue shaded points represent flowing artesian wells while orange shaded points represent non-flowing wells. The pre-1910 median hydraulic heads (in meters above sea level) are: 53, 43, and 66 meters for the Middle Claiborne aquifer, Lower Claiborne confining unit, and Middle Wilcox aquifer, respectively. Post-2010 medians (in meters above sea level) were 24, 59, and 57 meters for the Middle Claiborne aquifer, Lower Claiborne confining unit, and Middle Wilcox aquifer, respectively. The pre-1910 median hydraulic head should be viewed as a minimum because we estimated the hydraulic head of flowing artesian wells to equate to the land surface elevation (but actual hydraulic heads for a flowing artesian well exceed the land surface elevation).

(f) Houston-Gulf Coast Aquifer System

In the Houston-Gulf Coast Aquifer System (Fig. 3F) in our pre-1910 dataset (n=62 wells), we classified 87% of wells (n=53) as tapping confined conditions; 96.2% of these wells that tap confined conditions (n=51) are flowing artesian. The majority of wells under confined conditions that also exhibit flowing artesian conditions have bottoms within the Chicot and Evangeline Aquifers. Post-2010 (n=719 wells), 88% of wells (n=630) were classified as tapping confined conditions, but artesian conditions have disappeared entirely from confined wells.

Table S15. Houston-Gulf Coast Aquifer System flowing artesian conditions by geologic unit.

Geologic formation that the well bottom lies within	Pre-1910			Geologic formation that the well bottom lies within	Post-2010		
	Wells (n)	Flowing artesian wells (n)	Percentage of wells that are flowing artesian (%)		Wells (n)	Flowing artesian wells (n)	Percentage of wells that are flowing artesian (%)
Chicot (surficial)	26 wells	26 wells	100%	Chicot (surficial)	69 wells	0 wells	0%
Evangeline aquifer	21 wells	20 wells	95%	Evangeline aquifer	394 wells	0 wells	0%
Burkeville confining unit	2 wells	2 wells	100%	Burkeville confining unit	26 wells	0 wells	0%
Jasper aquifer	2 wells	2 wells	100%	Jasper aquifer	124 wells	0 wells	0%
Catahoula confining unit	2 wells	1 wells	50%	Catahoula confining unit	17 wells	0 wells	0%

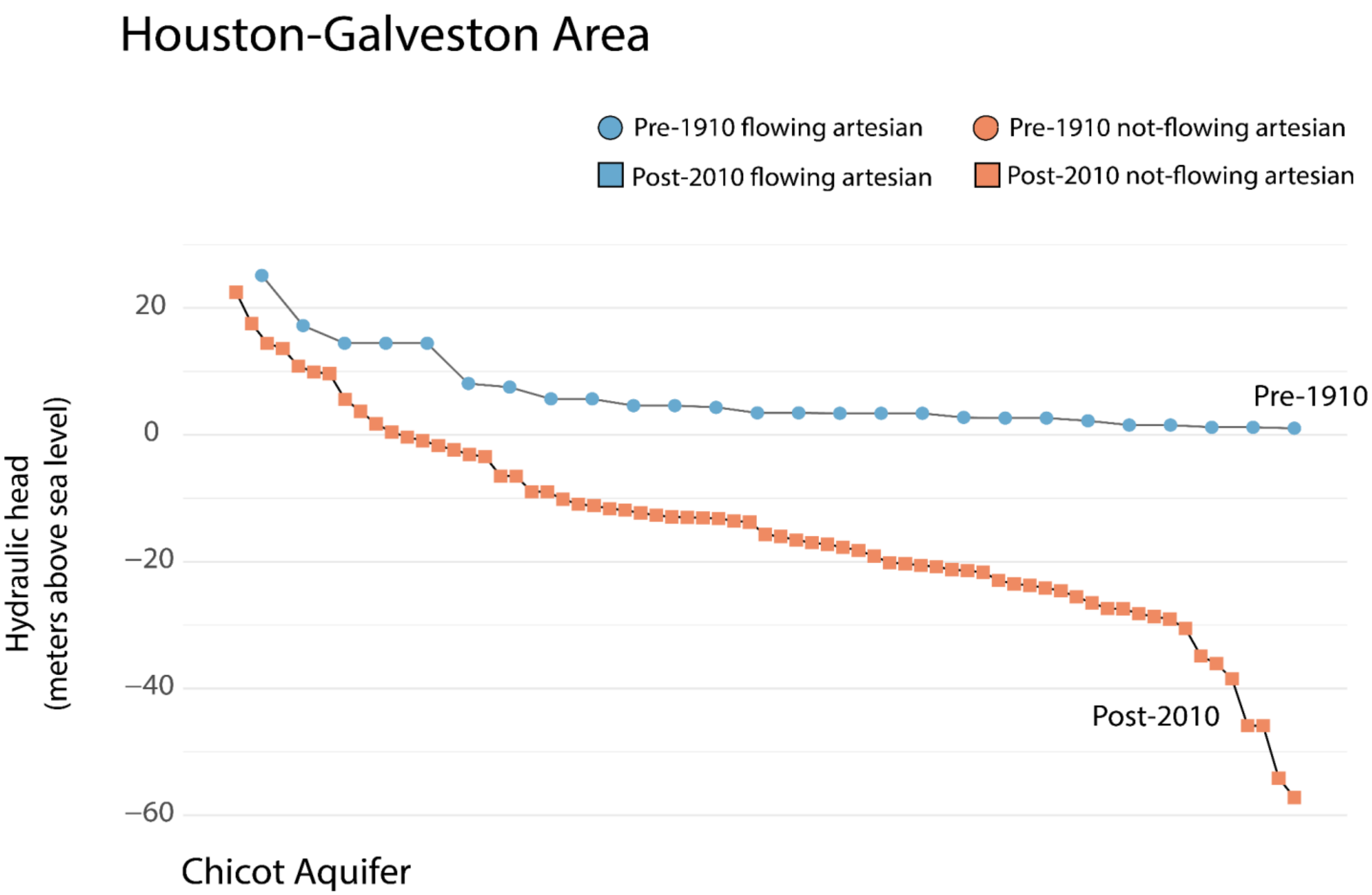

Fig. S16. **Change of hydraulic head between our pre-1910 and post-2010 datasets in the Chicot aquifer of the Houston-Galveston area.** Each point represents one well water level measurement, and these points are ranked from highest (left) to lowest (right) hydraulic head along the x-axis of the plot. Circles represent the pre-1910 time period and squares represent the post-2010 time period. Blue shaded points represent flowing artesian wells while orange shaded points represent non-flowing wells. The pre-1910 median hydraulic head is 4 meters above sea level; the post-2010 median hydraulic head is 16 meters below sea level (or, −16 m above sea level). The pre-1910 median hydraulic head should be viewed as a minimum because we estimated the hydraulic head of flowing artesian wells to equate to the land surface elevation (but actual hydraulic heads for a flowing artesian well exceed the land surface elevation).

(g) Roswell Artesian Basin

In the Roswell Artesian Basin (Fig. 3G) in our pre-1910 well water level dataset (n=386 wells), we classified 64% of wells (n=248) as tapping confined conditions; all (i.e., 100%) of these confined wells (n=248) are flowing artesian. In our post-2010 dataset (n=92 wells), we classified 35% of wells (n=32) as tapping confined conditions; none (i.e., 0%) demonstrate flowing artesian conditions.

Table S16. Roswell Artesian Basin flowing artesian conditions by geologic unit.

**Geologic formation that the well bottom lies within**	**Pre-1910**			**Geologic formation that the well bottom lies within**	**Post-2010**		
	Wells (n)	Flowing artesian wells (n)	Percentage of wells that are flowing artesian (%)		Wells (n)	Flowing artesian wells (n)	Percentage of wells that are flowing artesian (%)
Alluvium	137 wells	0 wells	0%	Alluvium	58 wells	0 wells	0%
Artesia	249 wells	248 wells	99.6%	Artesia	34 wells	0 wells	0%

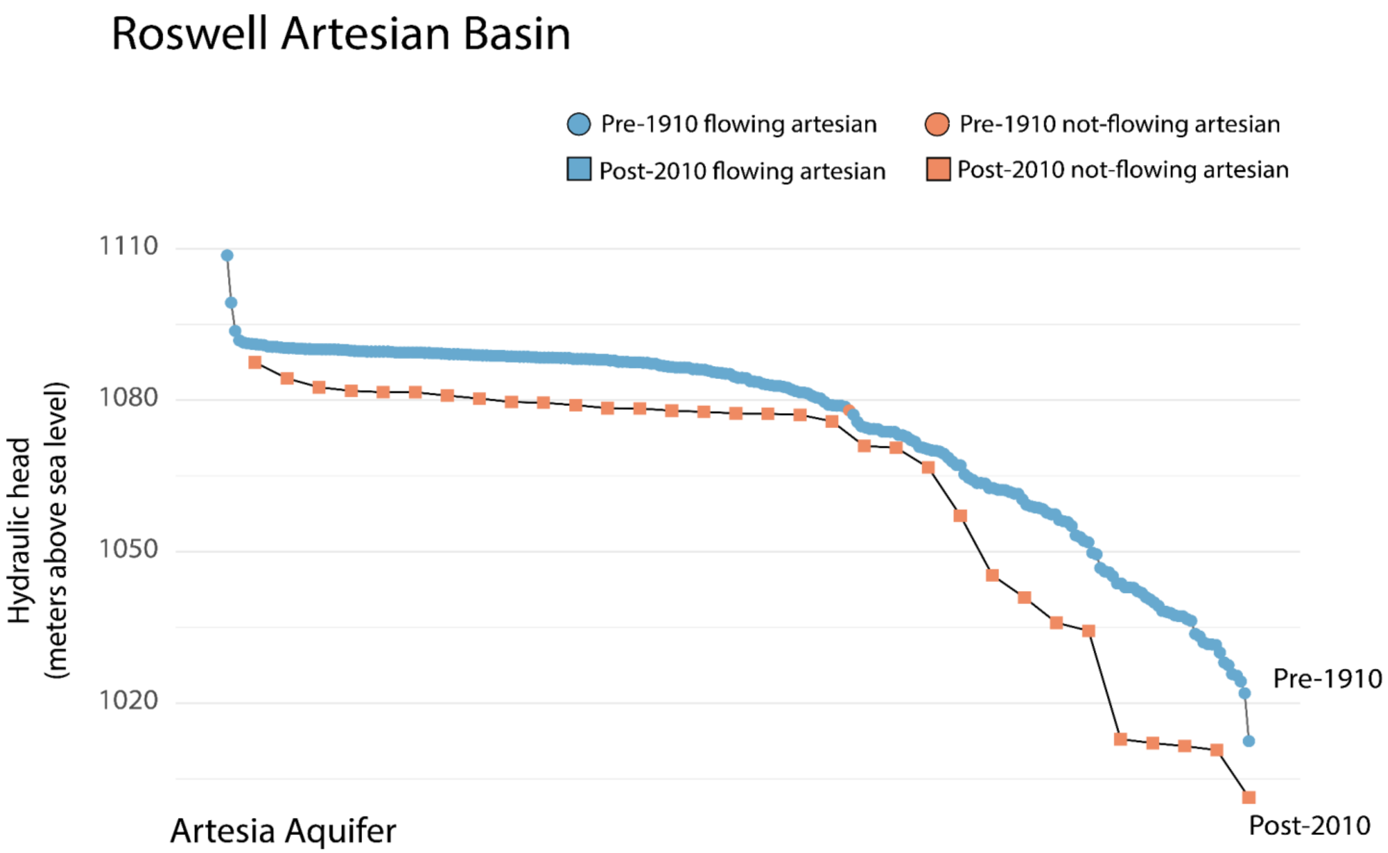

Fig. S17. Change of hydraulic head between our pre-1910 and post-2010 datasets in the Artesia aquifer of the Roswell Artesian Basin. Each point represents one well water level measurement, and these points are ranked from highest (left) to lowest (right) hydraulic head along the x-axis of the plot. Circles represent the pre-1910 time period and squares represent the post-2010 time period. Blue shaded points represent flowing artesian wells while orange shaded points represent non-flowing wells. The pre-1910 median hydraulic head is 1084 meters above sea level; the post-2010 median hydraulic head is 1077 meters above sea level. The pre-1910 median hydraulic head should be viewed as a minimum because we estimated the hydraulic head of flowing artesian wells to equate to the land surface elevation (but actual hydraulic heads for a flowing artesian well exceed the land surface elevation).

(h) Central Valley Aquifer

In the Central Valley Aquifer (Fig. 3H) in our pre-1910 dataset (n=4026 wells), we classified 7.7% of wells (n=310) as tapping confined conditions; 76.5% of these wells that tap confined conditions (n=237) are flowing artesian. In our post-2010 dataset (n=6239 wells), we classified 41% of wells (n=2571) as tapping confined conditions; 0.2% (n=6) are flowing artesian.

Table S17. Central Valley Aquifer flowing artesian conditions of confined geologic unit.

Geologic formation that the well bottom lies within	Pre-1910			Geologic formation that the well bottom lies within	Post-2010		
	Wells (n)	Flowing artesian wells (n)	Percentage of wells that are flowing artesian (%)		Wells (n)	Flowing artesian wells (n)	Percentage of wells that are flowing artesian (%)
Confined	310 wells	237 wells	77%	Confined	2571 wells	6 wells	0.2%

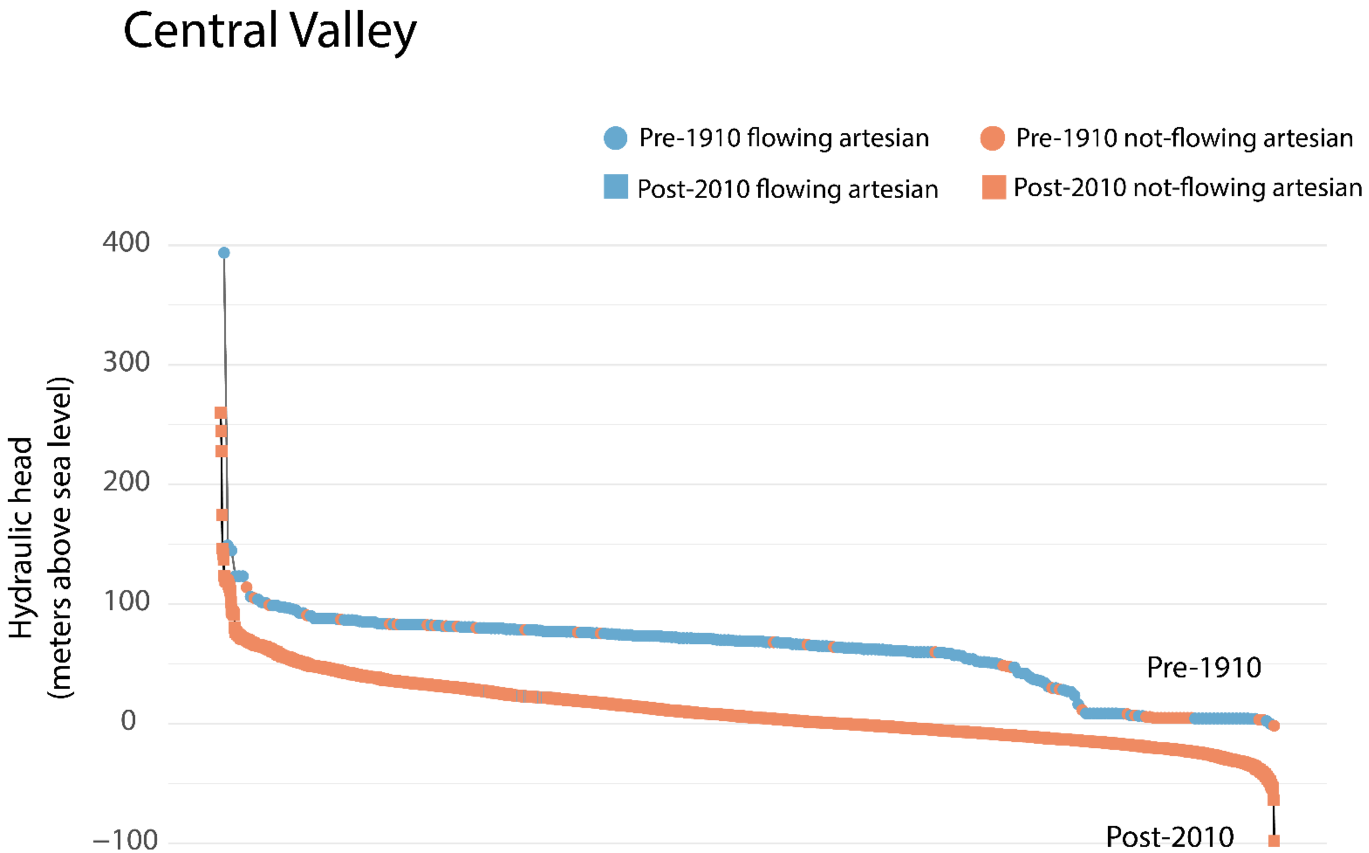

Fig. S18. Change of hydraulic head between our pre-1910 and post-2010 datasets of confined wells in the Central Valley. Each point represents one well water level measurement, and these points are ranked from highest (left) to lowest (right) hydraulic head along the x-axis of the plot. Circles represent the pre-1910 time period and squares represent the post-2010 time period. Blue shaded points represent flowing artesian wells while orange shaded points represent non-flowing wells. The pre-1910 median hydraulic head is 69 meters above sea level; the post-2010 median hydraulic head is 6 meters above sea level. The pre-1910 median hydraulic head should be viewed as a minimum because we estimated the hydraulic head of flowing artesian wells to equate to the land surface elevation (but actual hydraulic heads for a flowing artesian well exceed the land surface elevation).

S5. Hydrogeological conditions in our 62 aquifer systems

Here we detail the depth to confined conditions for each of our n=62 study aquifers and the hydrogeologic cross sections that we visually inspected to familiarize ourselves with each study area.

Table S19. Depth to confined conditions estimated for each of our n=62 regional aquifer systems

**Area**	**Broader aquifer system**	**Depth below which most wells classified* by the US Geological Survey are classified as confined (see Methods; units are meters below land surface)**	**Local-scale study and hydrogeologic cross section that we visually inspected to familiarize ourselves with the study area**
Central Allegheny Plateau	Appalachian Plateaus	~90 m	Fig. 2 of (*109*)
Northern Allegheny Plateau	Appalachian Plateaus	~60 m	Fig. 5 of (*110*)
Sacramento Basin	California Central Valley	~100 m	Fig. 4 of (*111*)
San Joaquin Basin	California Central Valley	~160 m	Fig. 10 of (*112*)
Tulare Basin	California Central Valley	~180 m	Fig. 2 of (*113*)
Central Carrizo-Wilcox	Carrizo-Wilcox	~60 m	Fig. 15 of (*114*)
Eastern Carrizo-Wilcox	Carrizo-Wilcox	~30 m	Fig. 2.20 of (*115*)
Western Carrizo-Wilcox	Carrizo-Wilcox	~70 m	Fig. 2.20 of (*115*)
Bluffton Till Plain	Central Lowland Till Plain	~30 m	Fig. 2 of (*116*)
Central Wabash and Bloomington Ridged Plain	Central Lowland Till Plain	~40 m	Fig. 8 of (*117*)
Iroquois Till Plains	Central Lowland Till Plain	~10 m	Fig. 4 of (*118*)
Tipton Till Plain	Central Lowland Till Plain	~40 m	Fig. 2 of (*119*)
Umatilla Basin and Horse Heaven Hills	Columbia Plateau Regional Aquifer System	~70 m	Fig. 7b of (*85*)
Balcones Fault Zone	Edwards-Trinity Aquifer System	~200 m	Plate 8 of (*120*)
Bacon Terrace	Floridan Aquifer System	~20 m	Fig. 3 of (*121*)
Dougherty Plain and Marianna Lowlands	Floridan Aquifer System	~40 m	Fig. 3 of (*122*)
Eastern Flatwoods Southshores	Floridan Aquifer System	~120 m	Fig. 5 of (*123*)

Area	Broader aquifer system	Depth below which most wells classified* by the US Geological Survey are classified as confined (see Methods; units are meters below land surface)	Local-scale study and hydrogeologic cross section that we visually inspected to familiarize ourselves with the study area
Intermediate Aquifer	Floridan Aquifer System	~50 m	Fig. 6 of (*124*)
Lower Coastal Plain	Floridan Aquifer System	~100 m	Fig. 11 of (*125*)
Ocala Uplift	Floridan Aquifer System	~40 m	Fig. 4 of (*126*)
Sea Island	Floridan Aquifer System	~40 m	Fig. 6 of (*127*)
Tifton Upland	Floridan Aquifer System	~50 m	Fig. 4 of (*126*)
Vidalia Upland	Floridan Aquifer System	~20 m	Fig. 3 of (*121*)
Alabama Coastal Lowlands	Gulf Coast Regional Aquifer System	~30 m	Fig. 8 of (*128*)
Catahoula Area	Gulf Coast Regional Aquifer System	~240 m	Fig. 5 of (*129*)
Gonzales-New Orleans Aquifer	Gulf Coast Regional Aquifer System	~90 m	Fig. 3 of (*130*)
Houston-Galveston Area	Gulf Coast Regional Aquifer System	~30 m	Fig. 2 of (*90*)
Lafayette Area	Gulf Coast Regional Aquifer System	~30 m	Fig. 3 of (*131*)
Southern Hills	Gulf Coast Regional Aquifer System	~30 m	Fig. 3 of (*132*)
San Luis Valley	Middle Rio Grande	~160 m	Fig. 5 of (*133*)
Central Mississippi Embayment	Mississippi Embayment	~80 m	Fig. 8 of (*103*)
Confined Claiborne Near Jackson	Mississippi Embayment	~280 m	Fig. 2 of (*134*)
Eastern Mississippi Embayment	Mississippi Embayment	~80 m	Fig. 5 of (*135*)
Western Mississippi Embayment	Mississippi Embayment	~40 m	Fig. 4 of (*136*)
Delmarva Peninsula	North Atlantic Coastal Plain	~40 m	Fig. 21 of (*137*)
Maryland Western Shores	North Atlantic Coastal Plain	~40 m	Fig. 4 of (*138*)
New Jersey Coastal Plain	North Atlantic Coastal Plain	~90 m	Fig. 29 of (*139*)
North Carolina and Virginia Coastal Plain	North Atlantic Coastal Plain	~40 m	Fig. 4 of (*140*)
Powder River Basin	Northern Great Plains	~70 m	Fig. 4 of (*141*)
Williston Basin	Northern Great Plains	~160 m	Fig. 4 of (*141*)
Eastern Cambrian-Ordovician Aquifers	Northern Midwest Aquifer System	~160 m	Fig. 20 of (*4*)
Eastern Silurian-Devonian Aquifers	Northern Midwest Aquifer System	~40 m	Fig. 9 of (*142*)

**Area**	**Broader aquifer system**	**Depth below which most wells classified* by the US Geological Survey are classified as confined (see Methods; units are meters below land surface)**	**Local-scale study and hydrogeologic cross section that we visually inspected to familiarize ourselves with the study area**
Mississippian-Silurian-Devonian Carbonates	Northern Midwest Aquifer System	~70 m	Fig. 4 of (*143*)
Northeast Missouri Carbonates	Northern Midwest Aquifer System	~60 m	Plate 1 of (*4*)
Northern Cambrian-Ordovician Aquifers	Northern Midwest Aquifer System	~180 m	Fig. 6 of (*144*)
Upper Carbonate Aquifer	Northern Midwest Aquifer System	~30 m	Fig. 4 of (*143*)
Western Cambrian-Ordovician Aquifers	Northern Midwest Aquifer System	~40 m	Fig. 2 of (*145*)
Northcentral Valley and Ridge	Valley and Ridge Aquifer System	~180 m	Fig. 3 of (*146*)
Southern Valley and Ridge	Valley and Ridge Aquifer System	~10 m	Fig. 3 of (*147*)
Antlers Aquifer	-	~70 m	Fig. 7.2-1 of (*148*)
Black Hills Uplift	-	~90 m	Fig. 15 of (*149*)
Black Warrior River Aquifer System	-	~10 m	Fig. 3 of (*150*)
Dakota Aquifer System	-	~60 m	Fig. 1 of (*86*)
Denver Basin	-	~180 m	Fig. 3 of (*151*)
Eastern Dakota Aquifer	-	~70 m	Fig. 1 of (*152*)
Long Island	-	~180 m	Fig. 70 of (*153*)
Michigan Basin	-	~50 m	Fig. 7 of (*154*)
Pearl and Chattahoochee Aquifer System	-	~30 m	Fig. 74 of (*155*)
Peedee and Black Creek and Cape Fear Aquifers	-	~40 m	Fig. 2 of (*156*)
Roswell Basin	-	~120 m	Fig. 4 of (*157*)
Salt Lake Valley	-	~60 m	Fig. 4 of (*158*)
Santa Clara Valley	-	~120 m	Fig. 17 of (*159*)

*See Methods section entitled 'Identifying wells that tap confined aquifers across the US (Figs. 4 and 5)'

S6. Continental US results pre-1910 to post-2010

Here are the full results of our continental scale analysis of pre-1910 to post-2010 flowing artesian conditions. Table S20 shows such conditions by each aquifer studied (n=62). These results correspond to figures 4 and 5 in the main text.

Table S20. Pre-1910 and post-2010 aquifer systems (n=62) flowing artesian conditions.

Aquifer System	Pre-1910			Post-2010		
	Wells (n)	Flowing artesian wells (n)	Percentage of wells that are flowing artesian (%)	Wells (n)	Flowing artesian wells (n)	Percentage of wells that are flowing artesian (%)
Alabama Coastal Lowlands	13 wells	12 wells	92%	10 wells	0 wells	0%
Antlers Aquifer	10 wells	0 wells	0%	9 wells	0 wells	0%
Bacon Terrace	5 wells	0 wells	0%	34 wells	0 wells	0%
Balcones Fault Zone	14 wells	8 wells	57%	11 wells	2 wells	18%
Black Hills Uplift	6 wells	6 wells	100%	45 wells	6 wells	13%
Black Warrior River Aquifer System (Eutaw and McShan Formations and Tuscaloosa Group)	139 wells	73 wells	53%	259 wells	3 wells	1%
Bluffton Till Plain	19 wells	10 wells	53%	19 wells	0 wells	0%
Catahoula Area	38 wells	35 wells	92%	58 wells	12 wells	21%
Central Allegheny Plateau	13 wells	6 wells	46%	8 wells	0 wells	0%
Central Carrizo-Wilcox	18 wells	10 wells	56%	6 wells	0 wells	0%
Central Mississippi Embayment	182 wells	123 wells	68%	309 wells	2 wells	0.7%
Central Wabash and Bloomington Ridged Plain	22 wells	11 wells	50%	39 wells	0 wells	0%
Confined Claiborne Near Jackson	6 wells	4 wells	67%	16 wells	0 wells	0%
Dakota Aquifer System	232 wells	202 wells	87%	218 wells	11 wells	5%
Delmarva Peninsula	18 wells	12 wells	67%	258 wells	5 wells	2%
Denver Basin	19 wells	11 wells	58%	22 wells	0 wells	0%

Aquifer System	Pre-1910			Post-2010		
	Wells (n)	Flowing artesian wells (n)	Percentage of wells that are flowing artesian (%)	Wells (n)	Flowing artesian wells (n)	Percentage of wells that are flowing artesian (%)
Dougherty Plain and Marianna Lowlands	17 wells	6 wells	35%	139 wells	0 wells	0%
Eastern Cambrian-Ordovician Aquifers	63 wells	42 wells	67%	16 wells	5 wells	31%
Eastern Carrizo-Wilcox	126 wells	24 wells	19%	110 wells	2 wells	2%
Eastern Dakota Aquifer	17 wells	2 wells	12%	14 wells	0 wells	0%
Eastern Flatwoods Southshores	14 wells	13 wells	93%	58 wells	26 wells	45%
Eastern Mississippi Embayment	37 wells	18 wells	49%	160 wells	2 wells	1%
Eastern Silurian-Devonian Aquifers	114 wells	44 wells	39%	65 wells	1 wells	2%
Gonzales-New Orleans Aquifer	5 wells	5 wells	100%	13 wells	3 wells	23%
Houston-Galveston Area	45 wells	39 wells	87%	702 wells	0 wells	0%
Intermediate Aquifer	10 wells	9 wells	90%	271 wells	71 wells	26%
Iroquois Till Plains	5 wells	0 wells	0%	6 wells	0 wells	0%
Lafayette Area	90 wells	46 wells	51%	530 wells	5 wells	0.9%
Long Island	4 wells	4 wells	100%	88 wells	6 wells	7%
Lower Coastal Plain	19 wells	14 wells	74%	14 wells	0 wells	0%
Maryland Western Shores	16 wells	3 wells	19%	337 wells	0 wells	0%
Michigan Basin	15 wells	7 wells	47%	13 wells	0 wells	0%
Mississippian-Silurian-Devonian Carbonates	120 wells	58 wells	48%	39 wells	0 wells	0%
New Jersey Coastal Plain	76 wells	69 wells	91%	606 wells	4 wells	0.7%
North Carolina and Virginia Coastal Plain	30 wells	22 wells	73%	240 wells	4 wells	2%
Northcentral Valley and Ridge	4 wells	1 wells	25%	11 wells	1 wells	9%
Northeast Missouri Carbonates	45 wells	8 wells	18%	13 wells	0 wells	0%

Aquifer System	Pre-1910			Post-2010		
	Wells (n)	Flowing artesian wells (n)	Percentage of wells that are flowing artesian (%)	Wells (n)	Flowing artesian wells (n)	Percentage of wells that are flowing artesian (%)
Northern Allegheny Plateau	8 wells	3 wells	38%	8 wells	0 wells	0%
Northern Cambrian-Ordovician Aquifers	9 wells	3 wells	33%	4 wells	0 wells	0%
Ocala Uplift	13 wells	1 wells	8%	226 wells	4 wells	2%
Pearl and Chattahoochee Aquifer System	47 wells	14 wells	30%	130 wells	0 wells	0%
Peedee and Black Creek and Cape Fear Aquifers	27 wells	22 wells	82%	14 wells	0 wells	0%
Powder River Basin	5 wells	2 wells	40%	10 wells	0 wells	0%
Roswell Basin	247 wells	246 wells	99%	40 wells	0 wells	0%
Sacramento Basin	6 wells	5 wells	83%	892 wells	6 wells	0.7%
Salt Lake Valley	8 wells	7 wells	88%	50 wells	7 wells	14%
San Joaquin Basin	72 wells	47 wells	65%	252 wells	0 wells	0%
San Luis Valley	8 wells	8 wells	100%	28 wells	19 wells	68%
Santa Clara Valley	7 wells	7 wells	100%	58 wells	11 wells	19%
Sea Island	80 wells	72 wells	90%	254 wells	108 wells	43%
Southern Hills	41 wells	18 wells	44%	694 wells	43 wells	6%
Southern Valley and Ridge	29 wells	3 wells	10%	10 wells	0 wells	0%
Tifton Upland	19 wells	2 wells	11%	130 wells	0 wells	0%
Tipton Till Plain	11 wells	9 wells	82%	5 wells	0 wells	0%
Tulare Basin	186 wells	149 wells	80%	1586 wells	0 wells	0%

Aquifer System	Pre-1910			Post-2010		
	Wells (n)	Flowing artesian wells (n)	Percentage of wells that are flowing artesian (%)	Wells (n)	Flowing artesian wells (n)	Percentage of wells that are flowing artesian (%)
Umatilla Basin and Horse Heaven Hills	9 wells	0 wells	0%	11 wells	1 wells	9%
Upper Carbonate Aquifer	8 wells	1 wells	13%	3 wells	0 wells	0%
Vidalia Upland	30 wells	11 wells	37%	129 wells	4 wells	3%
Western Cambrian-Ordovician Aquifers	48 wells	15 wells	31%	7 wells	0 wells	0%
Western Carrizo-Wilcox	29 wells	17 wells	59%	15 wells	1 wells	7%
Western Mississippi Embayment	126 wells	30 wells	24%	303 wells	2 wells	0.7%
Williston Basin	4 wells	4 wells	100%	19 wells	4 wells	21%

S7. Comparison of artesian conditions in regional and continental analysis

Here we compare our main results (i.e., the proportion of wells within an aquifer system's boundaries that exhibit flowing artesian conditions in our pre-1910 dataset and in our post-2010 dataset) when we apply our two different methods to identify wells that tap a confined aquifer: ***Method 1: Regional analyses*** (columns 2 and 3 in the table below; *based on hydrostratigraphic data that are available for eight aquifer systems – results presented in main text Fig. 3)* and ***Method 2: Continental US analysis*** (columns 4 and 5 in the table below; *based on vertical variability in the categories (e.g., confined, unconfined) that the US Geological Survey has ascribed to deeper versus shallower wells – results presented in main text Figs. 4, 5).*

Table S21. Comparison of regional and continental analysis flowing artesian conditions

**Aquifer System**	**Method 1: Regional analyses**		**Method 2: Continental US analysis**	
	Pre-1910 flowing artesian	Post-2010 flowing artesian	Pre-1910 flowing artesian	Post-2010 flowing artesian
Columbia Plateau Regional Aquifer System	2 %<br><br>(n=2 of n=87 wells are artesian)	1 %<br><br>(n=1 of n=105 wells are flowing artesian)	0 %<br><br>(n=0 of n=9 wells are artesian)	9 %<br><br>(n=1 of n=11 wells are artesian)
Dakota Aquifer	93 %<br><br>(n=162 of n=175 wells are artesian)	9 %<br><br>(n=6 of n=64 wells are artesian)	87 %<br><br>(n=202 of n=232 wells are artesian)	11 %<br><br>(n=11 of n=218 wells are artesian)
North Atlantic Coastal Plain Aquifer System	83 %<br><br>(n=113 of n=137 wells are artesian)	0.8 %<br><br>(n=14 of n=1670 wells are artesian)	76 %<br><br>(n=110 of n=144 wells are artesian)	1 %<br><br>(n=19 of n=1529 wells are artesian)
Floridan Aquifer System	58 %<br><br>(n=83 of n=144 wells are artesian)	17 %<br><br>(n=172 of n=1024 wells are artesian)	57 %<br><br>(n=136 of n=237 wells are artesian)	17 %<br><br>(n=213 of n=1246 are artesian)

**Aquifer System**	**Method 1: Regional analyses**		**Method 2: Continental US analysis**	
	Pre-1910 flowing artesian	Post-2010 flowing artesian	Pre-1910 flowing artesian	Post-2010 flowing artesian
Mississippi Embayment Regional Aquifer	48 %<br>(n=115 of n=238 wells are artesian)	0.5 %<br>(n=3 of n=674 wells are artesian)	50 %<br>(n=171 of n=345 wells are artesian)	0.8 %<br>(n=6 of n=772 wells are artesian)
Houston-Gulf Coast Aquifer System	96 %<br>(n=51 of n=53 wells are artesian)	0 %<br>(n=0 of n=630 wells are artesian)	87 %<br>(n=39 of n=45 wells are artesian)	0 %<br>(n=0 of n=702 wells are artesian)
Roswell Artesian Basin	100 %<br>(n=248 of n=248 wells are artesian)	0 %<br>(n=0 of n=32 wells are artesian)	100 %<br>(n=246 of n=247 wells are artesian)	0 %<br>(n=0 of n=40 wells are artesian)
Central Valley Aquifer	77 %<br>(n=237 of n=310 wells are artesian)	0.2 %<br>(n=6 of n=2571 wells are artesian)	76 %<br>(n=201 of n=264 are artesian)	0.2 %<br>(n=6 of n= 2730 wells are artesian)

S8. Climate and groundwater withdrawals by aquifer system

We compiled data of climatological and anthropogenic factors that may influence flowing artesian conditions in each of the aquifer systems studied (n=62). We examined aridity, precipitation, and annual groundwater withdrawals.

Table S22. Aridity, precipitation, annual groundwater withdrawals as compared to pre-1910 and post-2010 flowing artesian conditions of all aquifer systems studied (n=62). Area and aquifer systems delineated by (*83*)**, aridity index by** (*54*)**, precipitation** (*53*)**, and annual groundwater withdrawals (2015) by** (*41*)**.**

Area	Broader aquifer system	Pre-1910 proportion of wells that exhibit flowing artesian conditions	Post-2010 proportion of wells that exhibit flowing artesian conditions	Aridity index (Annual precipitation divided by annual potential evapotranspiration)	Annual precipitation (mm/year)	Annual groundwater withdrawals (mm/year)
Central Allegheny Plateau	Appalachian Plateaus	46%	0%	0.94	1181	5
Northern Allegheny Plateau	Appalachian Plateaus	38%	0%	0.98	1123	5
Sacramento Basin	California Central Valley	83%	0%	0.29	580	338
San Joaquin Basin	California Central Valley	65%	0%	0.18	345	312
Tulare Basin	California Central Valley	80%	0%	0.10	215	298
Central Carrizo-Wilcox	Carrizo-Wilcox	56%	0%	0.57	1044	11
Eastern Carrizo-Wilcox	Carrizo-Wilcox	19%	2%	0.75	1283	6
Western Carrizo-Wilcox	Carrizo-Wilcox	59%	7%	0.33	665	10
Bluffton Till Plain	Central Lowland Till Plain	53%	0%	0.82	1025	7
Central Wabash and Bloomington Ridged Plain	Central Lowland Till Plain	50%	0%	0.82	1007	7
Iroquois Till Plains	Central Lowland Till Plain	0	0%	0.85	1010	4
Tipton Till Plain	Central Lowland Till Plain	82%	0%	0.88	1085	14
Umatilla Basin and Horse Heaven Hills	Columbia Plateau Regional Aquifer System	0%	9%	0.23	371	26

Area	Broader aquifer system	Pre-1910 proportion of wells that exhibit flowing artesian conditions	Post-2010 proportion of wells that exhibit flowing artesian conditions	Aridity index (Annual precipitation divided by annual potential evapotranspiration)	Annual precipitation (mm/year)	Annual groundwater withdrawals (mm/year)
Balcones Fault Zone	Edwards-Trinity Aquifer System	57%	18%	0.37	765	55
Bacon Terrace	Floridan Aquifer System	0%	0%	0.78	1247	11
Dougherty Plain and Marianna Lowlands	Floridan Aquifer System	35%	0%	0.88	1464	26
Eastern Flatwoods Southshores	Floridan Aquifer System	93%	45%	0.70	1342	32
Intermediate Aquifer	Floridan Aquifer System	90%	26%	0.70	1381	38
Lower Coastal Plain	Floridan Aquifer System	74%	0%	0.78	1308	7
Ocala Uplift	Floridan Aquifer System	8%	2%	0.82	1399	25
Sea Island	Floridan Aquifer System	90%	43%	0.80	1313	23
Tifton Upland	Floridan Aquifer System	11%	0%	0.80	1306	16
Vidalia Upland	Floridan Aquifer System	37%	3%	0.74	1212	9
Alabama Coastal Lowlands	Gulf Coast Regional Aquifer System	92%	0%	1.03	1649	13
Catahoula Area	Gulf Coast Regional Aquifer System	92%	21%	1.04	1596	8
Gonzales-New Orleans Aquifer	Gulf Coast Regional Aquifer System	100%	23%	1.03	1662	23
Houston-Galveston Area	Gulf Coast Regional Aquifer System	87%	0%	0.73	1324	23
Lafayette Area	Gulf Coast Regional Aquifer System	51%	0.90%	0.94	1534	25
Southern Hills	Gulf Coast Regional Aquifer System	44%	6%	1.05	1614	15
San Luis Valley	Middle Rio Grande	100%	68%	0.18	272	44
Central Mississippi Embayment	Mississippi Embayment	68%	0.70%	0.88	1371	188
Confined Claiborne Near Jackson	Mississippi Embayment	67%	0%	0.96	1481	13
Eastern Mississippi Embayment	Mississippi Embayment	49%	1%	0.96	1440	10

Area	Broader aquifer system	Pre-1910 proportion of wells that exhibit flowing artesian conditions	Post-2010 proportion of wells that exhibit flowing artesian conditions	Aridity index (Annual precipitation divided by annual potential evapotranspiration)	Annual precipitation (mm/year)	Annual groundwater withdrawals (mm/year)
Western Mississippi Embayment	Mississippi Embayment	24%	0.70%	0.91	1426	4
Delmarva Peninsula	North Atlantic Coastal Plain	67%	2%	0.79	1176	20
Maryland Western Shores	North Atlantic Coastal Plain	19%	0%	0.76	1173	24
New Jersey Coastal Plain	North Atlantic Coastal Plain	91%	0.70%	0.87	1202	39
North Carolina and Virginia Coastal Plain	North Atlantic Coastal Plain	73%	2%	0.84	1272	6
Powder River Basin	Northern Great Plains	40%	0%	0.24	397	2
Williston Basin	Northern Great Plains	100%	21%	0.29	419	1
Eastern Cambrian-Ordovician Aquifers	Northern Midwest Aquifer System	67%	31%	0.79	960	10
Eastern Silurian-Devonian Aquifers	Northern Midwest Aquifer System	39%	2%	0.80	927	15
Mississippian-Silurian-Devonian Carbonates	Northern Midwest Aquifer System	48%	0%	0.75	952	8
Northeast Missouri Carbonates	Northern Midwest Aquifer System	18%	0%	0.74	1035	4
Northern Cambrian-Ordovician Aquifers	Northern Midwest Aquifer System	33%	0%	0.78	864	15
Upper Carbonate Aquifer	Northern Midwest Aquifer System	13%	0%	0.74	899	4
Western Cambrian-Ordovician Aquifers	Northern Midwest Aquifer System	31%	0%	0.76	859	11
Northcentral Valley and Ridge	Valley and Ridge Aquifer System	25%	9%	0.92	1168	3
Southern Valley and Ridge	Valley and Ridge Aquifer System	10%	0%	1.03	1434	7
Antlers Aquifer	-	0%	0%	0.70	1178	3
Black Hills Uplift	-	100%	13%	0.32	528	2

Area	Broader aquifer system	Pre-1910 proportion of wells that exhibit flowing artesian conditions	Post-2010 proportion of wells that exhibit flowing artesian conditions	Aridity index (Annual precipitation divided by annual potential evapotranspiration)	Annual precipitation (mm/year)	Annual groundwater withdrawals (mm/year)
Black Warrior River Aquifer System (Eutaw and McShan Formations and Tuscaloosa Group)	-	53%	1%	0.96	1437	5
Dakota Aquifer System	-	87%	67%	0.38	566	3
Denver Basin	-	58%	0%	0.22	425	8
Eastern Dakota Aquifer	-	12%	0%	0.63	808	6
Long Island	-	100%	7%	0.87	1211	158
Michigan Basin	-	47%	0%	0.78	883	8
Pearl and Chattahoochee Aquifer System	-	30%	0%	0.83	1352	9
Peedee and Black Creek and Cape Fear Aquifers	-	82%	0%	0.82	1306	8
Roswell Basin	-	99%	0%	0.14	313	45
Salt Lake Valley	-	88%	14%	0.26	457	128
Santa Clara Valley	-	100%	19%	0.27	447	134

S9. Statistical analyses using Generalized Linear Mixed Models (GLMM)

Our data in this study consists of binary flowing artesian designations for two time periods, pre-1910 and post-2010. We were able to compile three sets of possible explanatory variables: the aridity index, annual precipitation (mm/year), annual groundwater withdrawals (mm/year for year 2015), as described in S8. These data are derived from relatively recent measurements (aridity index 1970-2000; annual precipitation 1991-2020; annual groundwater withdrawals 2015). Therefore, we only apply them to our post-2010 time period (and not our pre-1910 time period).

We designed two analyses for our data. We performed our analyses in R using the package `lme4` (Linear Mixed-Effects Models using 'Eigen' and S4; (*160*).

Analysis 1: Does the proportion of wells exhibiting flowing artesian conditions change with time period?

N0: The time period does not matter
N1: The time period matters, time period is an explanatory variable

We used a GLMM with random effects to account for differences that may be inherent to the individual aquifer systems. The Bayesian Information Criterion (BIC) values were 3169 and 814 for the null and hypothesis, respectively. The delta BIC is 2355. With a dBIC > 10 there is strong evidence that the time period difference has resulted in demonstrably different values of the well flowing. This result makes intuitive sense to us and is as we expected. We then move to examining our other covariates in analysis 2.

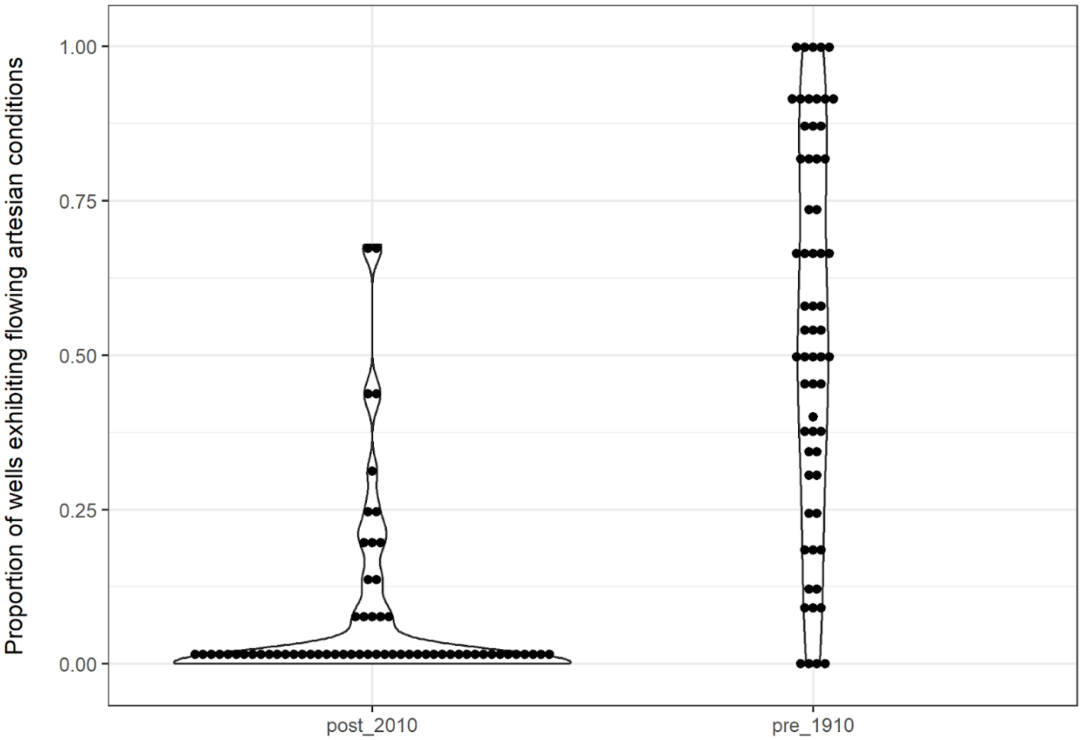

Fig. S19. The distribution of the proportion of wells exhibiting flowing artesian conditions for both time periods (pre-1910, post-2010). Each point represents one of our n=62 aquifer systems for each time interval. The plot provides a clear visual impression that the proportion of wells exhibiting flowing artesian conditions is higher for the pre-1910 time interval (points in the right column) than the post-2010 time interval (points in the left column).

Analysis 2: Do covariates of aridity, precipitation, and groundwater withdrawals change the proportion of flowing artesian wells?

N0: None of the covariates explain the proportion of wells exhibiting flowing artesian conditions

N1: A single covariate explains the proportion of wells exhibiting flowing artesian conditions

-Aridity

-Precipitation

-Groundwater withdrawals

N2: Two of the three covariates explain the proportion of wells exhibiting flowing artesian conditions

-Aridity & precipitation

-Aridity & groundwater withdrawals

-Groundwater withdrawals and precipitation

N3: All three covariates explain the proportion of wells exhibiting flowing artesian conditions

-Aridity & precipitation & groundwater withdrawals

For the N2 and N3 models, all were tested with and without interactions. Due to the nature of our covariate data (as discussed above), these models were only tested on the post-2010 time period.

Table S23. BIC and dBIC of analysis 2 examining covariates of aridity, precipitation, and groundwater withdrawals on the proportion of wells exhibiting flowing artesian conditions in the post-2010 time period (n=9,644 wells across n=62 aquifer systems).

Model	BIC	dBIC	K (parameters)
M0 (null)	1181	221	1
Single Covariates			
M1: aridity	1185	225	2
M2: precipitation	1176	216	2
M3: groundwater withdrawals	1123	162	2
Two covariates			
M4: aridity & precipitation	1091	131	3
M5: aridity & groundwater withdrawals	1085	125	3
M6: groundwater withdrawals & precipitation	1122	161	3
Two covariates with interactions			
M4i: aridity & precipitation with interactions	1095	134	4
M5i: aridity & groundwater withdrawals with interactions	1079	118	4
M6i: groundwater withdrawals & precipitation with interactions	1118	158	4
Three covariates			
M7: aridity & precipitation & groundwater withdrawals	997	36	4
Three covariates with interactions			
M7i: aridity & precipitation & groundwater with interactions	960	0 (BEST MODEL)	8

The ‘best’ model (i.e., defined as the model with the lowest BIC, in this case: BIC=960) from this analysis is the most complex model with all three covariates and interactions (M7i). All of the other models have dBIC > 7; thus, we dismiss these remaining models as the evidence against these models is strong. Model fit can be assessed visually by comparing the observed and predicted values for the proportion of wells exhibiting flowing artesian conditions under M7i. Deviation from the 1:1 line indicates the model may be best, but does not have a good fit. Here we observe that the data and model identify low proportions (most <0.4) of wells exhibiting flowing artesian conditions, but there is considerable variation in the predicted value of the proportion.

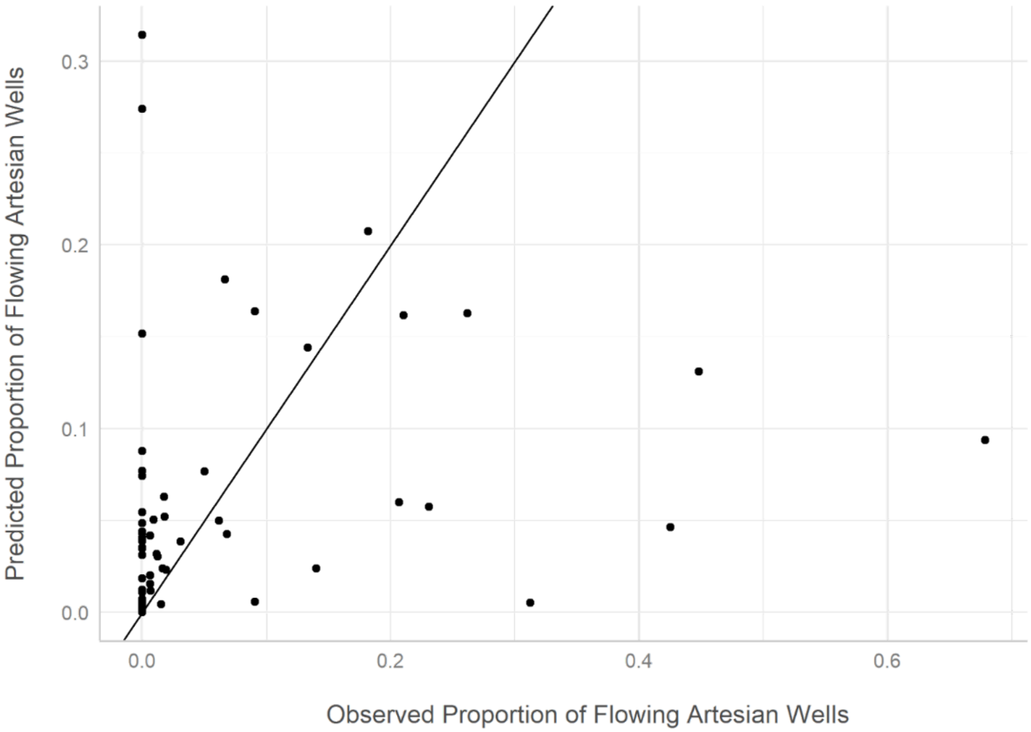

Fig. S20. The observed proportion of wells exhibiting flowing artesian conditions vs the predicted proportion of wells exhibiting flowing artesian conditions for model M7i (post-2010 time period). Each point represents one of our n=62 aquifer systems. The y-axis values represent the predicted (statistical model) proportion of wells exhibiting flowing artesian conditions. The x-axis values represent the actual proportion of wells exhibiting flowing artesian conditions based on our compiled data.

Further, when we visually examine interaction effects of the model (three-way interactions between aridity, precipitation, and groundwater withdrawals on the proportion of wells exhibiting flowing artesian conditions), we find that the results are generally counterintuitive to what we might hypothesize given the covariates. We conclude that while model M7i is best among the suite of hypotheses tested, there is a need for additional variables to test additional

hypotheses to explain the proportion of wells exhibiting flowing artesian conditions. We have identified two possible explanations for model's behavior:

(1) Due to the modern time period the covariate data represent, we believe that it is only appropriate to apply the covariates to the post-2010 dataset and not to our pre-1910 dataset. However, the post-2010 dataset (which examines 62 aquifer systems), contains n=9,644 total wells, of which only n=381 are flowing artesian wells. Because flowing artesian wells are so uncommon in the post-2010 time period, we believe that our post-2010 dataset of flowing artesian well prevalence may represent a rare event problem in the model. That is, there are not enough flowing artesian wells compared to non-flowing wells for our model to accurately predict the proportion of wells exhibiting flowing artesian conditions in the post-2010 time period.

(2) A key variable missing in our analyses are the specific storages of the aquifer systems. Hydraulic properties are critically important to understand aquifer units and systems; one of the most important variables is specific storage, defined as the volume of water released from storage per unit volume of saturated material. We attempted to compile the specific storage values for all of our aquifer systems, but we discovered that information on the hydraulic properties of our aquifer systems are difficult to find in the literature. For our eight regional aquifer systems, which are all well studied aquifer systems with 3D hydrogeologic models available (excluding the Dakota, which did not have a 3D hydrogeologic model), we were able to find specific storage (or storage coefficient) values for seven of the eight systems. However, for those systems there are issues with the consistency and nomenclature of specific storage (sometimes reported as specific storage, sometimes storage coefficient, etc.). Though we could not develop a compilation of specific storage values that we were sufficiently confident in for analyses, we stress that specific storage could be a critical addition and may improve the statistical model, potentially yielding better understanding of changes in the proportion of wells exhibiting flowing artesian conditions between the two time periods. The compilation of such data would be an invaluable contribution for future work.

As discussed earlier, we believe there may be a rare event problem in our post-2010 model. We believe it is likely that there would not be a rare event problem if we were able to generate a model for our pre-1910 time period (as the total number of flowing artesian wells observed is much higher than our post-2010 time period). However, we are unable to test this theory as we do not currently have covariate data for the pre-1910 time period. To our knowledge, observational data for aridity, annual precipitation, and annual groundwater withdrawals prior to 1910 do not exist continentally for the United States. It is possible that some measurements exist in historical reports, but these measurements have yet to be made widely available. One possible solution to the issue of these missing data could be to use simulated values from climatological and hydrological models. This would be an interesting area of future work but is unfortunately beyond the scope of our study.

S10. Early 1900s data compilation and quality control

We compiled thousands of water level measurements reported in tables within US Geological Survey reports published in the early 1900s (*3*, *57–60*)*.

Our search for data sources took place from May-June 2021; our data compilation and transcription took place from June-October 2021; our data cleaning occurred in November 2021.

*our transcription of (*58*) is incomplete in the state of Texas. The following counties were not transcribed: Anderson, Angelina, Camp, Cherokee, Franklin, Gregg, Hopkins, Houston, Tasper, Morris, Polk, Red River, Rusa, Sabine, San Augustine, Shelby, Smith, Titus. See Search Criteria below for details. All other sources were transcribed in their entirety.

10.1 Search criteria

We searched for publications from the U.S. Geological Survey that were available online in the U.S. Geological Survey Publication Warehouse. We specifically looked for water level measurements that were made prior to 1910 and recorded in a tabular format.

Our pre-1910 data compilation is not comprehensive. There are other early well water level measurements available in different U.S. Geological Survey publications which we did not hand digitize due to limited time and resources. For the purpose of our study, we sought early records that spanned the continental US (i.e., (*60*), which contains measurements from all 48 continental US states), as well as some documents that focused on regional aquifer systems (i.e., (*3*), which focuses on the Mississippi Embayment Regional Aquifer System).

10.2 Transcription quality check

Five students at the University of California, Santa Barbara helped to manually transcribe the early 1900s well data (see Acknowledgements in the main text). Students were instructed to follow similar data compilation protocols. The data were transcribed, checked twice by the transcriber, then subjected to a final review for their accuracy by lead-author A.E. Hilton.

10.3 Data cleaning

The following modifications were made to the original text during transcription:

(i) punctuation marks in text (e.g., "?", "!", "") were removed

(ii) fractions were replaced with their decimal equivalent (e.g., ½ becomes 0.5)

(iii) blank columns indicated by punctuation (e.g., "........") were replaced with NA

(iv) "do" in text indicated "same as above" and was replaced by text preceding it

(v) columns were renamed for brevity and to be consistent with other data sources (see Columns below)

10.4 Columns

Table S24. Original and modified columns of early 1900s data (*3*, *57–60*).

Data Source	Original Column	Modified Column *
(*3*)	No	id
	State	state
	County	county
	Town	town
	Owner	owner
	Township	township
	Range	range
	Section	section
	Year completed	year
	Diameter of well	diameter_in
	Depth of well	min_depth_ft
		max_depth_ft
		depth_sign

Data Source	Original Column	Modified Column *
		depth_text
	Depth to principal water supply	depth_water_supply
	Height of water above (+) or below (-) mouth of well	min_height_water_ft
		max_height_water_ft
	How obtained at surface	obtained
	Quality	quality
	Supply per minute	yield_per_min_gal_pump
	Increase or decrease of supply	increase_decrease_supply
	Effect of pumping on level of water	pumping_effect
	Geologic horizon of well mouth	geologic_well_mouth
	Geologic horizon of principal water-bearing stratum	geologic_water
		citation
		page_no
(*60*)		id
	Location	state
		county
		city
		specific_location
	Depth	min_depth_ft

**Data Source**	**Original Column**	**Modified Column ***
		max_depth_ft
		depth_sign
	Diameter	diameter_in
	Yield per minute	yield_per_minute_gallons
	Height of water	min_height_water_ft
		max_height_water_ft
		height_water_text
	Remarks	remarks
		citation
		page_no
(*57*)	No	id
	Location	state
	County	county
		city
		specific_location
	T	township
	R	range
	S	section
	Owner	owner

Data Source	Original Column	Modified Column *
	Contractor	contractor
	Driller	driller
	Authority	authority
	Depth	min_depth_ft
		max_depth_ft
		depth_sign
		depth_text
	Diameter	diameter_in
	Depth to principal water or oil supply	depth_water_supply
	Height of water	min_height_water_ft
		max_height_water_ft
		height_water_text
	Yield per minute Flow	yield_per_min_gal_flow
	Yield per minute Pump	yield_per_min_gal_pump
	Year completed	year
	Kind of well	kind_of_well
	Remarks	remarks
		citation
		page_no

**Data Source**	**Original Column**	**Modified Column ***
(*59*)	No	id
	Kind of well	well_type
	Location	state
	County	county
		city
		specific_location
	Owner	owner
	Contractor	contractor
	Driller	driller
	Authority	authority
	Depth	min_depth_ft
		max_depth_ft
		depth_sign
		depth_text
	Diameter	diameter_in
	Depth to principal water or oil supply	depth_water_supply
	Height of water	min_height_water_ft
		max_height_water_ft
		height_water_text

**Data Source**	**Original Column**	**Modified Column ***
	Yield per minute Flow	yield_per_min_gal_flow
	Yield per minute Pump	yield_per_min_gal_pump
	Year completed	year
	Remarks	remarks
		citation
		page_no
(*58*)	No	id
	Location	state
		county
		city
		specific_location
		township
		range
		section
	Owner	owner
	Driller	driller
	Authority	authority
	Diameter of well	diameter_in
	Depth of well	min_depth_ft

Data Source	Original Column	Modified Column *
		max_depth_ft
		depth_sign
		depth_text
	Approximate elevation of surface	elevation_ft
	Height of water above (+) or below (-) the ground	min_height_water_ft
		max_height_water_ft
		height_water_text
	Depths of principal water bearing strata	min_depth_ft_strata
		max_depth_ft_strata
	Yield per minute Flow	yield_per_min_gal_flow
	Yield per minute Pump	yield_per_min_gal_pump
	Geologic horizon of water bearing strata	geologic
	Quality	quality
	Remarks	remarks
		citation
		page_no

*All headings are shorthand for the original column headings; some headings were added for clarity. Multiple headings for location were added if there was more than one locator description (e.g., separating “city”, “state”, “township”, “range”, “section”, and any shorthand location descriptions as “specific_location”). Multiple headings were also created to accurately track original data entries and include units of measurement, which often included numerical ranges, text, and special characters. These modifications include: (i) “Depth of well” which becomes “min_depth_ft”, “max_depth_ft”, “depth_sign”, “depth_text”; (ii) “Height of water above (+) or below (-) the ground” which becomes “min_height_water_ft”, “max_height_water_ft”, “height_water_text”; (iii) “Depths of principal water bearing strata” becomes “min_depth_ft_strata”, “max_depth_ft_strata”; (iv) “citation” and (v) “page_no” headings were added to denote the citation of data source and the page number of the data reference.

Table S25. Columns of combined early 1900s data (*3*, *57–60*).

**Complete early 1900s records**	id
	state
	county
	city
	specific_location
	township
	range
	section
	latitude*
	longitude*
	min_depth_ft
	max_depth_ft
	depth_sign
	depth_text
	min_height_water_ft
	max_height_water_ft
	height_water_text
	remarks

	citation

*columns added with geolocation

10.5 Geolocation

No wells in the early 1900s US Geological Survey reports have an associated latitude and longitude coordinate recorded within the report; the location of these wells are often provided as a description (e.g. State, County, City) or a township, range, and section. We estimated the latitude and longitude coordinates of each unique well by the following steps:

(1) Remove wells with no location information.
(2) Separate wells with only township, range, section (no city, county, state information)
(3) Use “Geocode by Awesome Table” (*68*) software to locate based on city, state column for remaining wells.
(4) Check all geolocations created in step 3 for accuracy, correct NA values or locations unable to be found.
(5) For wells with only a township, range, and section (step 2), locate township, range, section and calculate latitude and longitude based on the centroid of that township, range, section.
(6) For wells with a "specific location" (additional location information beyond township, range, section, city, county, state), estimate specific location if possible and add latitude/longitude estimation. For example, a “specific location” (as defined here) might read as follows: “6m nw of”. We used the geocoding software to estimate the landmark or city of the well (e.g., "Jackson, Alabama”) and then applied the horizontal offset (e.g., 6 miles north west of Jackson, Alabama) to estimate the latitude and longitude of the well.

10.6 Artesian indications

In the pre-1910 dataset, the depth to water measurements for each well are not described consistently. For example, some depth to water measurements are recorded as "below the mouth of the well," "below land surface", or simply as "height of water". We highlight that different early 1900s reports have different measurement reference points and/or measurement practices.

Table S26. Artesian conditions were indicated either by a positive number (+) or written text.

**Sign**	+
**Text**	"flow", "flows", "one flows", "flowed at first", "flowed", "flowed once", "slight flow", "flowed originally", "to surface", "nearly to surface", "rises to surface", "near surface", "surface", "at top", "nearly to top"

10.7 Data used in analysis

Data used in this study primarily included well location (estimated latitude and longitude), depth of well, height of water, and an artesian indication (by text or sign). Where ranges existed for well depth and height of water (see Columns above, e.g., indicated by "min_depth_ft", "max_depth_ft"), we utilized the maximum value.

We excluded well water level measurements from further analyses if (a) the well was identified as an oil and gas well, (b) the record did not specify a numeric well depth, (c) there was no recorded water level measurement or water depth indication, or (d) the recorded well location could not be straightforwardly converted into an estimate of the latitude and longitude, such as short-hand locations (e.g., Spence Run, Allegheny County, Pennsylvania).

10.8 Notes of limitations

Sometimes wells recorded in these historical documents are not one specific well, but are meant to represent multiple wells. Among the reports we consulted, this notation (i.e., multiple wells captured by one row in a table) is most common in (*60*), pg. 7 where it is stated "In regions of oil and gas wells, where borings are numerous, the individual wells cannot be listed here, but representative wells are given."

S11. Regional aquifer systems confined criteria & error analysis

In our regional aquifer system analysis, we analyzed hydrostratigraphic spatial data to determine the depth to confined conditions (see S3 for data sources). Well bottom elevations were used to determine the confinement category (i.e., either 'confined' or 'not confined') for all wells for each of our two studied time intervals: pre-1910 and post-2010. In combination with the spatial hydrostratigraphic data, our specific confining criteria for each aquifer system is described in Table S27.

In addition to our classification criteria with hydrostratigraphic data, we also identified US Geological Survey wells that were designated as being drilled in a confined or unconfined aquifer unit (n=22,946), which represent less than half (43.6%) of the wells in our post-2010 dataset; these US Geological Survey classifications were only available for our post-2010 dataset. For aquifer systems that had sufficient wells with a US Geological Survey confined classification (i.e., at least n=10 USGS wells where a confinement classification was available), we compared the results of our hydrostratigraphic confining classification to these data and calculated the error rate of our analysis (i.e., how our method of classifying confined wells matched the US Geological Survey's own classification of confined wells; see Table S28).

Table S27. Confining criteria for regional aquifer systems

Aquifer System	Confining criteria	Reference	Quote from reference
Columbia Plateau Regional Aquifer System	The bottom of a well must have a confining unit above it	(*74*)	"On a regional scale, it is assumed that interbeds transmit water much less efficiently than the CRBG aquifers and commonly are classified as 'confining units.'"
Dakota Aquifer System	The bottom of a well must be below the top of the Dakota aquifer unit	(*86*)	"The confining layer overlying the Dakota, which we call the 'Cretaceous shale confining layer,' includes the entire sequence above the Dakota-Newcastle Sandstone"

Aquifer System	Confining criteria	Reference	Quote from reference
North Atlantic Coastal Plain Aquifer System	The bottom of a well must have a confining unit above it	(*98*)	"The sediments of the NACP have been divided for this study into 10 regional aquifers (fig. 9) and 9 regional confining units. These divisions are based on similarities and differences in hydrologic characteristics resulting from geologic origins of the units. The regional hydrogeologic units defined here are typically groupings of aquifers or confining units previously recognized at the State or local scale, with correlations across State boundaries based primarily on the continuity of hydraulic permeability."
Floridan Aquifer System	The bottom of a well must have a confining unit above it	(*99*)	"Although the term 'confining unit' is not totally abandoned within the revised framework, a new term 'composite unit' is introduced for lithostratigraphic units that cannot be defined as either a confining or aquifer unit over their entire extent."
Mississippi Embayment Regional Aquifer	The bottom of a well must have a confining unit above it	(*77*)	"These units include Quaternary age deposits of the Mississippi River Valley alluvial aquifer (composed mainly of coarse gravel, sand, and clay), the Tertiary-age deposits of the Vicksburg-Jackson confining unit, upper Claiborne aquifer, middle Claiborne confining unit, middle Claiborne aquifer, lower Claiborne confining unit, lower Claiborne aquifer, middle Wilcox aquifer, lower Wilcox aquifer, and the Midway confining unit. These units make up the aquifers and the confining units of the Mississippi embayment aquifer system."
Houston-Gulf Coast Aquifer System	The bottom of a well must be 100 meters or greater in depth	(*104*)	"As depth increases in the aquifer system and the cumulative thicknesses of the interbedded sand and clay increase, water-table conditions transition to confined potentiometric conditions. Thus, the lowermost parts of the aquifer system (deep zones) are under confined conditions. The middle parts of the aquifer system (intermediate zones) therefore are under semiconfined conditions. Because the transition from water table to confined conditions incrementally increases with depth, assigning specific depth horizons to shallow, intermediate, and deep zones is problematic."

**Aquifer System**	**Confining criteria**	**Reference**	**Quote from reference**
Roswell Artesian Basin	Wells must be in the Artesia aquifer unit, and be contained between 10 km west of city of Roswell and the Pecos River	(*24*)	"The artesian aquifer becomes confined 10 km west of the city of Roswell, where the eastward-dipping San Andres limestone passes beneath gypsum and mudstones of the Artesia Group."
Central Valley Aquifer	The bottom of a well must be 150 meters or greater in depth	(*41*)	See Supplementary Note 3 in (*41*): Sections 3.1, 3.2, 3.3 for depth to confined conditions

Table S28. Error analysis of confined conditions with US Geological Survey designations

Aquifer System	Total confined wells	Total wells with USGS classification	Correctly confined according to USGS	Incorrectly confined according to USGS	Error
Columbia Plateau Regional Aquifer System	105	7	4	3	NA*
Dakota Aquifer System	64	5	5	0	NA*
North Atlantic Coastal Plain Aquifer System	1730	1348	1286	62	4.6 %
Floridan Aquifer System	1037	458	445	13	2.8 %
Mississippi Embayment Regional Aquifer	700	486	459	27	5.6 %
Houston-Gulf Coast Aquifer System	765	577	440	137	23.7 %
Roswell Artesian Basin	32	1	1	0	NA*
Central Valley Aquifer	512	18	16	2	11.1 %
Continental United States	7770	3903	3567	336	8.6 %

*NA indicates not sufficient sample size (n < 10) of US Geological Survey wells with confined designation to conduct error analysis

REFERENCES AND NOTES

1. T. N. Narasimhan, Groundwater: From mystery to management. *Environ. Res. Lett.* **4**, 035002 (2009).

2. W. C. Mendenhall, R. B. Dole, H. Stabler, Ground water in San Joaquin Valley, California. *USGS Water Supply Paper* (no. 398) (1916).

3. A. F. Crider, L. C. Johnson, Summary of the underground-water resources of Mississippi. *USGS Water Supply Paper* (no. 159) (1906).

4. H. L. Young, D. I. Siegel, Hydrogeology of the Cambrian-Ordovician aquifer system in the northern Midwest, United States with a section on ground-water quality. *USGS Professional Paper* (no. 1405-B) (1992).

5. A. G. Fiedler, Artesian water in Somervell County, Texas. *USGS Water Supply Paper* (no. 660) (1934).

6. E. Chaussard, E. Havazli, H. Fattahi, E. Cabral-Cano, D. Solano-Rojas, Over a century of sinking in Mexico City: No hope for significant elevation and storage capacity recovery. *J. Geophys. Res. Solid Earth*. **126**, e2020JB020648 (2021).

7. T. D. Lahm, E. S. Bair, Regional depressurization and its impact on the sustainability of freshwater resources in an extensive midcontinent variable-density aquifer. *Water Resour. Res.* **36**, 3167–3177 (2000).

8. G. Herrera-García, P. Ezquerro, R. Tomás, M. Béjar-Pizarro, J. López-Vinielles, M. Rossi, R. M. Mateos, D. Carreón-Freyre, J. Lambert, P. Teatini, E. Cabral-Cano, G. Erkens, D. Galloway, W.-C. Hung, N. Kakar, M. Sneed, L. Tosi, H. Wang, S. Ye, Mapping the global threat of land subsidence. *Science* **371**, 34–36 (2021).

9. J. Tóth, Mapping and interpretation of field phenomena for groundwater reconnaissance in a prairie environment, Alberta, Canada. *Int. Assoc. Sci. Hydrol. Bull.* **11**, 20–68 (1966).

10. J.-Z. Wang, X.-W. Jiang, L. Wan, A. Wörman, H. Wang, X.-S. Wang, H. Li, An analytical study on artesian flow conditions in unconfined-aquifer drainage basins. *Water Resour. Res.* **51**, 8658–8667 (2015).

11. Y.-P. Zhang, X.-W. Jiang, J. Cherry, Z.-Y. Zhang, X.-S. Wang, L. Wan, Revisiting hydraulics of flowing artesian wells: A perspective from basinal groundwater hydraulics. *J. Hydrol.* **609**, 127714 (2022).

12. X.-W. Jiang, J. Cherry, L. Wan, Flowing wells: Terminology, history and role in the evolution of groundwater science. *Hydrol. Earth Syst. Sci.* **24**, 6001–6019 (2020).

13. M. L. Fuller, Bibliographic review and index of papers relating to underground waters. *USGS Water Supply Paper* (no. 120) (1905).

14. D. J. MacAllister, G. Krishan, M. Basharat, D. Cuba, A. M. MacDonald, A century of groundwater accumulation in Pakistan and northwest India. *Nat. Geosci.* **15**, 390–396 (2022).

15. L. F. Konikow, Groundwater depletion in the United States (1900−2008). *Scientific Investigations Report* (USGS Numbered Series 2013–5079, U.S. Geological Survey, Reston, VA, 2013), p. 75.

16. Ostrom, *Water & politics; a study of water policies and administration in the development of Los Angeles* (Haynes Foundation, Los Angeles, 1953).

17. W. C. Mendenhall, Development of underground waters in the central coastal plain region of southern California. *USGS Water Supply Paper* (no. 138) (1905).

18. Leverett, Flowing wells and municipal water supplies in the southern portion of the southern peninsula of Michigan. *USGS Water Supply Paper* (no. 182) (1906).

19. J. D. Whitney, *The United States: Facts and Figures Illustrating the Physical Geography of the Country, and Its Material Resources; Supplement I. Population, Immigration, Irrigation* (Little, Brown, 1894).

20. A. Baiocchi, F. Lotti, V. Piscopo, Impact of groundwater withdrawals on the interaction of multi-layered aquifers in the Viterbo geothermal area (central Italy). *Hydrgeol. J.* **21**, 1339–1353 (2013).

21. C. A. Dieter, M. A. Maupin, R. R. Caldwell, M. A. Harris, T. I. Ivahnenko, J. K. Lovelace, N. L. Barber, K. S. Linsey, Estimated use of water in the United States in 2015. *USGS Circular* (no. 1441) (2018).

22. J. Margat, J. van der Gun, *Groundwater around the World: A Geographic Synopsis* (CRC Press, 2013).

23. T. C. Chamberlin, The Requisite and Qualifying Conditions of Artesian Wells. *USGS Fifth Annual Report of the Director* (1885).

24. L. Land, B. T. Newton, Seasonal and long-term variations in hydraulic head in a karstic aquifer: Roswell artesian Basin, new Mexico1. *JAWRA J. Am. Water Resour. Assoc.* **44**, 175–191 (2008).

25. D. L. Galloway, D. R. Jones, S. E. Ingebritsen, Land Subsidence in the United States. *USGS Circular* (no. 1182) (1999).

26. H. G. Healy, Appraisal of uncontrolled flowing artesian wells in Florida. *USGS Water-Resources Investigations Report* (no. 95) (1978).

27. J. Evans, The abandoned artesian well plugging program. *St Johns River Water Manag. Dist.*, (available at https://sjrwmd.com/education/abandoned-wells/).

28. Artesian Well Rebate Program. *Coach. Val. Water Dist.*, (available at http://cvwd.org/204/Artesian-Well-Rebate-Program).

29. E. Salameh, A. Tarawneh, Assessing the impacts of uncontrolled artesian flows on the management of groundwater resources in the Jordan Valley. *Environ. Earth Sci.* **76**, 291 (2017).

30. M. Velis, K. I. Conti, F. Biermann, Groundwater and human development: Synergies and trade-offs within the context of the sustainable development goals. *Sustain. Sci.* **12**, 1007–1017 (2017).

31. C. Walsh, Hydraulic opulence: Artesian wells and bathing in Mexico, 1850–1900. *Water Hist.* **14**, 85–100 (2022).

32. J. H. Waterman, *General history of Seward County, Nebraska* (Beaver Crossing, Nebr, 1916).

33. N. H. Darton, Preliminary report on the geology and underground water resources of the central Great Plains. *USGS Professional Paper* (no. 149) (1905).

34. C. A. Fisher, Preliminary report on the geology and underground waters of the Roswell artesian area, New Mexico. *USGS Professional Paper* (no. 158) (1906).

35. N. H. Darton, Artesian-well prospects in the Atlantic Coastal Plain region. *US Department of the Interior Bulletin* (no. 138) (1896).

36. C. Cutter, Cutter's Guide to the City of Waco, Texas. *Padgitts Park Natatorium* (1894; https://digitalcollections-baylor.quartexcollections.com/Documents/Detail/cutters-guide-to-the-city-of-waco-texas/936413?item=936443).

37. B. Simmons M., Geyser City, Waco: Reading a Photograph of the Crystal Palace Pool. *Tex. Collect. Bayl. Univ.* (2012; https://blogs.baylor.edu/texascollection/2012/08/01/geyser-city-waco-reading-photograph-crystal-palace-pool/).

38. E. H. Barbour, Wells and windmills in Nebraska. *USGS Water Supply Paper* (no. 29) (1899).

39. Lyle, Artesian Wells: Technology That Changed Chicago. *Chic. Public Libr.* (2015; https://chipublib.org/blogs/post/technology-that-changed-chicago-artesian-wells).

40. J. B. Smith, Waco a "Geyser City" no more. *Waco Trib.-Her. TX* (2010; https://wacotrib.com/news/waco-a-geyser-city-no-more/article_90a7719f-a723-5905-89b4-a672c22d96fb.html).

41. M. Thaw, M. GebreEgziabher, J. Y. Villafañe-Pagán, S. Jasechko, Modern groundwater reaches deeper depths in heavily pumped aquifer systems. *Nat. Commun.* **13**, 5263 (2022).

42. M. Falkenmark, C. Folke, S. S. D. Foster, P. J. Chilton, Groundwater: The processes and global significance of aquifer degradation. *Philos. Trans. R. Soc. Lond. B Biol. Sci.* **358**, 1957–1972 (2003).

43. K. B. Brown, J. C. McIntosh, L. K. Rademacher, K. A. Lohse, Impacts of agricultural irrigation recharge on groundwater quality in a basalt aquifer system (Washington, USA): A multi-tracer approach. *Hydrgeol. J.* **19**, 1039–1051 (2011).

44. R. Smith, R. Knight, S. Fendorf, Overpumping leads to California groundwater arsenic threat. *Nat. Commun.* **9**, 2089 (2018).

45. I. Mihajlov, M. R. H. Mozumder, B. C. Bostick, M. Stute, B. J. Mailloux, P. S. K. Knappett, I. Choudhury, K. M. Ahmed, P. Schlosser, A. van Geen, Arsenic contamination of Bangladesh aquifers exacerbated by clay layers. *Nat. Commun.* **11**, 2244 (2020).

46. J. D. Ayotte, Z. Szabo, M. J. Focazio, S. M. Eberts, Effects of human-induced alteration of groundwater flow on concentrations of naturally-occurring trace elements at water-supply wells. *Appl. Geochem.* **26**, 747–762 (2011).

47. R. K. Gabrysch, C. W. Bonnet, Land-surface subsidence in the area of Moses Lake near Texas City, Texas. *USGS Water-Resources Investigations Report* (no. 76–32) (1976).

48. G. H. Davis, J. B. Small, H. B. Counts, Land Subsidence Related to Decline of Artesian Pressure in the Ocala Limestone at Savannah, Georgia, in *Engineering Geology Case Histories Number 4*, P. D. Trask, G. A. Kiersch, Eds. Geological Society of America (1963).

49. A. Brambati, L. Carbognin, T. Quaia, P. Teatini, L. Tosi, The Lagoon of Venice: Geological setting, evolution and land subsidence. *Episodes J. Int. Geosci.* **26**, 264–268 (2003).

50. J. Poland, *Guidebook to Studies on Land Subsidence due to ground-water withdrawal* (The United Nations, Paris, France, 1984; https://rcamnl.wr.usgs.gov/rgws/Unesco/).

51. P. Sahu, P. K. Sikdar, Threat of land subsidence in and around Kolkata City and East Kolkata Wetlands, West Bengal, India. *J. Earth Syst. Sci.* **120**, 435–446 (2011).

52. C. C. Faunt, M. Sneed, J. Traum, J. T. Brandt, Water availability and land subsidence in the Central Valley, California, USA. *Hydrogeol. J.* **24**, 675–684 (2016).

53. PRISM Gridded Climate Data. *PRISM Clim. Group Or. State Univ.* at https://prism.oregonstate.edu/normals/).

54. R. J. Zomer, J. Xu, A. Trabucco, Version 3 of the global aridity index and potential evapotranspiration database. *Sci. Data*. **9**, 409 (2022).

55. L. F. Konikow, E. Kendy, Groundwater depletion: A global problem. *Hydrgeol. J.* **13**, 317–320 (2005).

56. A. C. Amanambu, O. A. Obarein, J. Mossa, L. Li, S. S. Ayeni, O. Balogun, A. Oyebamiji, F. U. Ochege, Groundwater system and climate change: Present status and future considerations. *J. Hydrol.* **589**, 125163 (2020).

57. M. L. Fuller, E. F. Lines, A. C. Veatch, Record of deep-well drilling for 1904. *USGS Bulletin* (no. 264) (1905).

58. A. C. Veatch, Geology and underground water resources of northern Louisiana and southern Arkansas. *USGS Professional Paper* (no. 46) (1906).

59. M. L. Fuller, S. Sanford, Record of deep-well drilling for 1905. *USGS Bulletin* (no. 298) (1906).

60. N. H. Darton, Preliminary list of deep borings in the United States. *USGS Water Supply Paper* (no. 149) (1905).

61. R. A. Pauloo, A. Escriva-Bou, H. Dahlke, A. Fencl, H. Guillon, G. E. Fogg, Domestic well vulnerability to drought duration and unsustainable groundwater management in California's Central Valley. *Environ. Res. Lett.* **15**, 044010 (2020).

62. B. Walton, California's Dogged Drought Cutting Off Water Supplies to State's Poor. *Circ. Blue* (2014; https://circleofblue.org/2014/world/californias-dogged-drought-cutting-water-supplies-states-poor/).

63. S. Jasechko, D. Perrone, California's Central Valley groundwater wells run dry during recent drought. *Earths Future* **8**, e2019EF001339 (2020).

64. R. Becker, California enacted a groundwater law 7 years ago. But wells are still drying up — and the threat is spreading. *CalMatters* (2021; http://calmatters.org/environment/2021/08/california-groundwater-dry/).

65. S. Cagle, "Lost communities": thousands of wells in rural California may run dry. *The Guardian* (2020; https://theguardian.com/environment/2020/feb/28/california-water-wells-dry-sgma).

66. USGS Water Data for the Nation. *U. S. Geol. Surv.*, https://waterdata.usgs.gov/nwis).

67. Water-Level Status Codes. *U. S. Geol. Surv.* (2011 https://help.waterdata.usgs.gov/code/lev_status_query?fmt=html).

68. Geocode by Awesome Table (2021; https://workspace.google.com/marketplace/app/geocode_by_awesome_table/904124517349).

69. J. A. Hansen, B. C. Jurgens, M. S. Fram, Groundwater-quality data and ancillary data for selected wells in the San Joaquin Valley, California, 1900-2015. US Geological Survey data release 10.5066/F7319T3K (2018).

70. Groundwater Ambient Monitoring and Assessment Program (GAMA). *Calif. State Water Resour. Control Board* (2021; https://waterboards.ca.gov/gama/).

71. Periodic Groundwater Level Measurements-California Natural Resources Agency Open Data, (https://data.cnra.ca.gov/dataset/periodic-groundwater-level-measurements).

72. Continuous Groundwater Level Measurements-California Natural Resources Agency Open Data, (https://data.cnra.ca.gov/dataset/continuous-groundwater-level-measurements).

73. Observation Wells. *S. D. Dep. Agric. Nat. Resour.*, (https://apps.sd.gov/nr69obswell/default.aspx#viewHelp).

74. E. R. Burns, D. S. Morgan, R. S. Peavler, S. C. Kahle, Three-Dimensional Model of the Geologic Framework for the Columbia Plateau Regional Aquifer System, Idaho, Oregon, and Washington. *USGS Scientific Investigations Report* (no. 5246) (2010).

75. J. P. Pope, D. C. Andreasen, E. R. Mcfarland, M. K. Watt, Digital elevations and extents of regional hydrogeologic units in the Northern Atlantic Coastal Plain aquifer system from Long Island, New York, to North Carolina. *USGS Data Series* (no. 996) (2016).

76. L. J. Williams, J. F. Dixon, Digital surfaces and thicknesses of selected hydrogeologic units of the Floridan aquifer system in Florida and parts of Georgia, Alabama, and South Carolina. *USGS Data Series* (no. 926) (2015).

77. R. M. Hart, B. R. Clark, S. E. Bolyard, Digital Surfaces and Thicknesses of Selected Hydrogeologic Units within the Mississippi Embayment Regional Aquifer Study (MERAS). *USGS Scientific Investigations Report* (no. 5098) (2008).

78. M. C. Kasmarek, Hydrogeology and simulation of groundwater flow and land-surface subsidence in the northern part of the Gulf Coast aquifer system, Texas, 1891-2009. *USGS Scientific Investigations Report* (no. 5154) (2012).

79. C. Cikoski, M. Fichera, E. Mamer, L. Sturgis, A Three-Dimensional Hydrogeologic Model from the Pecos Slope to the Southern High Plains, Southeastern New Mexico. *New Mexico Bureau of Geology and Mineral Resources* (Open-File Report 614) (2020).

80. C. Faunt, Groundwater availability in the Central Valley. *USGS Professional Paper* (no. 1766) (2009).

81. Lithologic Logs Database. *S. D. Dep. Agric. Nat. Resour.*, (http://cf.sddenr.net/lithdb/).

82. GIS Data Download the National Map. *U. S. Geol. Surv.*, (available at https://usgs.gov/the-national-map-data-delivery/gis-data-download?qt-science_support_page_related_con=0#qt-science_support_page_related_con).

83. M. GebreEgziabher, S. Jasechko, D. Perrone, Widespread and increased drilling of wells into fossil aquifers in the USA. *Nat. Commun.* **13**, 2129 (2022).

84. R. A. Freeze, J. A. Cherry, *Groundwater* (Prentice-Hall, Englewood Cliffs, N.J, 1979).

85. S. C. Kahle, D. S. Morgan, W. B. Welch, D. M. Ely, S. R. Hinkle, J. J. Vaccaro, L. L. Orzol, Hydrogeologic Framework and Hydrologic Budget Components of the Columbia Plateau Regional Aquifer System, Washington, Oregon, and Idah. *USGS Scientific Investigations Report* (no. 5124) (2011).

86. J. D. Bredehoeft, C. E. Neuzil, P. C. Milly, Regional flow in the Dakota aquifer; a study of the role of confining layers. *USGS Water Supply Paper* (no. 2237) (1983).

87. J. P. Masterson, J. P. Pope, M. N. Fienen, J. Monti Jr., M. R. Nardi, J. S. Finkelstein, Assessment of groundwater availability in the Northern Atlantic Coastal Plain aquifer system From Long Island, New York, to North Carolina. *USGS Professional Paper* (no. 1829) (2016).

88. L. J. Williams, H. E. Gill, Revised Hydrogeologic Framework of the Floridan Aquifer System in the Northern Coastal Area of Georgia and Adjacent Parts of South Carolina. *USGS Scientific Investigations Report* (no. 5158) (2010).

89. R. A. Renken, Ground Water Atlas of the United States: Segment 5, Arkansas, Louisiana, Mississippi. *USGS Numbered Series Hydrologic Atlas* (no. 730-F) (1998).

90. C. L. Braun, J. K. Ramage, S. D. Shah, Status of groundwater-level altitudes and long-term groundwater-level changes in the Chicot, Evangeline, and Jasper aquifers, Houston-Galveston region, Texas, 2019. *USGS Scientific Investigations Report* (no. 5089) (2019).

91. R. W. Page, G. O. Balding, Geology and quality of water in the Modesto-Merced area, San Joaquin Valley, California, with a brief section on hydrology. *USGS Water-Resources Investigations Report* (no. 6) (1973).

92. C. W. Myers, S. M. Price, Geologic studies of the Columbia Plateau: A status report. (RHO-BWI-ST-4, Rockwell International Corp., Richland, WA (USA). Rockwell Hanford Operations, 1979; https://osti.gov/biblio/6681240).

93. J. L. Riedel, A. Telka, A. Bunn, J. J. Clague, Reconstruction of climate and ecology of Skagit Valley, Washington, from 27.7 to 19.8 ka based on plant and beetle macrofossils. *Quatern. Res.* **106**, 94–112 (2022).

94. J. J. Vaccaro, S. C. Kahle, D. M. Ely, E. R. Burns, D. T. Snyder, J. V. Haynes, T. D. Olsen, W. B. Welch, D. S. Morgan, Groundwater availability of the Columbia Plateau Regional Aquifer System, Washington, Oregon, and Idaho. *USGS Professional Paper* (no. 1817) (2015).

95. E. R. Burns, Groundwater Status and Trends for the Columbia Plateau Regional Aquifer System, Washington, Oregon, and Idaho. *USGS Scientific Investigations Report* (no. 5261) (2012).

96. D. M. Ely, E. R. Burns, D. S. Morgan, J. J. Vaccaro, Numerical simulation of groundwater flow in the Columbia Plateau Regional Aquifer System, Idaho, Oregon, and Washington. *USGS Scientific Investigations Report* (no. 5127) (2014).

97. L. F. Konikow, C. E. Neuzil, A method to estimate groundwater depletion from confining layers. *Water Resour. Res.* **43**, W07417 (2007).

98. J. P. Masterson, J. P. Pope, J. Monti Jr., M. R. Nardi, J. S. Finkelstein, K. J. McCoy, Hydrogeology and hydrologic conditions of the Northern Atlantic Coastal Plain aquifer System from Long Island, New York, to North Carolina. *USGS Scientific Investigations Report* (no. 5133) (2015).

99. L. J. Williams, E. L. Kuniansky, Revised hydrogeologic framework of the Floridan aquifer system in Florida and parts of Georgia, Alabama, and South Carolina. *USGS Professional Paper* (no. 1807) (2015).

100. J. C. Bellino, E. L. Kuniansky, A. M. O'Reilly, J. F. Dixon, Hydrogeologic setting, conceptual groundwater flow system, and hydrologic conditions 1995–2010 in Florida and parts of Georgia, Alabama, and South Carolina. *USGS Scientific Investigations Report* (no. 5030) (2018).

101. E. L. Kuniansky, J. C. Bellino, Tabulated Transmissivity and Storage Properties of the Floridan Aquifer System in Florida and Parts of Georgia, South Carolina, and Alabama. *USGS Data Series* (no. 669) (2016).

102. E. M. Cushing, E. H. Boswell, R. L. Hosman, General geology of the Mississippi embayment. *USGS Professional Paper* (no. 448-B) (1964).

103. B. R. Clark, R. M. Hart, The Mississippi Embayment Regional Aquifer Study (MERAS): Documentation of a groundwater-flow model constructed to assess water availability in the Mississippi embayment. *USGS Scientific Investigations Report* (no. 5172) (2009).

104. M. C. Kasmarek, J. L. Robinson, Hydrogeology and simulation of ground-water flow and land-surface subsidence in the northern part of the Gulf Coast aquifer system, Texas. *USGS Scientific Investigations Report* (no. 5102) (2004).

105. G. L. Bertoldi, R. H. Johnston, K. D. Evenson, Ground water in the Central Valley, California; a summary report. *USGS Professional Paper* (no. 1401-A) (1991).

106. R. L. Ireland, J. F. Poland, F. S. Riley, Land subsidence in the San Joaquin Valley, California, as of 1980. *USGS Professional Paper* (no. 437-I) (1984).

107. J. P. Masterson, J. P. Pope, J. Monti Jr., M. R. Nardi, J. S. Finkelstein, K. J. McCoy, Hydrogeology and hydrologic conditions of the Northern Atlantic Coastal Plain aquifer System from Long Island, New York, to North Carolina. *USGS Scientific Investigations Report* (no. 5133) (2013).

108. B. R. Clark, R. M. Hart, J. J. Gurdak, Groundwater availability of the Mississippi Embayment. *USGS Professional Paper* (no. 1785) (2011).

109. M. D. Kozar, Geohydrology and ground-water quality of Southern Canaan Valley, Tucker County, West Virginia. *USGS Water-Resources Investigations Report* (no. 4103) (1996).

110. T. F. Buckwalter, M. E. Moore, Ground-Water Resources and the Hydrologic Effects of Petroleum Occurrence and Development, Warren County, Northwestern Pennsylvania. *USGS Scientific Investigations Report* (no. 5263) (2006).

111. B. J. O. L. McPherson, G. Garven, Hydrodynamics and overpressure mechanisms in the Sacramento Basin, California. *Am. J. Sci.* **299**, 429–466 (1999).

112. K. R. Burrow, J. L. Shelton, J. A. Hevesi, G. S. Weissmann, Hydrogeologic Characterization of the Modesto Area, San Joaquin Valley, California. *USGS Scientific Investigations Report* (no. 5232) (2004).

113. M. J. Stephens, D. H. Shimabukuro, J. M. Gillespie, W. Chang, Groundwater salinity mapping using geophysical log analysis within the Fruitvale and Rosedale Ranch oil fields, Kern County, California, USA. *Hydrogeol. J.* **27**, 731–746 731746 (2018).

114. A. R. Dutton, B. Harden, J. P. Nicot, D. O'Rourke, Groundwater availability model for the central part of the Carrizo-Wilcox aquifer in Texas. (Texas Water Development Board, 2003).

115. V. A. Kelley, N. E. Deeds, D. G. Fryar, J. P. Nicot, Groundwater Availability Models for the Queen City and Sparta Aquifers. (Texas Water Development Board, 2004), (available at https://twdb.texas.gov/groundwater/models/gam/qcsp/qcsp.asp).

116. M. A. Thomas, Arsenic in groundwater of Licking County, Ohio, 2012—Occurrence and relation to hydrogeology. *USGS Scientific Investigations Report* (no. 5148) (2016).

117. R. T. Kay, P. M. Buszka, Application of hydrogeology and groundwater-age estimates to assess the travel time of groundwater at the site of a landfill to the Mahomet Aquifer, near Clinton, Illinois. *USGS Scientific Investigations Report* (no. 5159) (2016).

118. L. D. Arihood, M. E. Basch, Geohydrology and simulated ground-water flow in an irrigated area of northwestern Indiana. *USGS Water-Resources Investigations Report* (no. 4046) (1994).

119. M. R. Risch, Hydrogeologic investigations by the U.S. Geological Survey at the former Fort Benjamin Harrison, Marion County, Indiana. *USGS Fact Sheet* (no. 99) (1999).

120. R. A. Barker, A. F. Ardis, Hydrogeologic framework of the Edwards-Trinity aquifer system, west-central Texas. *USGS Professional Paper* (no. 1421-B) (1996).

121. J. S. Clarke, D. C. Leeth, D. Taylor-Harris, J. A. Painter, J. L. Labowski, Summary of hydraulic properties of the Floridan Aquifer system in coastal Georgia and adjacent parts of South Carolina and Florida. *USGS Scientific Investigations Report* (no. 5264) (2004).

122. D. W. Hicks, H. E. Gill, S. A. Longsworth, Hydrogeology, chemical quality, and availability of ground water in the Upper Floridan aquifer, Albany area, Georgia. *USGS Water Resources Investigation Report* (no. 4145) (1987).

123. A. O'Reilly, R. Spechler, B. McGurk, Hydrogeologic and water-quality characteristics of the Lower Floridan aquifer in east-central Florida, (2002).

124. L. A. Knochenmus, Regional evaluation of the hydrogeologic framework, hydraulic properties, and chemical characteristics of the intermediate aquifer system underlying southern west-central Florida. *USGS Scientific Investigations Report* (no. 5013) (2006).

125. W. R. Aucott, Hydrology of the southeastern Coastal Plain aquifer system in South Carolina and parts of Georgia and North Carolina. *USGS Professional Paper* (no. 1410-E) (1996).

126. J. H. Davis, Hydrogeologic investigation and simulation of ground-water flow in the Upper Floridan Aquifer of north-central Florida and southwestern Georgia and delineation of contributing areas for selected city of Tallahassee, Florida, water-supply wells. *USGS Water-Resources Investigations Report* (no. 4296) (1996).

127. R. W. Fairchild, Availability of water in the Floridan aquifer in southern Duval and northern Clay and St. Johns counties, Florida. *USGS Water-Resources Investigations Report* (no. 98) (1976).

128. M. J. Mallory, Hydrogeology of the Southeastern Coastal Plain aquifer system in parts of eastern Mississippi and western Alabama. *USGS Professional Paper* (no. 1410-G) (1993).

129. K. J. Halford, N. L. Barber, Analysis of ground-water flow in the Catahoula aquifer system in the vicinity of Laurel and Hattiesburg, Mississippi. *USGS Water-Resources Investigations Report* (no. 4219) (1995).

130. L. B. Prakken, V. E. White, J. K. Lovelace, Water resources of Orleans Parish, Louisiana. *USGS Fact Sheet* (no. 3017) (2014).

131. R. B. Fendick Jr., J. M. Griffith, L. B. Prakken, Water Resources of Lafayette Parish. *USGS Fact Sheet* (no. 3048) (2011).

132. A. Buono, The Southern Hills regional aquifer system of southeastern Louisiana and southwestern Mississippi. *USGS Water-Resources Investigations Report* (no. 4189) (1983).

133. G. J. Leonard, K. R. Watts, Hydrogeology and simulated effects of ground-water development on an unconfined aquifer in the Closed Basin Division, San Luis Valley, Colorado. *USGS Water-Resources Investigations Report* (no. 4284) (1988).

134. E. Haile, A. E. Fryar, Chemical evolution of groundwater in the Wilcox aquifer of the northern Gulf Coastal Plain, USA. *Hydrogeol. J.* **25**, 2403–2418 (2017).

135. J. V. Brahana, T. O. Mesko, Hydrogeology and preliminary assessment of regional flow in the upper Cretaceous and adjacent aquifers in the northern Mississippi embayment. *USGS Water-Resources Investigations Report* (no. 4000) (1988).

136. H. L. Welch, J. A. Kingsbury, R. W. Tollett, R. C. Seanor, Quality of Shallow Groundwater and Drinking Water in the Mississippi Embayment-Texas Coastal Uplands Aquifer System and the Mississippi River Valley Alluvial Aquifer, South-Central United States, 1994-2004. *USGS Scientific Investigations Report* (no. 5091) (2009).

137. W. E. Sanford, J. P. Pope, D. L. Selnick, R. F. Stumvoll, Simulation of groundwater flow in the shallow aquifer system of the Delmarva Peninsula, Maryland and Delaware. *USGS Open-File Report* (no. 1140) (2012).

138. D. A. Vroblesky, W. B. Fleck, Hydrogeologic framework of the coastal plain of Maryland, Delaware, and the District of Columbia. *USGS Professional Paper* (no. 1404-E) (1991).

139. P. J. Lacombe, G. B. Carleton, Hydrogeologic framework, availability of water supplies, and saltwater intrusion, Cape May County, New Jersey. *USGS Water Resources Investigation Report* (no. 4246) (2002).

140. M. D. Winner Jr., R. W. Coble, Hydrogeologic framework of the North Carolina Coastal Plain aquifer system. *USGS Open-File Report* (no. 690) (1989).

141. A. J. Long, J. N. Thamke, K. W. Davis, T. T. Bartos, Groundwater availability of the Williston Basin, United States and Canada. *USGS Professional Paper* (no. 1841) (2018).

142. M. Gotkowitz, A. T. Leaf, S. M. Sellwood, Hydrogeology and simulation of groundwater flow in Columbia County, Wisconsin. *USGS Wisconsin Geological and Natural History Survey Bulletin* (2021).

143. M. E. Savoca, E. M. Sadorf, K. K. B. Akers, Ground-water quality in the eastern part of the Silurian-Devonian and upper Carbonate aquifers in the eastern Iowa basins, Iowa and Minnesota, 1996. *USGS Water-Resources Investigations Report* (no. 4224) (1999).

144. T. D. Conlon, Hydrogeology and simulation of ground-water flow in the Sandstone Aquifer, northeastern Wisconsin. *USGS Water-Resources Investigations Report* (no. 4096) (1998).

145. J. E. Almendinger, J. H. Leete, Regional and local hydrogeology of calcareous fens in the Minnesota River basin, USA. *Wetlands*. **18**, 184–202 (1998).

146. R. M. Yager, N. Plummer, L. J. Kauffman, D. H. Doctor, D. L. Nelms, P. Schlosser, Comparison of age distributions estimated from environmental tracers by using binary-dilution and numerical models of fractured and folded karst: Shenandoah Valley of Virginia and West Virginia USA. *Hydrogeol. J.* **21**, 25 (2013).

147. D. Kopaska-Merkel, L. Dean, *Hydrogeology and vulnerability to contamination of major aquifers in Alabama: Area 4* (2020).

148. M. V. Marcher, D. L. Bergman, L. J. Slack, S. P. Blumer, Hydrology of area 41, Western Region, Interior Coal Province, Oklahoma and Arkansas. *USGS Water-Resources Investigations Report* (no. 129) (1987).

149. D. G. Driscoll, J. M. Carter, J. E. Williamson, L. D. Putnam, Hydrology of the Black Hills Area, South Dakota. *USGS Water Resources Investigation Report* (no. 4094) (2002).

150. E. W. Strom, M. J. Mallory, Hydrogeology and simulation of ground-water flow in the Eutaw-McShan Aquifer and in the Tuscaloosa aquifer system in northeastern Mississippi. *USGS Water-Resources Investigations Report* (no. 4223) (1995).

151. H. F. Malenda, C. A. Penn, Groundwater levels in the Denver Basin bedrock aquifers of Douglas County, Colorado, 2011–19. *USGS Scientific Investigations Report* (no. 5076) (2020).

152. R. Rowden, Groundwater Resource Evaluation of the Lowa Dakota Aquifer in North-Central and Southwest Iowa. *Iowa Geological Survey Water-Resources Investigations Report* (no. 12) (2015).

153. P. G. Olcott, Ground Water Atlas of the United States: Segment 12, Connecticut, Maine, Massachusetts, New Hampshire, New York, Rhode Island, Vermont. *USGS Hydrologic Atlas* (no. 730–M) (1995).

154. D. B. Westjohn, T. L. Weaver, Hydrogeologic framework of the Michigan Basin regional aquifer system. *USGS Professional Paper* (no. 1418) (1998).

155. J. A. Miller, Ground Water Atlas of the United States: Segment 6, Alabama, Florida, Georgia, South Carolina. *USGS Hydrologic Atlas* (no. 730-G) (1990).

156. W. L. Lyke, A. R. Brockman, Ground-water pumpage and water-level declines in the Peedee and Black Creek aquifers in Onslow and Jones counties, North Carolina, 1900–86. *USGS Water-Resources Investigation Report* (no. 4197) (1990).

157. G. E. Welder, Geohydrologic framework of the Roswell ground-water basin, Chaves and Eddy Counties, New Mexico. *USGS Technical Report* (no. 42) (1983).

158. A. H. Manning, D. K. Solomon, An integrated environmental tracer approach to characterizing groundwater circulation in a mountain block. *Water Resour. Res.* **41**, W12412 (2005).

159. C. M. Wentworth, R. C. Jachens, R. A. Williams, J. C. Tinsley III, R. T. Hanson, Physical subdivision and description of the water-bearing sediments of the Santa Clara Valley, California. *USGS Scientific Investigations Report* (no. 5017) (2015).

160. D. Bates, M. Maechler, B. Bolker [aut, cre, S. Walker, R. H. B. Christensen, H. Singmann, B. Dai, F. Scheipl, G. Grothendieck, P. Green, J. Fox, A. Bauer, P. N. K. (shared copyright on simulate.formula), lme4: Linear Mixed-Effects Models using "Eigen" and S4 (2023; https://cran.r-project.org/web/packages/lme4/index.html).
